# PPAR-γ agonists reactivate the ALDOC-NR2F1 axis to enhance sensitivity to temozolomide and suppress glioblastoma progression

**DOI:** 10.1186/s12964-024-01645-3

**Published:** 2024-05-13

**Authors:** Yu-Chan Chang, Ming-Hsien Chan, Chien-Hsiu Li, Chi-Long Chen, Wen-Chiuan Tsai, Michael Hsiao

**Affiliations:** 1 Department of Biomedical Imaging and Radiological Sciences, National Yang Ming Chiao Tung University, Taipei, 112 Taiwan; 2 Department of Urology, Shuang Ho Hospital, Taipei Medical University, New Taipei, 235 Taiwan; 3 Department of Pathology, Taipei Medical University Hospital, Taipei Medical University, Taipei, 110 Taiwan; 4 Department of Pathology, College of Medicine, Taipei Medical University, Taipei, 110 Taiwan; 5 Department of Pathology, Tri-Service General Hospital, National Defense Medical Center, Taipei, 114 Taiwan; 6 Genomics Research Center, Academia Sinica, Taipei, 115 Taiwan

**Keywords:** Glioblastoma, Aldolase C, Serotonin, 5-Hydroxytryptamine receptors, PPAR-γ

## Abstract

Glioblastoma (GBM) is a type of brain cancer categorized as a high-grade glioma. GBM is characterized by limited treatment options, low patient survival rates, and abnormal serotonin metabolism. Previous studies have investigated the tumor suppressor function of aldolase C (ALDOC), a glycolytic enzyme in GBM. However, it is unclear how ALDOC regulates production of serotonin and its associated receptors, HTRs. In this study, we analyzed ALDOC mRNA levels and methylation status using sequencing data and in silico datasets. Furthermore, we investigated pathways, phenotypes, and drug effects using cell and mouse models. Our results suggest that loss of ALDOC function in GBM promotes tumor cell invasion and migration. We observed that hypermethylation, which results in loss of ALDOC expression, is associated with serotonin hypersecretion and the inhibition of PPAR-γ signaling. Using several omics datasets, we present evidence that ALDOC regulates serotonin levels and safeguards PPAR-γ against serotonin metabolism mediated by 5-HT, which leads to a reduction in PPAR-γ expression. PPAR-γ activation inhibits serotonin release by HTR and diminishes GBM tumor growth in our cellular and animal models. Importantly, research has demonstrated that PPAR-γ agonists prolong animal survival rates and increase the efficacy of temozolomide in an orthotopic brain model of GBM. The relationship and function of the ALDOC-PPAR-γ axis could serve as a potential prognostic indicator. Furthermore, PPAR-γ agonists offer a new treatment alternative for glioblastoma multiforme (GBM).

**Supplementary Information:**

The online version contains supplementary material available at 10.1186/s12964-024-01645-3.

## Introduction

Glioblastoma (GBM) is a World Health Organization (WHO) grade IV malignancy and one of the most aggressive glial cell tumors of the central nervous system [1]. Objective evaluations suggest that standard therapy for GBM patients, which includes tumor resection, concurrent radiotherapy, and adjuvant chemotherapy with temozolomide (TMZ), does not result in long-term survivalas the median survival time is less than two years [2]. Many common biomolecules in glioblastoma (GBM) affect patient outcomes, including isocitrate dehydrogenase 1/2 (IDH 1/2), TP53, alpha thalassemia/mental retardation syndrome X-linked (ATRX), and O6-methylguanine DNA methyltransferase (MGMT) which contain genetic alterations [3–5]. Despite significant advancements in understanding the molecular mechanisms and in devising novel therapeutic protocols, numerous patients with GBM still exhibit low survival rates. Hence, GBM is viewed as a multifactorial tumor rather than as a condition linked to a single risk factor. Currently, GBM is categorized into three molecular subtypes (proneural, classical, or mesenchymal) according to its molecular characteristics [6, 7]. These subtypes manifest distinctive gene mutations/expressions, clinical courses, and survival rates. Therefore, the identification of changes in transcription factors and expression patterns within each subtype can assist in investigating potential drug applications and signaling pathways.

Serotonin (5-HT), which regulates mood and emotions such as fear and happiness activates various serotonin receptors (5-HTR) upon release. Fourteen receptors within seven families of serotonin receptors have been defined [8]. Previous research has indicated that serotonin disrupts G-protein complex assembly, signaling cascades, and cAMP levels [8]. Various HTRs release diverse neurotransmitters, such as dopamine, norepinephrine, and serotonin [9–11]. Clinical studies have resulted in the generation of HTRs and selective serotonin reuptake inhibitors (SSRIs) as a means to hinder HTR function and inhibit serotonin reuptake [12]. Consequently, maintaining adequate levels of serotonin in the brain is vital. Previous studies have shown that serotonin can mediate a variety of events in GBM cells, including signaling pathway activity, the response to chemotherapy, and apoptosis/autophagy. [13–18]. Various 5-HTRs can increase serotonin levels and affect other signaling pathways, such as peroxisome proliferator-activated receptor gamma (PPARγ), which has been correlated with multiple brain-related diseases and conditions, such as stroke, cancer, and tranumatic brain injury [19]. PPARγ plays a regulatory role in anti-inflammatory mechanisms, oxidative stress, neuronal death, and glucose homeostasis [20]. Recent scientific studies have identified PPARγ as a therapeutic target in GBM patients [21, 22]. For example, the PPARγ agonist, pioglitazone enhances performance. Furthermore, PPARγ may also contribute to a reduction in cancer phenotypes and characteristics induced by serotonin [23]. It is currently unclear whether the function of PPARγ regulating serotonin secretion is impaired in GBM. Nonetheless, research and medical interventions for GBM utilizing 5-HTR inhibitors and SSRIs are in progress [24].

Aldolase, an enzyme that plays a critical role in metabolism and glycolysis, has three isoforms: aldolase-A (ALDOA), aldolase-B (ALDOB), and aldolase-C (ALDOC) [25]. ALDOA is widely expressed in most cancers and is associated with poor survival [26]. The roles of ALDOB or ALDOC vary across different cancer types. Several studies have suggested that ALDOB can obstruct metastasis and invasiveness of hepatocellular carcinomas [27]. ALDOC is expressed in specific regions of the brain and its expression correlates with development, injury, and trauma. Suppressed ALDOC expression was observed in GBM, and this lack of expression was significantly correlated with various clinicopathological factors [26]. However, further investigation is necessary to determine the exact mechanism of action involved. In this study, we found that hypermethylation of the ALDOC promoter suppresses its expression. This, in turn, leads to abnormal serotonin production and deactivation of the PPARγ pathway, which results in malignant GBM. The incorporation of SSRIs and PPARγ agonists into current TMZ treatment regimens may yield positive outcomes. Accordingly, the ALDOC/PPARγ axis has become a significant component of GBM research, including the exploration of novel therapies.

## Materials and methods

### Cell culture and establishment of stable clones

The CCF-STTG1 human glioblastoma cell line was cultured in RPMI 1640 medium supplemented with 10% fetal bovine serum (FBS) (Invitrogen, Carlsbad, CA, USA). The human glioblastoma cell lines T98-G, U87-MG, and SVGp12 were cultured in EMEM supplemented with 10% FBS (Invitrogen, Carlsbad, CA, USA). The human glioblastoma cell lines A172, LN-229, Hs683, and U118-MG were cultured in DMEM supplemented with 10% FBS (Invitrogen, Carlsbad, CA, USA). The SW1088 human glioblastoma cell line was cultured in L-15 medium supplemented with 10% FBS (Invitrogen, Carlsbad, CA, USA). Cells were incubated in a humidified atmosphere, -containing 5% CO_2_ with the exception of SW1088. The ALDOC sequence and pGIPZ lentiviral shRNA mir system (Thermo, Waltham, MA, USA) were utilized to establish stable cell line. ALDOC shRNA#1: 5’-GCAGCACAGTCACTCTACATT-3’ and the shRNA#2: 5’- CTCTACCAGAAAGATGATAAT-3’. The cells were infected with lentiviruses for two days after which. Puromycin (1 µg/ml, Sigma, St. Louis, MO, USA) was used to select stable clones for two weeks. The following cell lines were obtained from the ATCC cell bank: CCF-STTG1, U87-MG, T98-G, Hs683, U118-MG, A172, LN-229, SW1088, and SVGp12. The cells were all authenticated through short tandem repeat (STR) analysis, which produced profiled loci matches of more than 80%. An assay kit was used to confirm that all cell lines were mycoplasma-free for the purposes of this study.

### In vivo model

The Institutional Animal Care and Use Committee (IACUC) of Academia Sinica approved all the animal studies (#21-12-1744). All animal experiments were performed according to the National Institutes of Health (NIH) Guidelines for the Care and Use of Laboratory Animals (publication no. 85 − 23, revised 1996). Six-week-old male NOD-SCIDγ strain mice (JAXTM NOD-Cg-Prkdcscid Il2rgtm1Wjl/SzJ; NOD-SCIDγ) obtained from The Jackson Laboratory (Bar Harbor, ME, USA) and exhibited severe combined immunodeficiency (JAXTM NOD.Cg-Prkdcscid Il2rgtm1Wjl/SzJ). To evaluate the in vivo tumorigenicity of U87-MG cells 5 × 10^4^ cells were added to 3–5 µl of PBS -mixed with Matrigel (1:1 mixure) and stereotactically injected into the brains of the animal ( with the guide screw located 2.5 mm to the right and 1.5 mm above the bregma on the skull) [28]. The syringe was gradually lowered to a depth of 3 mm below the surface of the skull. After the needle entereds the brain, an electric pump was used to pass through the cells slowly at a rate of 1 µL/minute for 6–8 min to prevent any reverse flow. On the day of tumor injection, the mice were randomly assigned to groups, and various treatments were initiated: the vehicle group received PBS, while the treatment group received either a low dose (10 mg/kg) or a high dose (40 mg/kg) of GW0742 with or without TMZ (at a dosage of 20 mg/kg) via oral gavage seven times per week (*n* = 8 mice per group). We measured the volume of the tumor and the body weight on a weekly basis. The tumor volume was calculated using the following formula: tumor volume = 1/2LW^2^. When the orthotopic tumor was removed after seven weeks, the cell fluorescence/luminescence signal at the endpoint was analyzed using IVIS. The survival time of each mouse was recorded, and survival curves were plotted according to the treatment group.

### Case selection

Between 1997 and 2005, 50 patients were diagnosed with different grades of gliomas at the Tri-Service General Hospital in Taiwan. Our cohort contained 1 of grade 1_pilocytic astrocytoma, 3 of grade 2_oligodendroglioma, NOS, 1 of grade 2_astrocytoma, IDH-mutant, 1 of grade 2_glioblastoma, IDH-wildtype, 2 of grade 3_Oligodendroglioma, NOS, 2 of grade 3_Astrocytoma, IDH-mutant, 28 of grade 4_Glioblastoma, IDH-wildtype, 10 of grade 4_Diffuse midline glioma, H3 K27-altered and 2 of grade 4_Astrocytoma, IDH-mutant. A retrospective review of each patient’s medical records was used to collect clinical information and pathology data. All patients were diagnosed according to the World Health Organization (WHO) Classification of Central Nervous System Tumors (2021). Most patients had follow-up data, with the longest clinical follow-up time begin 60 months. The study at Tri-Service General Hospital (number 098-05-295) was approved by the Institutional Review Board after obtaining written informed consent from each patient who participated in the study.

### Chemicals and antibodies

Inositol (catalogue number PHR1351) was acquired from Sigma (St. Louis, MO, USA). The anti-serotonin antibody was purchased from Abcam (catalog number ab66047). RS-127,445 (item number R2533) and serotonin powder (item number H9523) were both obtained from Merck (Kenilworth, NJ, USA). GW0742 (item number S8020) and Pioglitazone (item number AD-4833) along with asenapine maleate (item number S1283) and myo-inositol (item number S4530) were purchased from Selleckchem (Houston, TX, USA). A DMSO solution was used to dissolve all the chemicals.

### Bisulfite conversion and methylation-specific PCR

Genomic DNA was isolated from GBM cells at 85% confluent using the DNeasy Blood & Tissue Kit (Qiagen, 69,504). Bisulfite conversion was performed using the EpiJET Bisulfite Conversion Kit (Thermo Scientific, #K1461) according to the manufacturer’s instructions. PCR amplification of bisulfite converted DNA was performed using Phusion U Hot Start DNA Polymerase (Thermo Scientific, F555S) with specific primers designed by- MethPrimer and MethylPrimer Express. After PCR amplification, the samples were purified, and the methylation status was assessed by visualization on a 3% agarose gel.

### Immunofluorescence microscopy

The cells were cultured in 8-well chamber slides, fixed in 4% paraformaldehyde, permeabilized, and then exposed to primary antibodies, followed by incubation with secondary FITC- or Alexa Fluor 594-conjugated anti-mouse or anti-rabbit antibodies. The slides were examined, and images were captured with a Zeiss LSM 510 META microscope (Carl Zeiss, Jena, Germany). The nuclei were stained with 4’,6-diamidino-2-phenylindole (DAPI) to aid in visualization of the cells.

### Ingenuity pathway analysis (IPA)

A 1.5-fold change was dected for shALDOC-1 and shALDOC-2 compared with vector control samples based on the expression values from microarray chips. The aforementioned values were imported into IPA for analysis of upstream regulators. According to the IPA results (Supplementary Table 5), activated upstream regulators are shown in orange, while inhibited upstream regulators are showen in blue.

### In silico analysis

Clinical information and genomic matrix files were downloaded from the Cancer Genome Atlas (TCGA) database using the UCSC cancer browser website (https://genome-cancer.ucsc.edu/proj/site/hgHeatmap/) and from the Chinese Glioma Genome Atlas (CGGA) database using the GlioVis website (https://gliobis.bioinfor.cnio.es/) by clinicians and researchers. The GEPIA website (https://gepia.cancer-pku.cn/index.html) was used to assess the expression levels of genes in the different groups. All CCLE comprehensive datasets (RNA-seq gene expression, methylation, and metabolomics data) were downloaded from the CCLE website and analyzed using Prism software. Statistical analysis was performed using SPSS 17.0 software (SPSS, Inc., Chicago, IL, USA). Statistical differences between the two groups were analyzed using either a paired t-test or a Mann-Whitney U test. p values less than 0.05-indicated statistical significance.

### RT-quantitative PCR

The cells were lysed using TRIzol reagent (Invitrogen, Carlsbad, CA, USA), and total RNA was extractedaccording to the manufacturer’s instructions. Nanodrop spectrophotometer (Thermo, Waltham, MA, USA) was used to determine the quantity of RNA. Reverse transcription-PCR (RT-PCR) was performed using a SuperScript III kit (Invitrogen, Carlsbad, CA, USA), according to manufacturer’s instructions. To obtain a standardized expression level, the expression of target genes was compared with that of ribosomal protein S26, which served as an internal control. All primers were designed by referencing PrimerBank and previous publications (refer to Supplementary Table 5). MSP and BSP primers were designed using the MethPrimer website.

### Western blot

The cells were lysed in RIPA buffer for 30 min and then centrifuged at 13,000 rpm for 15 min at 4 ^o^C. The membrane/cytoplasmic protein fractions of the cultured cells were obtained using the Mem-PER Plus Membrane Protein Extraction Kit (Thermo, Waltham, MA, USA). The protein concentration was measured using a BCA protein assay (Thermo, Waltham, MA, USA). Total proteins (30 µg) were separated by SDS-PAGE on 10% polyacrylamide gels and transferred to PVDF membranes. The membranes were hybridized with primary antibodies overnight after blocking for 30 min in 5% nonfat milk. Immunoblotting was performed with primary antibodies against DNMT1 (GeneTex, Hsinchu City, Taiwan), p-Akt (Cell Signaling Technology, Danvers, MA, USA), Akt (Cell Signaling Technology, Danvers, MA, USA), HTR2B (GeneTex, Hsinchu City, Taiwan), ALDOC (Abcam, Cambridge, UK), PPARγ (Abcam, Cambridge, UK), PTGS2 (GeneTex, Hsinchu City, Taiwan), NR2F1 (GeneTex, Hsinchu City, Taiwan) and β-actin (Sigma, St. L ouis, MO, USA). A chemiluminescence system was used to visualize the immunoreactive bands (Amersham ECL PlusTM, GE Healthcare Life Sciences, Chalfont St. Giles, UK).

### Analysis of microarray gene expression data and microarray data collection

We isolated RNA (1–2 µg) from GBM cells infected with shLuc or shALDOC lentivirus using an RNeasy Mini kit. Affymetrix GeneChip products (human genome U133A plus 2.0) were used per the GeneChip User Manual to synthesize cRNA from total RNA and hybridize and scan microarrays. We normalized the raw gene expression data and used R-project statistical software (http://www.r-project.org/) coupled with Bioconductor packages to conduct the analysis. We used the t statistic to generate a cutoff value of > 1.5 fold changeand applied this value as the threshold to determine gene candidates that were differentially expressed between the control and overexpression models after RMA normalization (Supplementary Table 2). Finally, we uploaded the list of predicted upstream regulators and canonical pathways (found using IPA to Ingenuity.

### Construction of genes and production of lentiviruses

We obtained the lentiviral envelope and the packaging plasmid (pMDG and p△8.91) from the National RNAi Core Facility (Academia Sinica, Taiwan). CLONTECH (CA, USA) provided the ALDOC lentiviral shRNA constructs and the nonsilencing pGIPZ, an shRNA construct that does not bind to target DNA. Using a calcium phosphate transfection method, lentiviruses together with pM.DG, p△8.91 and the shRNA construct were cotransfected into 293T cells. The cells were incubated for 48 h and then infected with polybrene (2 g/ml) after the lentiviruses were harvested. Puromycin (2 µg/ml) was used for one week to select cells with altered ALDOC expression. For further experiments, a useful GL reporter gene (luciferase + green fluorescent reporter gene) plasmid was also prepared to infect ALDOC two-way stable cells.

### Migration and invasion assays using in a Boyden chamber

The migration experiment was performed on polycarbonate filters (GE Healthcare Life Sciences, Chalfont St. Giles, UK) using human fibronectin (1 mg/ml) from Sigma (St. Louis, MO, USA). In each well in the lower part of the Boyden chamber, 10% FBS was added to the cell culture medium. In all, 1.5 × 10^4^ cells in serum-free culture medium were seeded into each well corresponding to the upper part of the Boyden chamber. For the invasion experiment, 10% Matrigel (BD Biosciences, San Jose, CA, USA) was applied to the other side and mixed with human fibronectin at a concentration of 1 mg/ml. The lower part of the Boyden chamber was filled with culture medium containing 10% FBS. Each well of the Boyden chamber was seeded with cells in serum-free medium. After a specified time (migration: 8 h, invasion: 14 h), the insert was removed and the cells were stained with Giemsa solution and counted under a light microscope (400x, 8 random fields per well). Three independent experimental replicates and four replicates of each sample were included.

### Analysis of glucose uptake and lactate production

A colorimetric glucose and lactate assay kit (BioVision, Milpitas, CA, USA) was used to measure glucose consumption and lactate production according to the manufacturer’s instructions. Briefly, cells (intracellular metabolites) from the specified experiments were incubated with assay buffer containing enzymes and glucose/lactate probes. Optical densities were then determined at wavelengths of 570/450 nm. Cell numbers were calculated and normalized to the background.

### Cell viability assays

A Trevigen tetrazolium salt 3-(4,5-dimethylthiazol-2-yl)-2, 5-diphenyltetrazolium bromide (MTT) cell proliferation assay kit was used to assess cell viability according to the manufacturer’s instructions (Trevigen, Gaithersburg, MD, USA). In proliferation and cytotoxicity assays, MTT is used to determine cell viability. Cells were seeded into 96-well microplates at a density of 2,000 cells/100 mL of culture medium. After seeding, the cells were treated for 24, 48–72 h with dimethyl sulfoxide (DMSO) as a control or with different doses of drugs. A microplate reader (Spectral Max250; Molecular Devices, Sunnyvale, CA, USA) was used to measure the optical density at 570 nm after the cells were incubated for 4 h in medium containing MTT and lysed with DMSO.

### Tissue microarray and immunohistochemistry

We prepared a TMA containing GBM tissue and a small amount of corresponding adjacent noncancerous brain tissue. For each patient, we selected three 1 mm cores from different areas of the tumor tissue. A pathologist evaluated the histopathological diagnosis of all samples using hematoxylin and eosin-stained slides. Serial 5-µm thick sections of tissue microarrays (TMAs) were stained using an automated immunostainer (Ventana Discovery XT autostainer, Ventana Medical Systems, Tucson, AZ, USA). Sections were first dewaxed in an oven at 60^o^, deparaffinized in xylene and rehydrated in graded alcohol solutions. Heat-induced antigen retrieval was performed using Tris-EDTA buffer (pH 8.0) for 30 min. Staining was performed with a rabbit polyclonal anti-human ALDOC antibody (1:400, Cat.T0906, Abcam (Epitomics), Cambridge, UK) and with antibodies against, PTGS2 (1:100, GTX00656, GeneTex, Hsinchu, Taiwan) and NR2F1 (1:250, GTX4801, GeneTex, Hsinchu, Taiwan).

### Interpretation of tissue microarray staining by immunohistochemistry

An independent pathologist blinded to patient outcome assessed the IHC staining. The only IHC signals detected in the cytoplasm and nuclei of tumor cells were those associated with aldolase family members. A tissue microarray was used to score the tumor for ALDOC/PTGS2 expression based on intensity scores of 0, 1 or 2. The percentage scores were calculated based on a scale of 0∼100. Finally, we used the intensity X percentage to determine the total IHC score and then used a 50% cutoff for the high- and low- expression groups. Immunoreactivity was recorded in terms of both intensity and percentage. The method for interpreting immunostaining was described in a previous study. A score of 0 was defined as the absence of cytoplasmic staining or cytoplasmic staining in less than 5% of the tumor cells. Patients with a score of two or more points were considered to have high expression. A score of 0 or 1 + represents low expression of the candidate gene and indicates loss of expression.

### Statistical analysis

The nonparametric Mann-Whitney U test was used to analyze the statistical significance of differences among three independent experiments. SPSS 17.0 software (SPSS, Chicago, IL, USA) was used for statistical analysis. A paired t-test was used to compare the levels of ALDOC/PTGS2 expression by IHC in cancer tissues with those in adjacent normal tissues. Pearson’s chi-squared test was used to identify associations between clinicopathological categorical variables and ALDOC/PTGS2 IHC expression levels. The Kaplan-Meier (KM) method was used to estimate survival rates, and the log-rank test was used for comparisons. Patients lost to follow-up were censored from the follow-up period. Multivariate and univariate analyses were performed using Cox proportional hazards regression analysis with or without adjustment for tumor stage, lymph node stage and metastasis, and ALDOC/PTGS2 expression levels. All differences were considered significant at a P value of 0.05.

## Results

### Hypermethylation and loss of ALDOC function in GBMs

**Fig. 1:**
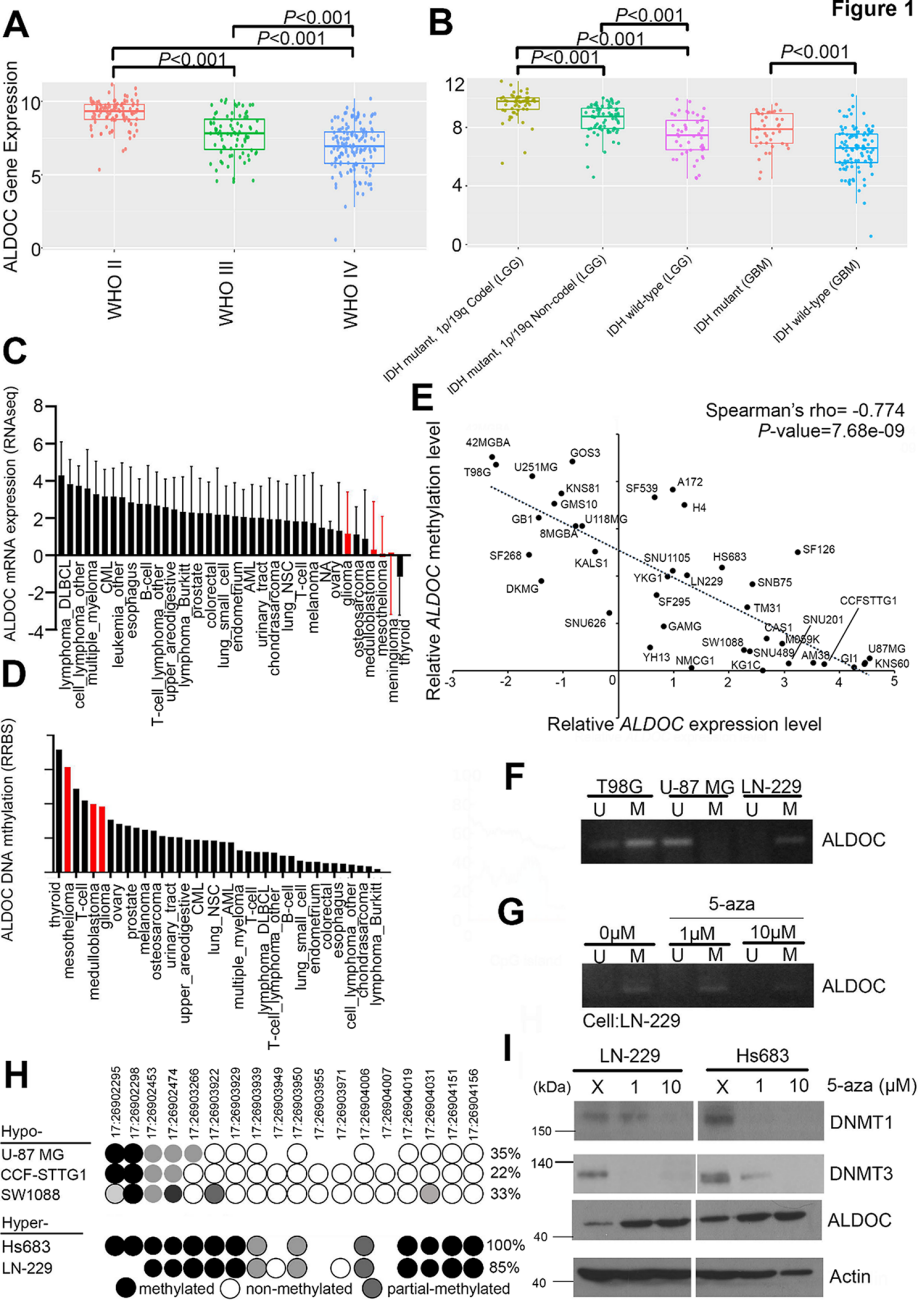
Hypermethylation and loss of ALDOC function in GBM cells. (**A**) The expression level of ALDOC according to the WHO classification of brain tumors. This database extracted ALDOC profiles from CGGA RNA-seq files. (**B**) The expression level of ALDOC in GBM patients with several genetic alterations. This database extracted ALDOC profiles from CGGA RNA-seq files. (**C**) ALDOC expression levels across multiple cancer. Red indicates glioma-related tumors. This database extracted ALDOC profiles from CCLE RNA-seq files. (**D**) ALDOC methylation level across multiple cancers from the CCLE website. Red indicates glioma-related tumors. This database extracted ALDOC profiles from CCLE DNA methylation files. (**E**) Correlation diagram showing the ALDOC methylation level of a specific fragment (17:26903951–26,904,951) and the expression level (Spearman’s rho=-0.774, *p* = 7.7e-09). This database extracted ALDOC profiles from CCLE GBM cell line files. (**F**) Characterization of the methylation status of ALDOC in various GBM cell lines (T98G, U-87MG, and LN-229) by methylation-specific PCR. (**G**) Characterization of the methylation status of ALDOC in untreated LN-229 cells and those treated with 5-Aza (1 µM and 10 µM) treatment by methylation-specific PCR. (**H**) Quantification of the percentage of CpG sites in the ALDOC promoter region (17:26903951–26,904,951) in various GBM cell lines by bisulfite-specific PCR and pyrosequencing. This database extracted ALDOC methylation status data from CCLE GBM cell line files. (**I**) DNMT1, DNMT3 and ALDOC protein levels in untreated GBM cells and those treated with 5-Aza. The data from three independent experiments are presented in **F**, **G**, and **I**

This study examined alterations in ALDOC expression in different subtypes of brain cancer. Our analysis revealed a significant decrease in ALDOC expression was found in patients with WHO stage II and III low-grade glioma (LGG) as well as WHO stage IV glioblastoma (GBM) compared with nontumor samples (Fig. 1A). Further classification revealed that ALDOC was generally less frequently expressed in GBM than in oligodendrogliomas, astrocytomas, and LGGs (Fig. S1). This article describes several common genetic alterations. The manifestations of ALDOC expression can be determined by the IDH1 mutation status or by the codeletion events of chromosome 1p/19q (Fig. 1B). The pancancer profile also demonstrated lower ALDOC expression levels in brain tumors, such as gliomas, medulloblastomas, and meningiomas, than in other cancer types (Fig. 1C). Therefore, the ALDOC expression level is closely associated with the occurrence of brain cancer.

Recent studies have indicated that the ALDOC promoter region is methylated [29]. This could affect the levels of ALDOC RNA, and therefore, it is hypothesized that the promoter region plays a crucial role in ALDOC silencing. To evaluate the degree of methylation, in silico analyses were conducted. The pancancer profile revealed increased methylation levels in certain intracranial malignancies (Fig. 1D). After the correlation between the ALDOC methylation status and RNA expression level was analyzed in GBM cell lines, a significant negative correlation was found in GBM cell lines according to the Cancer Cell Line Encyclopedia (CCLE) (Spearman’s rho = -0.774, pvalue = 7.7e-09) (Fig. 1E). To confirm these findings, three distinct GBM cell lines with varying methylation levels were selected. Our study revealed that the ALDOC gene is hypermethylated in T98G cells, while U-87 MG cells exhibit hypomethylation, and LN-229 cells exhibit an intermediate level of methylation, as detected by methylation-specific PCR (Fig. 1F). Furthermore, our findings suggested that the methylation levels in LN-229 cells decreased in a dose-dependent manner after treatment with the demethylating agent 5-Azacitidine (5-Aza) (Fig. 1G). Methylation-specific PCR (MSP) assays designed to amplify and characterize predicted methylation events. We furtehr review the assessment of methylation by bisulfite-specific PCR (BSP) amplification and sequencing. Our results indicated that the ALDOC promoter was hypermethylated in the A172 and LN-229 cell lines, while the ALDOC promoter was hypomethylated in the U-87MG, CCF-STTG1, and SW1088 cell lines (Fig. 1H). These results are consistent with the MSP analysis and CCLE profile results. Furthermore, DNMTs mainly regulate DNA methylation regulation. After 5-Aza treatment, DNMT1 and DNMT3 protein expression was reduced, while ALDOC expression was restored in GBM cells (Fig. 1I). These findings indicate that reduced ALDOC expression in GBM is due to hypermethylation.

### ALDOC triggers metabolic reprogramming in GBM cells

**Fig. 2:**
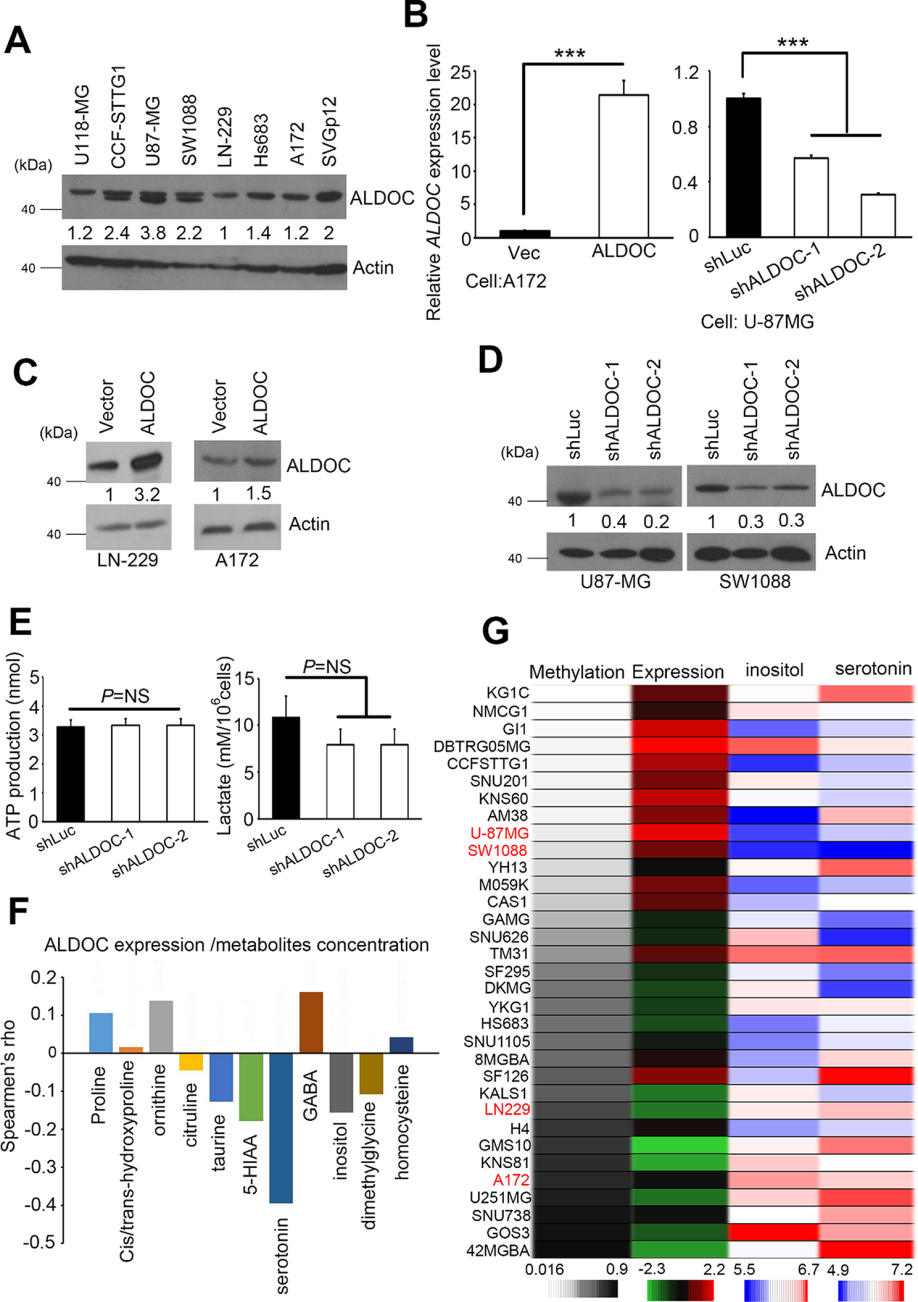
ALDOC regulates various metabolic events and metabolites in GBM. (**A**) Immunoblotting was used to determine the levels of endogenous ALDOC proteins in GBM cell panels. Actin served as the internal control. (**B**) Quantification of the expression level of *ALDOC* in ALDOC two-way (overexpressing and knockdown) stable cells by RT-qPCR. (**C**) Protein levels of ALDOC in two ALDOC-overexpressing stable cell lines, LN-229 and A172, were analyzed by immunoblotting. Actin served as the internal control. (**D**) Protein levels of ALDOC in two stable ALDOC-knockdown stable cell lines, U87-MG and SW1088, were analyzed by immunoblotting. Actin served as the internal control. (**E**) ATP concentration and lactate production in a stable ALDOC-knockdown U87-MG cell model. (**F**) Computation-dependent association analysis was performed in GBM cells using metabolite concentrations and ALDOC expression, and events with significant differences were screened. Spearman’s nonparametric method was used to determine the significance of the associations. This database extracts transcriptomic and metabolomic data from CCLE omics files. (**G**) The heatmap shows the methylation status, ALDOC expression level, and inositol/serotonin production in the GBM cell panel. This database extracted various profiles from CCLE omics files. The means and standard errors from three independent experiments are presented in B and E. The Mann-Whitney *U*-test was used to analyze the significance of the difference; *** *p* < 0.001

To assess the impact of ALDOC loss of function and to validate the in silico results, we examined the endogenous protein level of ALDOC in multiple GBM cell lines (Fig. 2A). Subsequently, we generated stable cell lines for ALDOC overexpression and knockdown using suitable cells. A172 and LN-229 cells were utilized to overexpress ALDOC, while U87-MG and SW1088 cells were used for ALDOC knockdown (Fig. 2B and D). Since ALDOC is involved in glycolysis, we collected equal amounts of cells from each group of ALDOC knockdown cells to perform ELISA for the measurement of glucose, lactate, and ATP levels. Notably, our results demonstrated no significant differences in lactate and ATP production or glucose utilization rates compared with those of the controls (Fig. 2E and S2A). Likewise, no important changes in metabolic activity were detected in the group treated with 5-Aza (Fig. S2B).

The CCLE metabolomics platform was used to investigate further metabolites linked to ALDOC expression [30]. This particular dataset contains measurements of several metabolites across multiple cell lines. Each metabolite can be produced and evaluated in relation to past events, such as methylation and expression. Earlier studies have shown connections between diverse events, such as methylation and expression levels of specific genes, with carbohydrates, amino acids, and lipids [31]. In this study, we investigated the correlation between ALDOC expression events and cellular metabolite concentrations to identify potential dependencies. Our results revealed a negative correlation between ALDOC expression and the serotonin metabolite 5-hydroxyindoleacetic acid (5-HIAA) (Fig. 2F). In contrast, ALDOC methylation events were negatively correlated with ALDOC expression and were positively correlated with the concentrations of the aforementioned metabolites (Fig. S3). Changes in ALDOC methylation and expression were observed in GBM cell lines. The metabolites myo-inositol and serotonin underwent significant changes (Fig. 2G). These findings suggest that GBM cells undergo metabolic reprogramming due to ALDOC loss of function or methylation.

### Dysfunction of ALDOC in GBM results in serotonin production and pathway activation

**Fig. 3:**
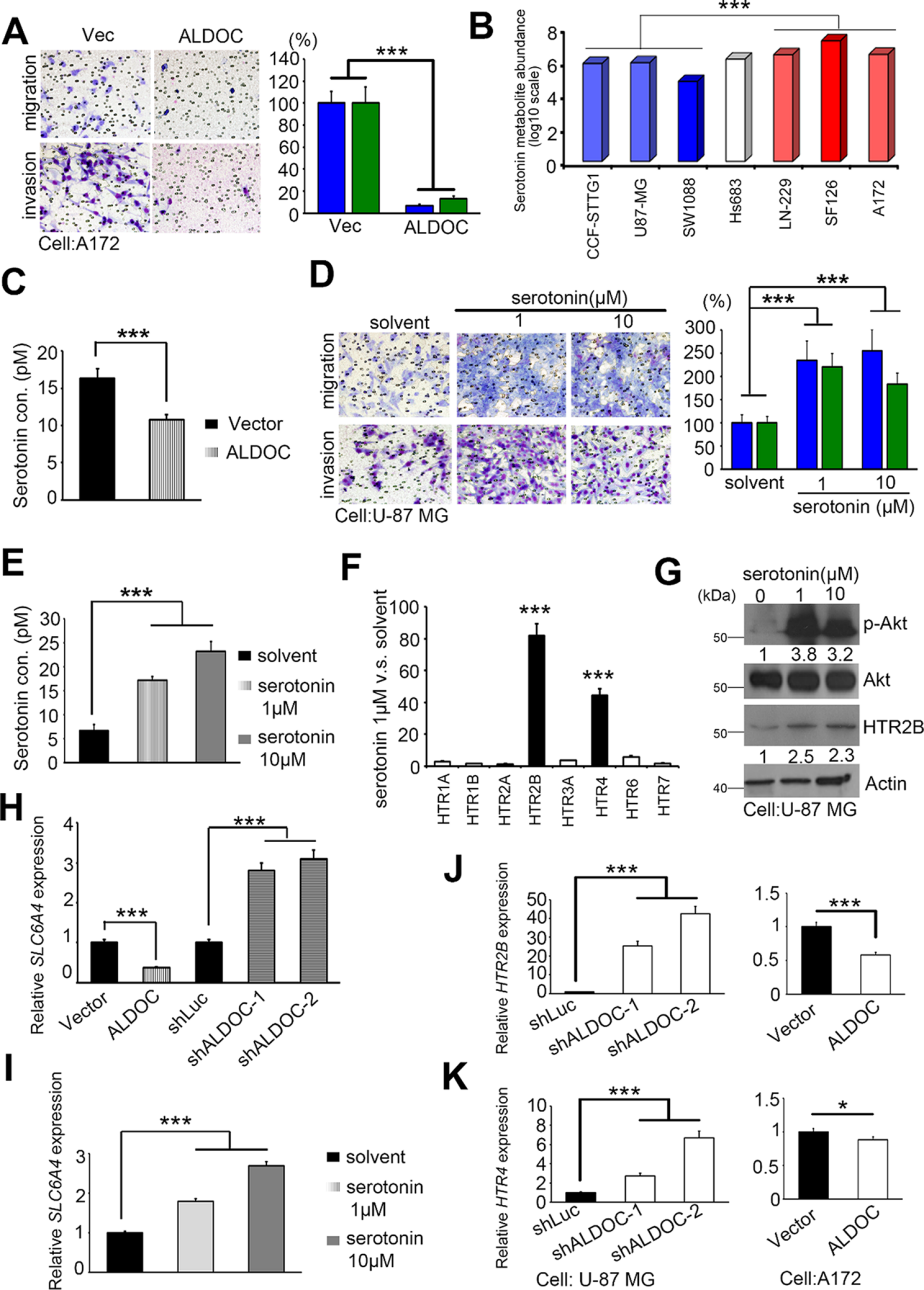
ALDOC regulates migration/invasion capabilities and the response to serotonin in GBM. (**A**) Migration/invasion ability of A172 cells expressing the exogenous vector or overexpressing ALDOC. Scale bar: 100 µM. (**B**) Bar graphs showing serotonin levels in GBM cell lines from CCLE metabolite profiles. We downloaded the results from the CCLE metabolomics pattern and customized low and high serotonin production in GBM cell lines. (**C**) Serotonin concentration of A172 cells expressing the exogenous vector or overexpressing ALDOC. (**D**) After exposure to serotonin, U-87MG cells were subjected to Giemsa staining to evaluate their migration ability (1 µM and 10 µM) treatment. Scale bar: 100 µM. (**E**) Serotonin concentrations in untreted U-87MG cells and thoese treated with serotonin (1 µM and 10 µM). (**F**) The expression level of *HTR* members in the serotonin treatment group. (**G**) The levels of p-Akt, Akt, and HTR2B in U-87MG cells treated with serotonin were determined by Western blot analysis to change in a dose-dependent manner. (**H**) The expression level of *SLC6A4* in the ALDOC-knockdown and overexpression models. (**I**) The expression level of *SLC6A4* in the serotonin treatment group. (**J**) Quantification of *HTR2B* expression levels in ALDOC two-way (overexpressing and knockdown) stable cells by q-PCR. (K) Quantitative analysis of the expression of HTR4 in ALDOC two-way cells (overexpressing and knockdown). In A, B, C, D, E, F, G, H, J and K, the means ± SEM of three independent experiments are presented. A nonparametric Mann-Whitney *U*-test was used to determine the significance of the differences. The blue column in A represents cellular migration, while the green column represents invasion ability. * *p* < 0.05; ** *p* < 0.01; *** *p* < 0.001

To examine the impact of ALDOC and the aforementioned metabolites, we assessed various cancer characteristics using cell models in which ALDOC was either overexpressed or suppressed. We observed significant disparities in migration/invasion, but not in proliferation (Fig S4A). ALDOC expression repressed invasion compared with control cells, whereas reduced ALDOC levels were linked to elevated migration/invasion abilities (Fig. 3A and S4B-C).

Previous metabolite profiles were analyzed to investigate the correlation between these changes and serotonin alterations. Our findings indicate that the CCF-STTG1, U87-MG, and SW1088 cell lines exhibited reduced serotonin production (< 5, serotonin metabolite abundance, log10 scle), whereas the Hs683 cell line displayed moderate serotonin production, and the LN-229, SF126, and A172 cell lines exhibited increased serotonin production (> 5, serotonin metabolite abundance, log10 scle) (Figs. 2G and 3B). The concentration of measured serotonin was significantly reduced in the ALDOC overexpressing group (Fig. 3C). The addition of serotonin to the cell cultures at low concentrations increased the invasiveness and proliferation of GBM cells (Fig. 3D and E and S5). We also used fluorescent labeling of serotonin in cell models [32]. A significant increase in the signal in and around the nucleus was observed with the addition of serotonin. ALDOC inhibition resulted in an even greater increase in serotonin expression (Fig. S6). We further examined the prospective functions of serotonin in GBM models, but the introduction of serotonin alone did not influence the response of GBM cell lines to TMZ (Fig. S7). Additionally, *myo*-inositol was formerly recognized as a possible contender, but this compound did not have any noteworthy impact on the GBM cell phenotype (Fig. S8).

To confirm the role of serotonin in transsynaptic signaling through the HTR, we conducted qRT-PCR screening of all 5-HTR members, including those utilized by other neurotransmitters such as dopamine and epinephrine. Our findings revealed that serotonin application led to intensified activation of HTR2B and HTR4 expression levels (Fig. 3F). We additionally explored conventional neurotransmitter signaling pathways and observed that serotonin treatment increased Akt phosphorylation (Fig. 3G). Furthermore, we have identified the serotonin-specific transporter, SLC6A4, whose expression is in sync with serotonin concentration (Fig. 3H and I). Our research using the ALDOC biphasic cell model confirmed the regulation of HTR2B and HTR4 expression levels by ALDOC (Fig. 3J and K). Our findings demonstrate that ALDOC hypermethylation or dysfunction promotes GBM cell migration and invasion via serotonin and its receptors.

### Loss of ALDOC function in GBM also affects PPARγ signaling

**Fig. 4:**
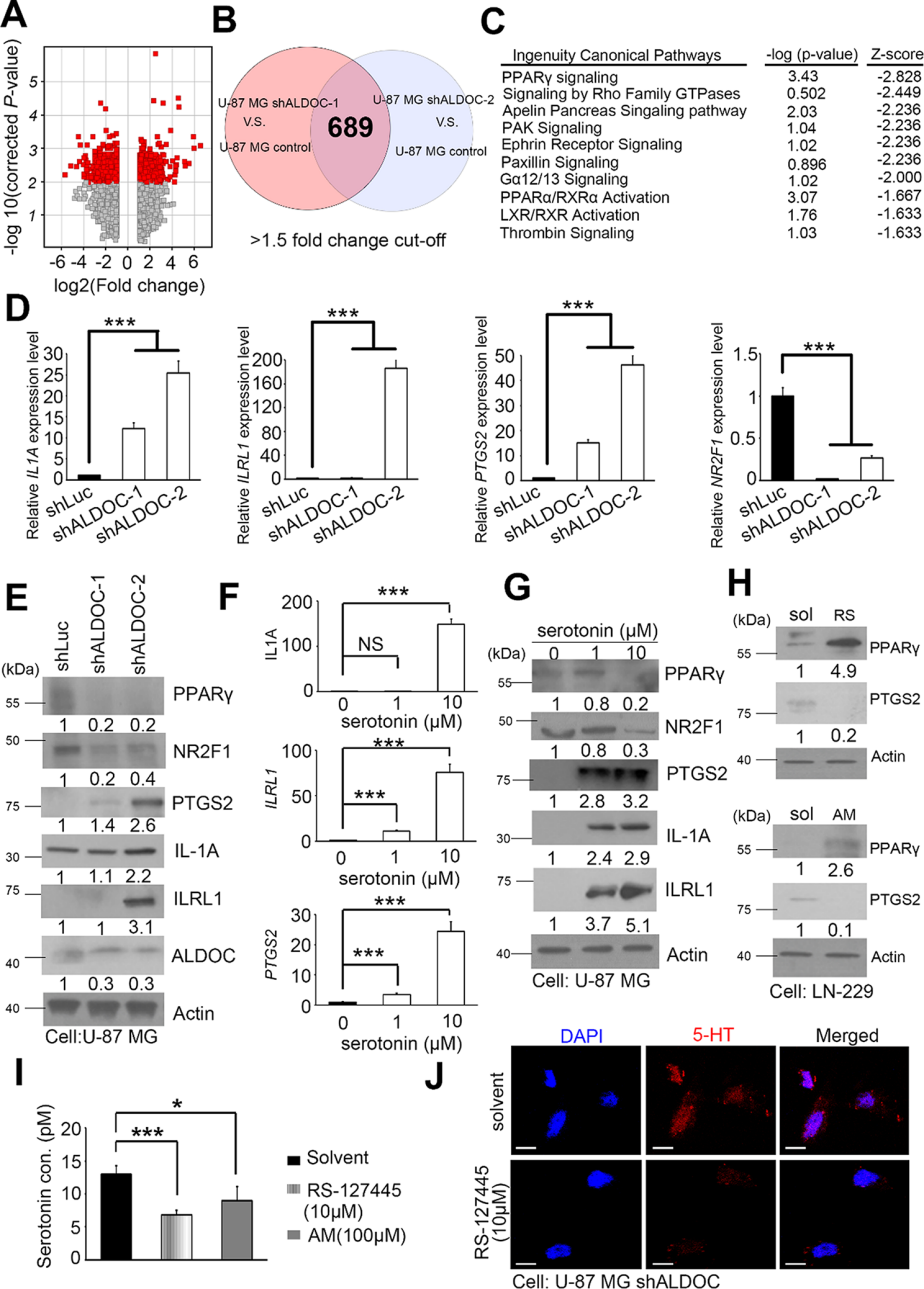
A reduction in ALDOC function is expected to decrease PPARγ signaling and its downstream targets. (**A**) The volcano plot reveals the candidate selection criteria for shALDOC versus the vector control in U-87MG cells. (**B**) The Venn diagram shows the common signatures (689 probes) between shALDOC-1 vs. control and shALDOC-2 vs. control for further interpretations. (**C**) The highest-ranking potential regulatory pathways from the common signature of shALDOC were predicted by IPA. (**D**) Quantification of the expression levels of PPARγ downstream targets (*IL1A*, *ILRL1*, *NR2F1*, and *PTGS2*) in stable ALDOC-knockdown cells by q-PCR. (**E**) The protein levels of ALDOC, PPARγ, and its downstream targets (NR2F1, PTGS2, IL-1 A and ILRL1) in stable ALDOC-knockdown cells were detected by immunoblotting. Actin served as the internal control. (**F**) Quantitative PCR was used to quantify the dose-dependent changes in the expression levels of PPARγ downstream targets (*IL1A, ILRL1* and *PTGS2*) in U-87MG cells. (**G**) The protein levels of PPARγ and its downstream targets (NR2F1, PTGS2, IL-1 A and ILRL1) in U-87MG cells increased in a serotonin dose-dependent manner. Actin served as an internal control purposes. (**H**) The protein levels of PPARγ and its downstream target PTGS2 in LN-229 cells treated with or without 5-HT receptor inhibitors (RS-127,445 and AM) were detected by immunoblotting. Actin served as an internal control. (**I**) Serotonin concentration in U-87MG shALDOC cells treated with 5-HT receptor inhibitors (RS-127,445, 10 µM and AM, 100 µM) treatment. (**J**) Immunofluorescence assay of U-87MG shALDOC cells after RS-127,445 treatment. Red: serotonin; Blue: DAPI. Scale bar: 20 µM. AM: Asenapine maleate. In D, F, and I, the means ± standard error of the means are presented for three independent experiments. A nonparametric Mann-Whitney *U*-test was used to determine the significance of the difference; * *p* < 0.05; ** *p* < 0.01; *** *p* < 0.001

Serotonin is secreted and transmitted via 5-HT receptors to regulate downstream factors. An ALDOC-knockdown model with two independent clones was used in the GBM study to establish transcriptome profiles and to identify the primary pathway of influence. Probes were normalized and a cutoff value of > 1.5 fold change was set for further prediction (Fig. 4A). The study revealed that 689 probes overlapped in two independent shALDOC versus control events (Fig. 4B). IPA predicted that the inhibition of ALDOC would also inhibit the PPARγ signaling pathway (Fig. 4C). Studies have suggested that the expression of genes downstream of PPARγ, such as IL1A, ILRL1, NR2F1, and PTGS2 [23, 33], may be influenced by serotonin metabolism. Thus, the expression levels of these genes were analyzed. The results of our study indicate that ALDOC-knockdown resulted in alterations in downstream factors that corresponded with the previous transcriptomic profile (Fig. 4D and Table S1). Conversely, the overexpression model exhibited an inverse trend (Fig. S9). Additionally, the inhibition of ALDOC suppressed PPARγ expression and the expression of downstream candidate factors (Fig. 4E). Among these factors, our focus was on NR2F1 and PTGS2. Our research using the ALDOC expression model highlights the importance of regulating the PPARγ-NR2F1/PTGS2 pathway.

We again used a cell model with added serotonin and observed a dose-dependent decrease in PPARγ expression in the GBM cell model following serotonin treatment. Furthermore, the downstream genes IL1A, ILRL1, and PTGS2 were upregulated, while NR2F1 protein activation was reversed (Fig. 4F-G). Upon administration of the HTR inhibitors/antagonists RS-127,445 and asenapine maleate (AM), PPARγ was reactivated (Fig. 4H). Treatment with these inhibitors significantly suppressed GBM cell migration, serotonin concentration, and fluorescence signals (Fig. 4I and J). We then performed a rescue experiment to investigate whether the addition of serotonin could reactivate the HTRs, potentially reducing the effect of the antagonists (Fig. S10). Our findings indicate that serotonin disrupts and inhibits PPARγ signaling in GBM and that this disruption is regulated by ALDOC.

### Modulation of the ALDOC-PPARγ axis can reduce in situ brain tumorigenicity and prolong survival

**Fig. 5:**
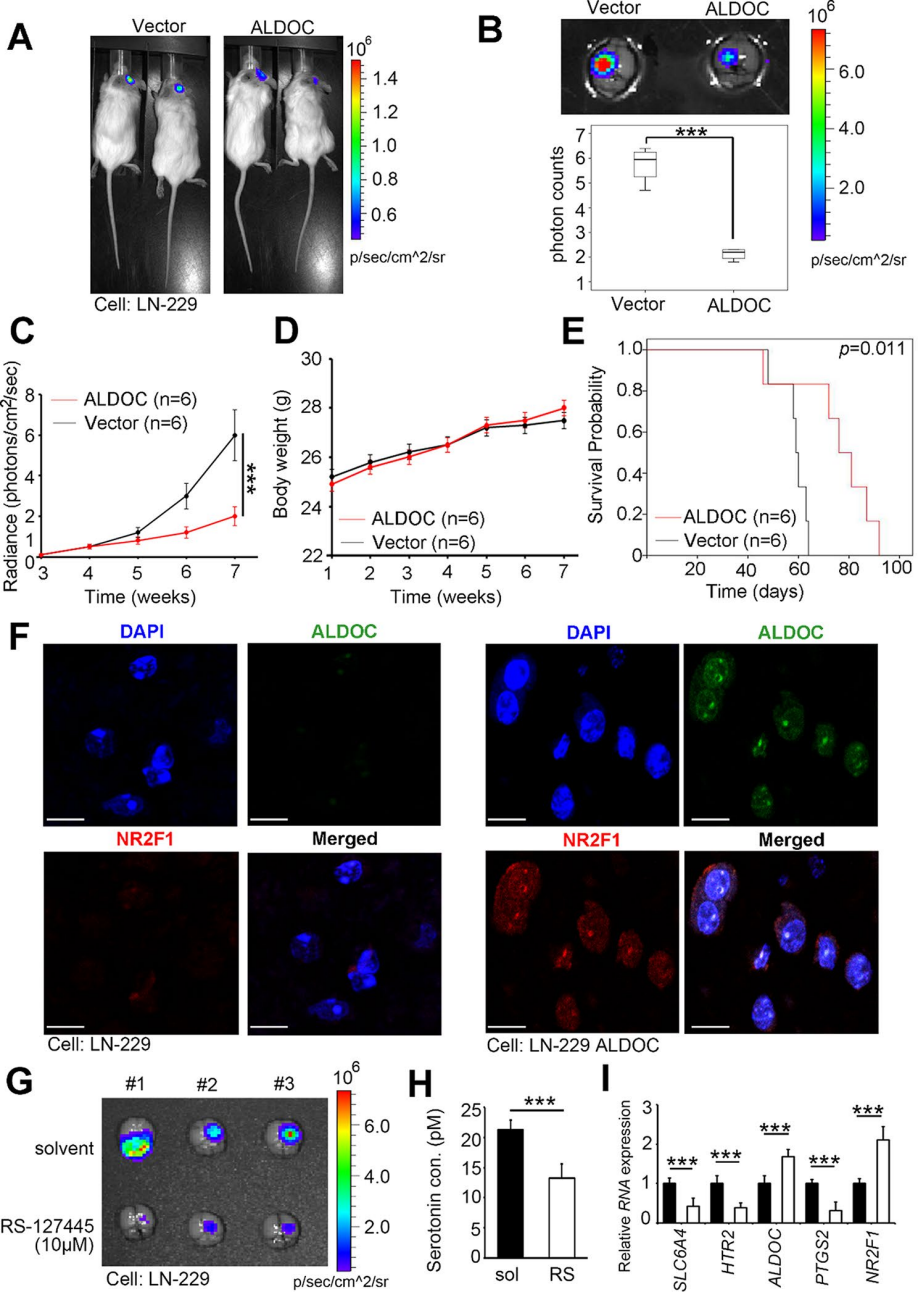
ALDOC modulates orthotopic tumor growth in GBM animal models. (**A**) An overview of an intracranial LN-229 cell injection at the first IVIS tracking signal (2nd week) between the vector group and the ALDOC overexpressing group. (**B**) Overview of the intracranial model at the endpoint in the vector and ALDOC overexpressing groups after whole-brain extraction. Quantification of the whole brain. (**C**) Continuous radiance quantification of the intracranial LN-229 cell model in the vector and ALDOC overexpression groups. (**D**) Continuous body weight quantification in the vector and ALDOC overexpression groups. (**E**) To establish an orthotopic brain model, six-week-old NOD/SCID gamma mice were injected intracranially with LN-229 cells expressing the vector control or ALDOC-overexpressing LN-229 cells. Kaplan-Meier plots of the time to death, are presented for the vector-treated or ALDOC-overexpressing mice. *n* = 6 per group, *p*-value = 0.011. (**F**) After whole-brain extraction, representative multiplex IF for several candidate proteins in the LN-229 and LN-229 shALDOC intracranial models was performed. Red: NR2F1; Green: ALDOC; Blue: DAPI. Scale bar: 150 µM. (**G**) IVIS luminescence imaging system detection in the solvent group and the 10 µM RS-127,445 group after whole-brain extraction in the LN-229 intracranial model. (**H**) Serotonin concentration in the solvent group or the 10 µM RS-127,445 group. (**I**) Quantitative PCR was used to quantify the expression levels of targets (*SLC6A4, HTR2, ALDOC, PTGS2* and *NR2F1*) in LN-229 cells after RS-127,445 treatment. A non-parametric Mann-Whitney *U*-test was used to determine the significance of the difference. *** *p* < 0.001

To evaluate the medical significance of ALDOC in animal models of GBM, we conducted in vivo tumorigenicity studies. We intracranially injected LN-229 cells and ALDOC-overexpressing cells into the mice. These cells were equipped with dual reporter genes (green fluorescent/luciferase) to ensure that the conditions were consistent among all groups during the study. Ultimately, we measured the photon counts of all groups using an in vivo imaging system (IVIS) at the endpoint. Compared with that in the vector group, the luminescence signal in the ALDOC-overexpressing group was decreased (Fig. 5A). Moreover, the ALDOC overexpressing group had a lower photon count than the whole-brain extraction group (Fig. 5B). Weekly real-time monitoring indicated that ALDOC reduced the in situ growth capability of GBM without affecting body weight (Fig. 5C and D). Compared with the control group, the group that received cells that overexpressed ALDOC had longer survival times (Fig. 5E).

To investigate particular regions of target proteins, we divided the whole brain and performed multiplex immunohistochemistry (IHC). The results indicated that in the animal experiments, the expression of ALDOC and the PPARγ downstream factor NR2F1 was significantly higher in the ALDOC overexpressing group than in the vector control group (Fig. 5F& S11). Additionally, we incorporated RS-127,445 into the above orthotopic brain tumor model. Additionally, the results indicated a significant decrease in serotonin (Fig. 5H) and related molecules, accompanied by the restoration of ALDOC and NR2F1 expression (Fig. 5I), in addition to the inhibition of tumor growth (Fig. 5G). These findings highlight the importance of both the ALDOC and PPARγ pathways in an in vivo GBM model.

### PPARγ agonists have therapeutic potential in GBM models

**Fig. 6:**
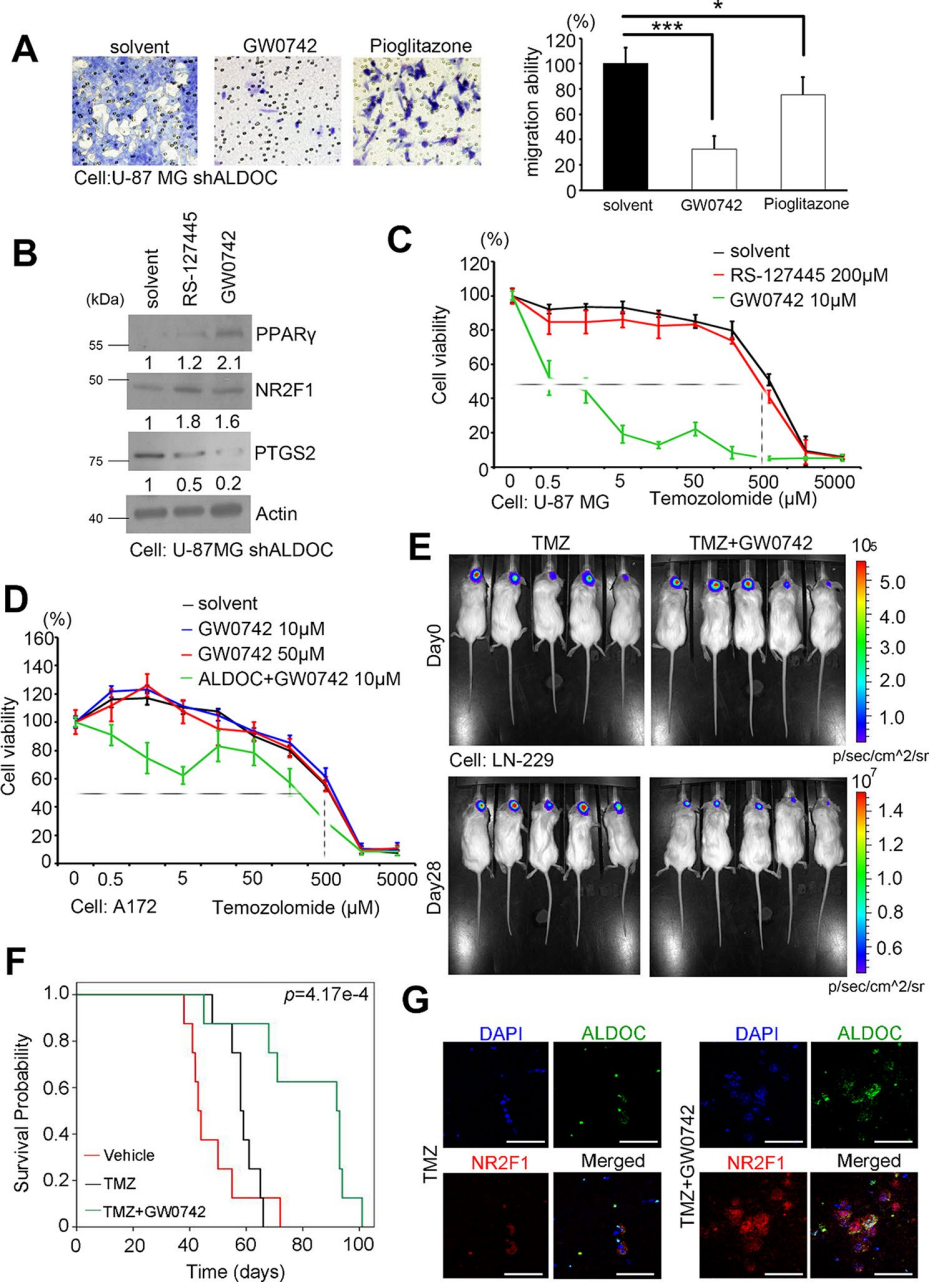
PPARγ agonists can reverse the phenotype caused by ALDOC loss in vitro and in vivo. (**A**) The ability of U-87MG shALDOC cells to migrate with or without PPARγ agonists was assessed by Giemsa staining (GW0742 and pioglitazone). Scale bar: 100 µM. (**B**) The protein levels of PPARγ and its downstream targets (NR2F1 and PTGS2) in U-87MG ALDOC-knockdown stable cells were detected by Western blotting, with or without RS-127,445/GW0742 treatment. Actin was used as an internal control. (**C**) Alamar blue assay was used to measure cell viability in U-87MG cells treated with TMZ in a dose-dependent manner and treated with GW0742 or RS-127,445. (**D**) Alamar blue assay was used to measure cell viability in an A172 TMZ dose-dependent manner with GW0742 or ALDOC overexpression combined with GW0742. (**E**) An IVIS imaging system detected the TMZ alone group or the TMZ combined with GW0742 group in the U-87MG shALDOC intracranial model. (**F**) Kaplan-Meier plots showing the survival time of each group after the U-87 shALDOC cell line was used to restablish an orthotopic brain model and after treatment with TMZ or TMZ combined with GW0742. *n* = 8 for each group, *p*-value = 4.17e-4. (**G**) Representative multiplex IHC for several candidate proteins in the intracranial LN-229 cell model treated with TMZ alone or in combination with GW0742. Red: NR2F1; Green: ALDOC; Blue: DAPI. Scale bar: 150 µM. In A and C, the means ± standard errors of the means are presented for three independent experiments. A nonparametric Mann-Whitney U-test was used to determine the significance of the differences. * *p* < 0.05; ** *p* < 0.01; *** *p* < 0.001

Although initial results suggest the clinical potential of HTR antagonists and SSRIs, these medications have limitations and may cause side effects. Therefore, we propose that enhancing PPARγ signaling could be an alternative therapeutic strategy. We treated ALDOC-knockdown cells with the PPARγ agonists GW0742 and pioglitazone [18], which resulted in a significant decrease in migration ability compared with that of the solvent control (Fig. 6A). Moreover, the group treated with the PPARγ agonists, particularly GW0742, exhibited decreased serotonin production at doses that did not impact cytotoxicity (Fig. S12). Furthermore, in the shALDOC model cotreated with serotonin and GW0742, the inhibition of PPARγ by serotonin decreased the efficacy of GW0742 (Fig. S13). To investigate the interplay of HTR antagonists as described earlier, we utilized RS-127,445 and GW0742 in a cell model in which ALDOC was knocked down. Western blot analyses demonstrated that agonists restored the expression of PPARγ and its downstream components (Fig. 6B & S14). These results suggest that PPARγ agonists are markedly more effective than HTR antagonists.

TMZ, a typical treatment option, was used to evaluate whether HTR antagonists or PPARγ antagonists were more effective. Our results revealed a negative correlation between the IC50 of TMZ and ALDOC/PPARγ expression (Fig. S4. This is in agreement with our actual experimental results. In a cell line that highly expresses ALDOC (U-87 MG), RS-127,445 alone did not enhance the effects of TMZ treatment, but the addition of GW0742 exerted significant effects (Fig. 6C). However, in cells with low ALDOC expression (A172), the overexpression of ALDOC was significant as was the treatment with GW0742 (Fig. 6D). In the animal model, after 28 days of treatment with TMZ combined with GW0742, the size of in situ tumors in the combined treatment group was significantly reduced compared with that in the group that was treated with TMZ alone (Fig. 6E). More importantly, we observed a doubling of survival time in the current animal model (Fig. 6F). In addition, the TMZ + GW0742 group demonstrated that ALDOC and NR2F1 actually restored potency (Fig. 6G). This finding suggests that PPARγ agonists in combination with TMZ may be a viable treatment option for GBM and that the expression of ALDOC should be carefully evaluated.

### The ALDOC-PPARγ axis can serve as a prognostic factor for patients with GBM

**Fig. 7:**
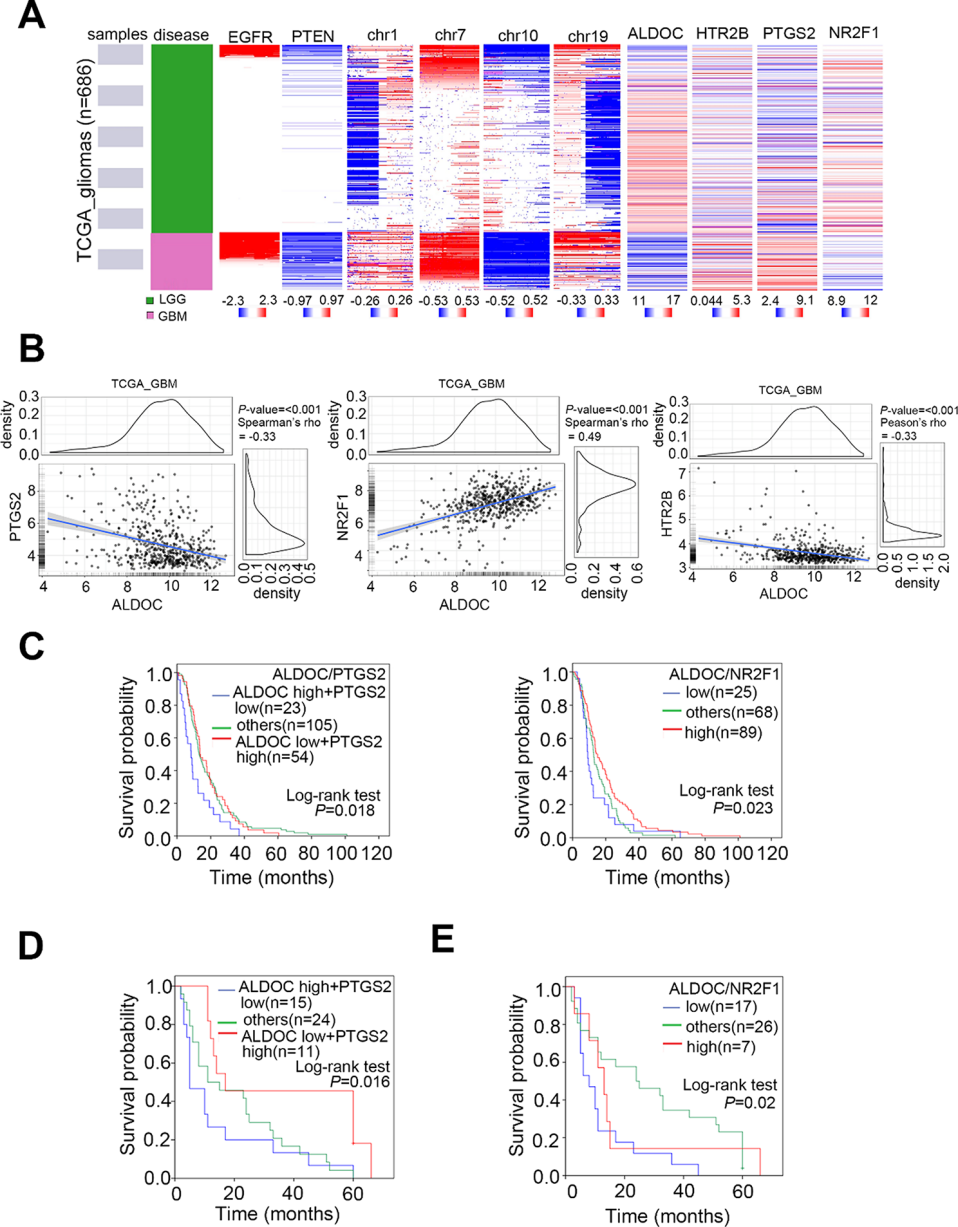
The ALDOC-PPARγ axis may have prodgnostic value in gliomas and GBMs. (**A**) Heatmap of the mRNA expression of candidates and various clinicopathological factors in TCGA glioma patients. (**B**) Correlation between *ALDOC* expression and *PTGS2/NR2F1/HTR2B* expression in the TCGA GBM cohort. A nonparametric Spearman correlation analysis was used to evaluate the significance of the correlation. (**C**) Kaplan-Meier (KM) analysis of the overall survival in patients with GBM according to *ALDOC* combined with *PTGS2* or *NR2F1* expression under various conditions. (**D**) KM analysis of the overall survival rate of patients accordin to ALDOC expression and the combined expression of the PTGS2 protein in three groups of GBM patients (ALDOC high/PTGS2 low, ALDOC low/PTGS2 high, and others) from the GBM TMA cohort. (**E**) KM analysis of overall survival in patients from the GBM TMA cohort according to ALDOC and NR2F1 protein expression at common low (score 0,1) and common high (score 2, 3) levels. The significance of the data was calculated using the log-rank test

To examine the role of ALDOC or PPARγ signaling in GBM clinical cohorts, we examined additional clinical events recorded in the TCGA glioma dataset. To examine the role of ALDOC or PPARγ signaling in GBM clinical cohorts, we assessed additional clinical data from the TCGA glioma dataset. As previously confirmed, PPARγ triggers NR2F1 and suppresses PTGS2 (Fig. 4D and E). We investigated the potential roles of ALDOC, HTR2B, PTGS2, and NR2F1 along with various clinicopathological factors of GBM, including EGFR amplification, PTEN deletion, and chromosomal abnormalities (including codeletion of 1p/19q, gain of chromosome 7, and loss of chromosome 10). These factors were used to divided patients into LGG and GBM groups based on the expression levels of our candidates, which varied by classification (Fig. 7A). The heatmap indicated that the expression of HTR2B did not differ significantly from that of the other candidates. In contrast, ALDOC showed a negative correlation with PTGS2 and a positive correlation with NR2F1. Focusing the GBM type, ALDOC was found to be associated with PTGS2 and NR2F1 in both the TCGA (Fig. 7B) and CGGA (Fig. S16) cohorts. This study reports on new prognostic markers related to ALDOC. Although prior research has highlighted its importance [34], we evaluated ALDOC in conjunction with PTGS2 or NR2F1 and found that the combinations had significant prognostic value at the RNA level (Fig. 7C). Our tissue microarray results,, obtained via immunohistochemistry demonstrated that ALDOC-PTGS2/NR2F1 protein levels predict poor survival and are correlated with tumor grade (Supplementary Tables 2 and 3). This trend was consistent with that observed for RNA and several other clinical cohorts (Fig. 7D and E). We have also provided Supplementary Tables 4 to further illustrate the potential functions, phenotypes, and pathways associated with ALDOC. In addition, we presented combination treatments with high translational medicinal value. These strategies can serve as guidelines for the treatment of GBM using precision medicine.

**Fig. 8:**
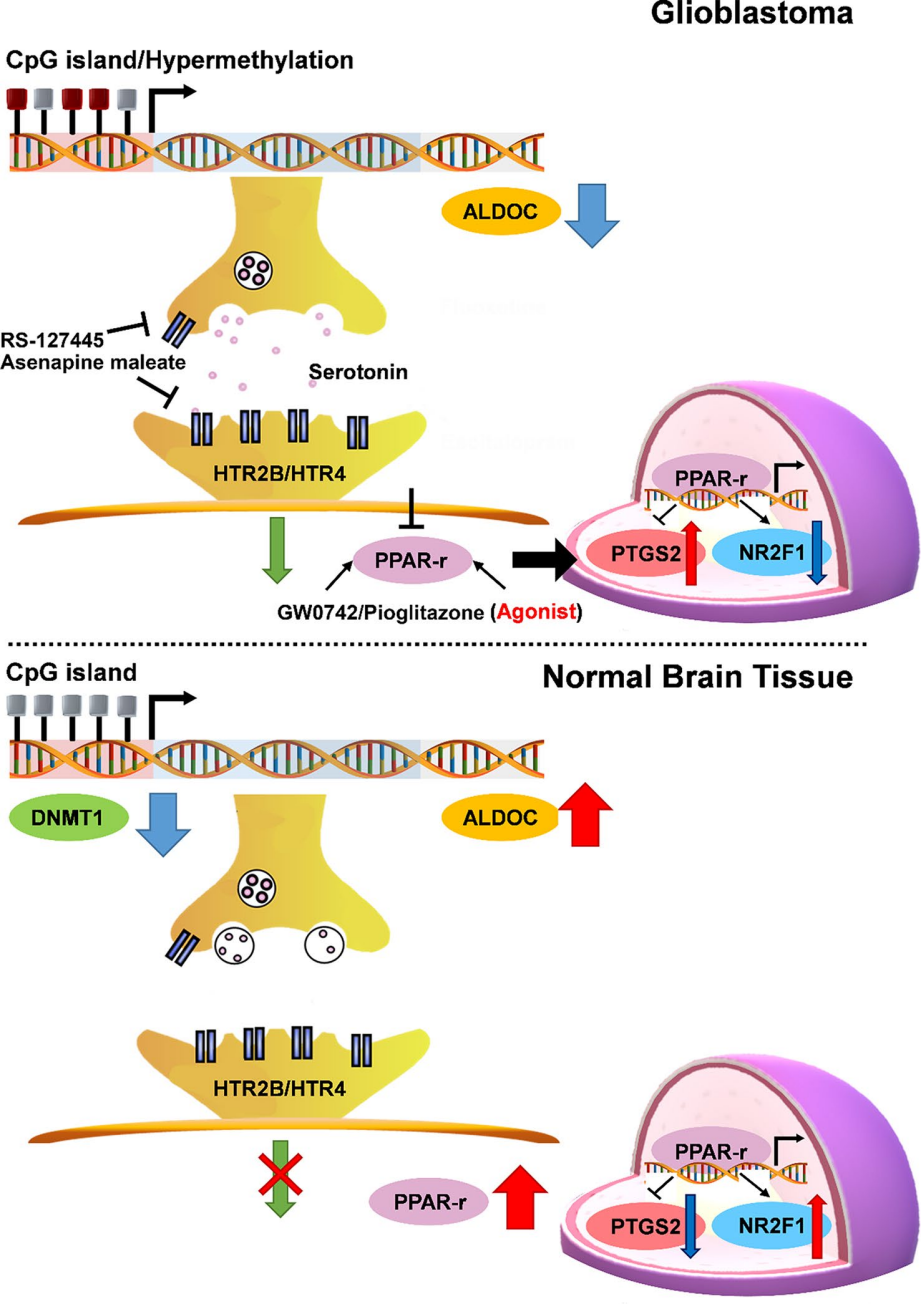
Schematic model of the relationship between ALDOC, serotonin and PPARγ signaling in GBM

## Discussion

In this study, we investigated the function of ALDOC in GBM. GBM cell lines were selected by integrating transcriptomic and metabolomic profiles to better predict outcomes. Our results revealed a negative correlation between ALDOC expression levels and hypermethylation status. In addition, we observed a positive association between hypermethylation and increased production of inositol and serotonin. Our model showed that several serotonin (5-HT) receptors were activated, which could promote GBM cell metastasis by increasing serotonin signaling. We used 5-HT antagonists to inhibit signaling and reverse the phenotype, which demonstrates their potential as inhibitors of GBM tumorigenesis. However, serotonin plays a critical role in human physiology and psychology, and the use of 5-HT antagonists can lead to anxiety, depression and other severe side effects. Therefore, we investigated the potential of PPARγ antagonists as alternative therapeutic agents. However, upon evaluation, no significant improvements in cell viability or toxicity were observed, and the antagonists had no effect on body weight in the mouse model. Although previous research has suggested an association between PPARγ antagonists and GBM [35, 36], it remains unclear how these antagonists are related to serotonin and ALDOC loss-of-function/hypermethylation events. It is important to note that these options complement the current TMZ treatment and may offer a new combination therapy.

Interestingly, selective serotonin reuptake inhibitors (SSRIs) inhibit serotonin reuptake and increase its concentration in specific brain regions [37]. Several clinical antidepressants, including escitalopram and fluoxetine, possess this function and are subject to safety regulations. Although their effects may be similar to those of 5-HT inhibitors, antidepressants are associated with significant adverse effects and are clinically restrictive [38]. In addition, it is necessary to determine whether serotonin production is related to specific 5-HT receptors and investigate the efficiency of reuptake in tissues compared with typical levels.

By combining prior observations with computational analysis, researchers have found that ALDOC expression relies on the apparent modifications in the IDH1 genetic background [26]. Both low-grade gliomas and GBM, with wild-type and mutated IDH1, showed correlations with various clinicopathological events, and the difference in ALDOC expression was statistically significant on its own. The exact molecular relationship between IDH1 and ALDOC, however, remains uncertain. The affects of the IDH1 gene on hypermethylation of the promoter region of ALDOC or on upstream transcription factor activity may significantly affect ALDOC silencing. Furthermore, due to its pivotal role in the aldolase family, ALDOC is important in connecting glycolysis and the tricarboxylic acid cycle (TCA). Therefore, it is crucial to investigate is the occurrence of a sequence of metabolic reprogramming events and whether GBM tumorigenesis results from 2-HG [39]. To address these uncertainties, we plan to validate our research findings using IDH1 knockout cell lines or by generating IDH1 R132 mutant cell lines. Our hypothesis is that ALDOC expression induction could serve as an independent factor or as part of a “two-hit” model in combination with IDH1 mutation. This discovery has the potential to advance the use of ALDOC in predicting and diagnosing GBM and other gliomas.

Several datasets focused on the omics of various cancer cell lines have been established. Technical term abbreviations such as omics will be explained when first used. The Cancer Cell Line Encyclopedia (CCLE) project offers well-organized collections of genomic, transcriptomic, proteomic, and metabolomic datasets [30, 40, 41]. In this study, we obtained GBM cell lines and their corresponding bioinformatics backgrounds from the CCLE dataset. Our analysis revealed that serotonin and inositol levels had a considerable effects on the expression of ALDOC and its methylation status. Inositol, also known as vitamin B8 [42], is an essential vitamin B complex. Scyllo-muco, D-chiro, and neo-inositol are some of the different isomers produced, and they are classified based on their structure [43]. The most common form is myo-inositol, which is synthesized from glucose 6-phosphate (G6P). Inositol-3-phosphate synthase converts G6P into myo-inositol-1-phosphate, which is then dephosphorylated by inositol monophosphatase to produce the metabolite myo-inositol [44]. Previous research has demonstrated that inositol is present in certain brain-related disorders [45, 46]. To determine the levels of myo-inositol and glutamine or the inositol/creatine ratio in GBM, magnetic resonance spectroscopy is used [47, 48]. Additionally, myo-inositol can serve as a biomarker for evaluating the effects of recurrent GBM with or without bevacizumab treatment [49]. This study contrasts the regulatory impact and metastatic capacity of inositol and serotonin on GBM cells. Furthermore, we supplemented these GBM cell lines with up to 1 mM of myo-inositol. However, our investigation did not reveal any significant changes in metastatic capacity. Nevertheless, the importance of inositol in relation to brain tumors and metabolic processes has been highlighted [50, 51]. Additionally, one hypothesis is that ALDOC regulates inositol, which requires further examination and analysis.

This study revealed that a reduction in ALDOC expression and excessive serotonin production lead to GBM phenotypes, such as metastasis, resistance to TMZ and hindered PPAR-γ signaling. The ALDOC/PPAR-γ axis serves as an autonomous prognostic marker. Both in vitro and in vivo experimental results highlight the ability of PPAR-γ agonists to restore the expression of genes associated with these phenotypes and to enhance the clinical impact of TMZ.

## STAR★Methods

**Table 1:** Key resources table

Reagent or resource	Source	Identifier
**Antibodies**		
p-Akt	Cell signaling	#9271
Akt	Cell signaling	#4685
DNMT1	GeneTex	GTX116011
ALDOC	Abcam	T0906
HTR2B	GeneTex	GTX70503
PPARγ	Abcam	ab209350
NR2F1	GeneTex	GTX4801
PTGS2	GeneTex	GTX00656
β-actin	Sigma	A5441
Serotonin (5-HT)	Abcam	ab66047
**Chemicals, peptides, and recombinanat proteins**		
Inositol	Sigma	PHR1351
RS-127,445	Merck	R2533
Pioglitazone	Selleckchem	AD-4833
Asenapine maleate	Selleckchem	S1283
Myo-inositol	Selleckchem	S4530
Serotonin powder	Merck	H9523
**Crutical commercial assays**		
Glucose Uptake	Biovision	K676
Lactate	Biovision	K607
**Primers**		
IL1A	Forward	AGATGCCTGAGATACCCAAAACC
IL1A	Reverse	CCAAGCACACCCAGTAGTCT
IL1B	Forward	ATGATGGCTTATTACAGTGGCAA
IL1B	Reverse	GTCGGAGATTCGTAGCTGGA
IL1RL1	Forward	AGAAATCGTGTGTTTGCCTCA
IL1RL1	Reverse	TCCAGTCCTATTGAATGTGGGA
NFKBIA	Forward	ACCTGGTGTCACTCCTGTTGA
NFKBIA	Reverse	CTGCTGCTGTATCCGGGTG
NFKBIE	Forward	GAATTGCTGCTTCGGAATGGA
NFKBIE	Reverse	CATGCGGGCATCTACCTGG
PPARD	Forward	GCCTCTATCGTCAACAAGGAC
PPARD	Reverse	GCAATGAATAGGGCCAGGTC
PTGS2	Forward	CTGGCGCTCAGCCATACAG
PTGS2	Reverse	CGCACTTATACTGGTCAAATCCC
NR2F1	Forward	ATCGTGCTGTTCACGTCAGAC
NR2F1	Reverse	TGGCTCCTCACGTACTCCTC
**Deposited data**		
TCGA	USCS cancer browser	https://genome-cancer.ucsc.edu/proj/site/hgHeatmap
CGGA	GlioVis	https://gliobis.bioinfor.cnio.es/
CCLE	Broad Institute	https://sites.broadinstitute.org/ccle/
**Experimental models: Organisms/strains**		
NOD-SCID	Jackson Laboratories	Strain #:005557
**Software and algorithms**		
SPSS	IBM	17.0
IPA	QIAGEN	N/A
Genspring		13.1.1

### Electronic supplementary material

Below is the link to the electronic supplementary material.

Supplementary Material 1

Supplementary Material 2

Supplementary Material 3

## Data Availability

No datasets were generated or analysed during the current study.

## Abbreviations

2-HG: 2-Hydroxyglutarate
5-AZA: 5-Azacitidine
5-HIAA: 5-Hydroxyindoleacetic acid
ALDOC: Aldolase C
ATRX: Alpha thalassemia/mental retardation syndrome X-linked
BSP: Bisulfite sequencing PCR
CCLE: Cancer Cell Line Encyclopedia
DNMT: DNA methyltransferase
EGFR: Epidermal growth factor receptor
GBM: Glioblastoma
HTR: 5-hydroxytryptamine receptor
IDH1: Isocitrate dehydrogenase 1
IHC: Immunohistochemistry
IPA: Ingenuity Pahtway Analysis
IVIS: In ViVo Imaging System
LGG: Low Grade Glioma
MGMT: O6-methylguanine-DNA methyltransferase
MSP: Methylation-specific PCR
NOD-SCID: Nonobese diabetic/severe combined immunodeficiency
NR2F1: nuclear receptor subfamily 2 group F member 1
SSRI: Selective Serotonin Reuptake Inhibitor
PPAR-γ: Peroxisome proliferator-activated receptor gamma
PTEN: Phosphatase and tensin homolog
PTGS2: prpstag; amdom-endoperoxide synthase 2
Serotonin: 5-hydroxytryptamine
TCA: Tricarboxylic acid cycle
TCGA: The Cancer Genome Atlas
TMZ: Temozolomide
