# Supplementary material for: PPAR-γ agonists reactivate the ALDOC-NR2F1 axis to enhance sensitivity to temozolomide and suppress glioblastoma progression
Source: Cell Commun Signal. 2024 May 13;22:266. doi: 10.1186/s12964-024-01645-3 (PMC11089732; doi:10.1186/s12964-024-01645-3)
Supplement: Supplementary file 1 — Supplementary Material 1 [file 12964_2024_1645_MOESM1_ESM.docx]

**Supplement information**

**PPAR-γ agonist reactivates ALDOC-NR2F1 axis to enhance Temozolomide sensitivity and suppress glioblastoma progression**

Yu-Chan Chang^1*^, Ming-Hsien Chan^1^, Chien-Hsiu Li^2^, Chi Long Chen^3,4^, Wen-Chiuan Tsai^5^ and Michael Hsiao^6^

1. Department of Biomedical Imaging and Radiological Sciences, National Yang Ming Chiao Tung University, Taipei 112, Taiwan
2. Department of Urology, Shuang Ho Hospital, Taipei Medical University, New Taipei City 235, Taiwan
3. Department of Pathology, Taipei Medical University Hospital, Taipei Medical University, Taipei 110, Taiwan
4. Department of Pathology, College of Medicine, Taipei Medical University 110, Taipei,
5. Department of Pathology, Tri-Service General Hospital, National Defense Medical Center, Taipei 114, Taiwan
6. Genomics Research Center, Academia Sinica, Taipei 115, Taiwan

#To whom correspondence should be addressed:

Dr. Yu-Chan Chang, Department of Biomedical Imaging and Radiological Sciences, National Yang Ming Chiao Tung University, Taipei, Taiwan, Tel: +886-2-2826-7064, E-mail: yuchanchang@nycu.edu.tw.

**Supplementary Table and table legends**

**Supplementary Table 1.** A list of transcriptome probes based on ALDOC knockdown.

|  |  | | ALDOC/PTGS2 expression, n(%) | | | | | |
| --- | --- | --- | --- | --- | --- | --- | --- | --- |
| Characteristics | | n | | Low  (n =13) | Medium  (n=19) | High  (n =8) | *P* value |  |
| PDGFRA | |  | |  |  |  |  |  |
| Wild-type  Mutant | | 38  2 | | 13(34.2)  0(0.0) | 17(44.7)  2(100) | 8(21.1)  0(0.0) | 0.741 |  |
| IDH1 | |  | |  |  |  |  |  |
| Wild-type | | 38 | | 13(34.2) | 18(47.4) | 7(18.4) | 0.218 |  |
| R132H mutant | | 2 | | 0(0.0) | 1(50.0) | 1(50.0) |  |  |
| TP53 | |  | |  |  |  |  |  |
| Wild-type | | 16 | | 5(31.3) | 7(43.8) | 4(24.9) | 0.681 |  |
| Mutant | | 24 | | 8(33.3) | 12(50.0) | 4(16.7) |  |  |
| EGFR | |  | |  |  |  |  |  |
| Wild-type | | 39 | | 13(33.3) | 18(46.2) | 8(20.5) | 0.818 |  |
| Mutant | | 1 | | 0(0.0) | 1(100) | 0(0.0) |  |  |
| EGFRvIII | |  | |  |  |  |  |  |
| Wild-type | | 32 | | 10(31.3) | 16(50.0) | 6(18.7) | 0.971 |  |
| Mutant | | 8 | | 3(37.5) | 3(37.5) | 2(25.0) |  |  |
| Neurofilament | |  | |  |  |  |  |  |
| Wild-type | | 36 | | 12(33.3) | 19(52.8) | 5(13.9) | 0.042* |  |
| Mutant | | 4 | | 1(25.0) | 0(0.0) | 3(75.0) |  |  |
| NF1 | |  | |  |  |  |  |  |
| Wild-type | | 22 | | 7(31.8) | 11(50.0) | 4(18.2) | 0.931 |  |
| Mutant | | 18 | | 6(33.3) | 8(44.4) | 4(22.3) |  |  |
| ATRX | |  | |  |  |  |  |  |
| Wild-type | | 18 | | 7(38.9) | 6(33.3) | 5(27.8) | 0.988 |  |
| Loss | | 22 | | 6(27.3) | 13(59.1) | 3(13.6) |  |  |
| H3K27M | |  | |  |  |  |  |  |
| Wild-type | | 30 | | 10(30.0) | 14(46.7) | 6(23.3) | 0.894 |  |
| Mutant | | 10 | | 3(30.0) | 5(50.0) | 2(20.0) |  |  |

**Supplementary Table 2.** Clinical relevance of ALDOC/PTGS2 expression in glioblastoma.

*p value<0.05 was considered statistically significant (Student’s t-test for continuous variables and Pearson’s chi-square test for variables). SD represents the standard deviation. #The tumor stage, tumor, lymph node, and distal metastasis status were classified according to the international system for staging lung cancer.

| Cox univariate analysis (OS) | | |  |  |  |
| --- | --- | --- | --- | --- | --- |
| Variables | | Comparison | HR (95% CI) | | P-value |
| ALDOC/PTGS2 |  | High vs. Low | 0.579 (0.392-0.855) | | 0.006* |
| ALDOC/NR2C1 |  | High vs. Low | 0.610 (0.361-1.029) | | 0.064 |
| Grade |  | T3-T4 vs. T1-T2 | 1.927 (1.321-2.812) | | 0.001* |
| PDGFRA |  | Mutant vs. Wild-type | 0.909 (0.280-2.948) | | 0.909 |
| IDH1-R132H |  | Mutant vs. Wild-type | 1.076 (0.382-3.026) | | 0.890 |
| TP53 |  | Mutant vs. Wild-type | 1.881 (1.033-3.423) | | 0.039* |
| EGFR |  | Mutant vs. Wild-type | 1.219 (0.165-8.977) | | 0.846 |
| EGFRvIII |  | Mutant vs. Wild-type | 1.532 (0.760-3.099) | | 0.233 |
| Neurofilament |  | Mutant vs. Wild-type | 1.023 (0.457-2.288) | | 0.957 |
| NF1 |  | Mutant vs. Wild-type | 1.188 (0.649-2.176) | | 0.577 |
| ATRX |  | Loss vs. Wild-type | 0.625 (0.351-1.112) | | 0.110 |
| H3K27M |  | Mutant vs. Wild-type | 1.599 (0.784-3.261) | | 0.197 |
| Cox multivariate analysis (OS) | | |  |  |  |
| Variables | | Comparison | HR (95% CI) | | P-value |
| ALDOC/PTGS2 |  | High vs. Low | 0.545 (0.320-0.929) | | 0.026* |
| ALDOC/NR2C1 |  | High vs. Low | 0.862 (0.441-1.682) | | 0.663 |
| Grade |  | T3-T4 vs. T1-T2 | 2.791 (1.618-4.815) | | <0.001* |
| PDGFRA |  | Mutant vs. Wild-type | 2.109 (0.525-8.480) | | 0.293 |
| IDH1-R132H |  | Mutant vs. Wild-type | 1.432 (0.416-4.931) | | 0.569 |
| TP53 |  | Mutant vs. Wild-type | 1.014 (0.507-2.026) | | 0.968 |
| EGFR |  | Mutant vs. Wild-type | 2.967 (0.312-28.24) | | 0.344 |
| EGFRvIII |  | Mutant vs. Wild-type | 1.548 (0.677-3.536) | | 0.300 |
| Neurofilament |  | Mutant vs. Wild-type | 4.373 (1.450-13.19) | | 0.009* |
| NF1 |  | Mutant vs. Wild-type | 0.379 (0.161-0.893) | | 0.026* |
| ATRX |  | Loss vs. Wild-type | 0.467 (0.218-1.002) | | 0.050* |
| H3K27M |  | Mutant vs. Wild-type | 0.691 (0.272-1.755) | | 0.437 |

**Supplementary Table 3.** Univariate and multivariate analyses for target genes expression in glioblastoma.

**Supplementary Table 4.** Various parameters and relationships in GBM cell lines.

| Cell lines | Methylation status | ALDOC expression | Serotonin production | Migration ability | PPAR-γ expression | PTGS2 expression | NR2F1 expression |
| --- | --- | --- | --- | --- | --- | --- | --- |
| A172 | High | Low | High | High | Low | High | Low |
| LN-229 | High | Low | High | High | Low | High | Low |
| U-87MG | Low | High | Low | Low | High | Low | High |
| SW1088 | Low | High | Low | Low | High | Low | High |

**Supplementary Table 5.** List of primers for Q-PCR.

| Name | Sequence |
| --- | --- |
| IL1A-5' | AGATGCCTGAGATACCCAAAACC |
| IL1A-3' | CCAAGCACACCCAGTAGTCT |
| IL1B-5' | ATGATGGCTTATTACAGTGGCAA |
| IL1B-3' | GTCGGAGATTCGTAGCTGGA |
| IL1RL1-5' | AGAAATCGTGTGTTTGCCTCA |
| IL1RL1-3' | TCCAGTCCTATTGAATGTGGGA |
| NFKBIA-5' | ACCTGGTGTCACTCCTGTTGA |
| NFKBIA-3' | CTGCTGCTGTATCCGGGTG |
| NFKBIE-5' | GAATTGCTGCTTCGGAATGGA |
| NFKBIE-3' | CATGCGGGCATCTACCTGG |
| PPARD-5' | GCCTCTATCGTCAACAAGGAC |
| PPARD-3' | GCAATGAATAGGGCCAGGTC |
| PTGS2-5' | CTGGCGCTCAGCCATACAG |
| PTGS2-3' | CGCACTTATACTGGTCAAATCCC |
| NR2F1-5' | ATCGTGCTGTTCACGTCAGAC |
| NR2F1-3' | TGGCTCCTCACGTACTCCTC |

**Supplementary Figure and figure legends**

**Figure S1.** The expression level of ALDOC in histological classification of brain tumors. This database extracted ALDOC profiles from CGGA RNA-seq files. **
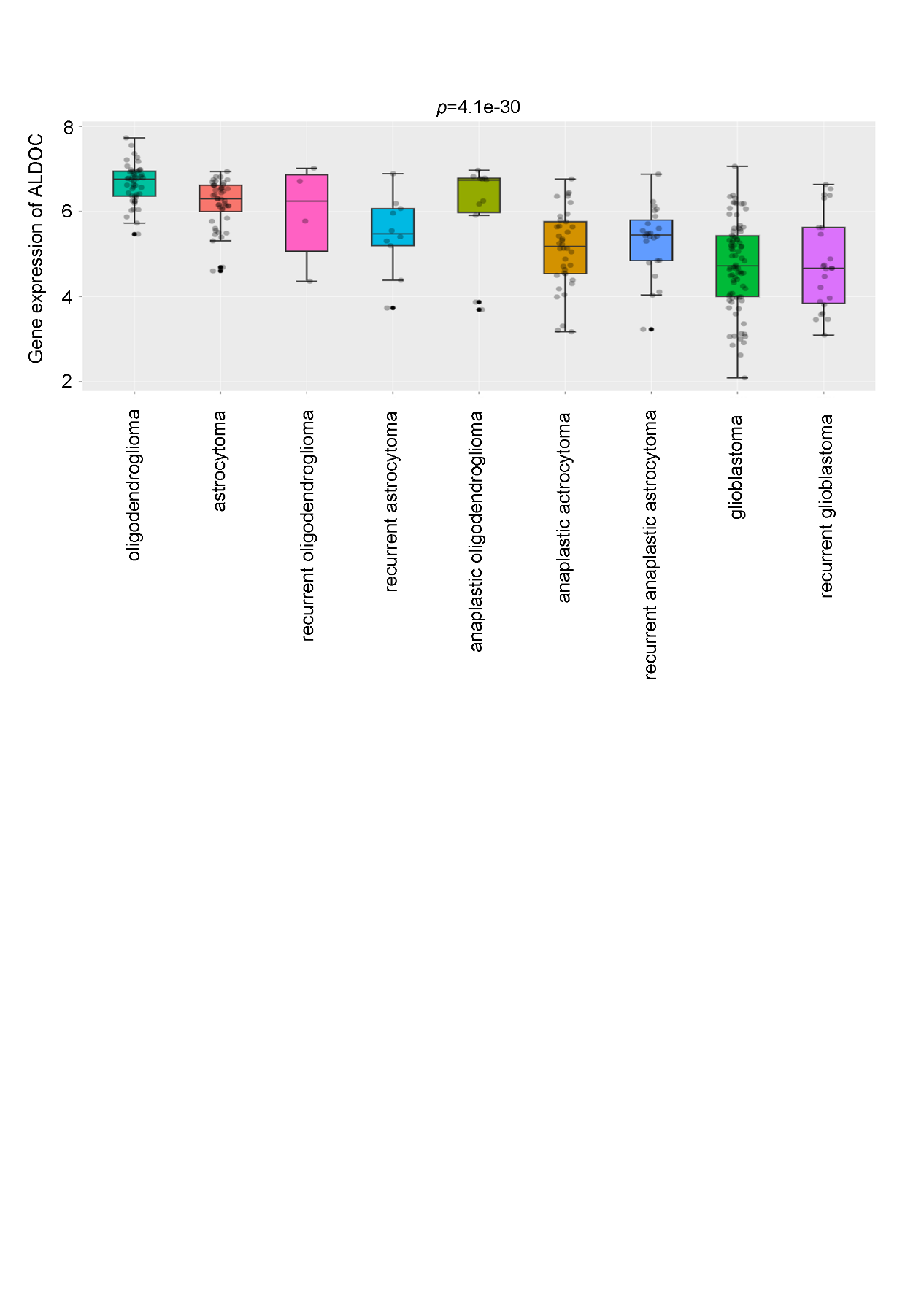
**

**Figure S2. (A)** The calculation of the glucose consumption in the ALDOC knockdown stable cells. The data from three independent experiments as the means ± SEM. **(B)** The calculation of the glucose consumption in the GBM cells with or without 5-aza treatment. The data from three independent experiments as the means ± SEM.

**
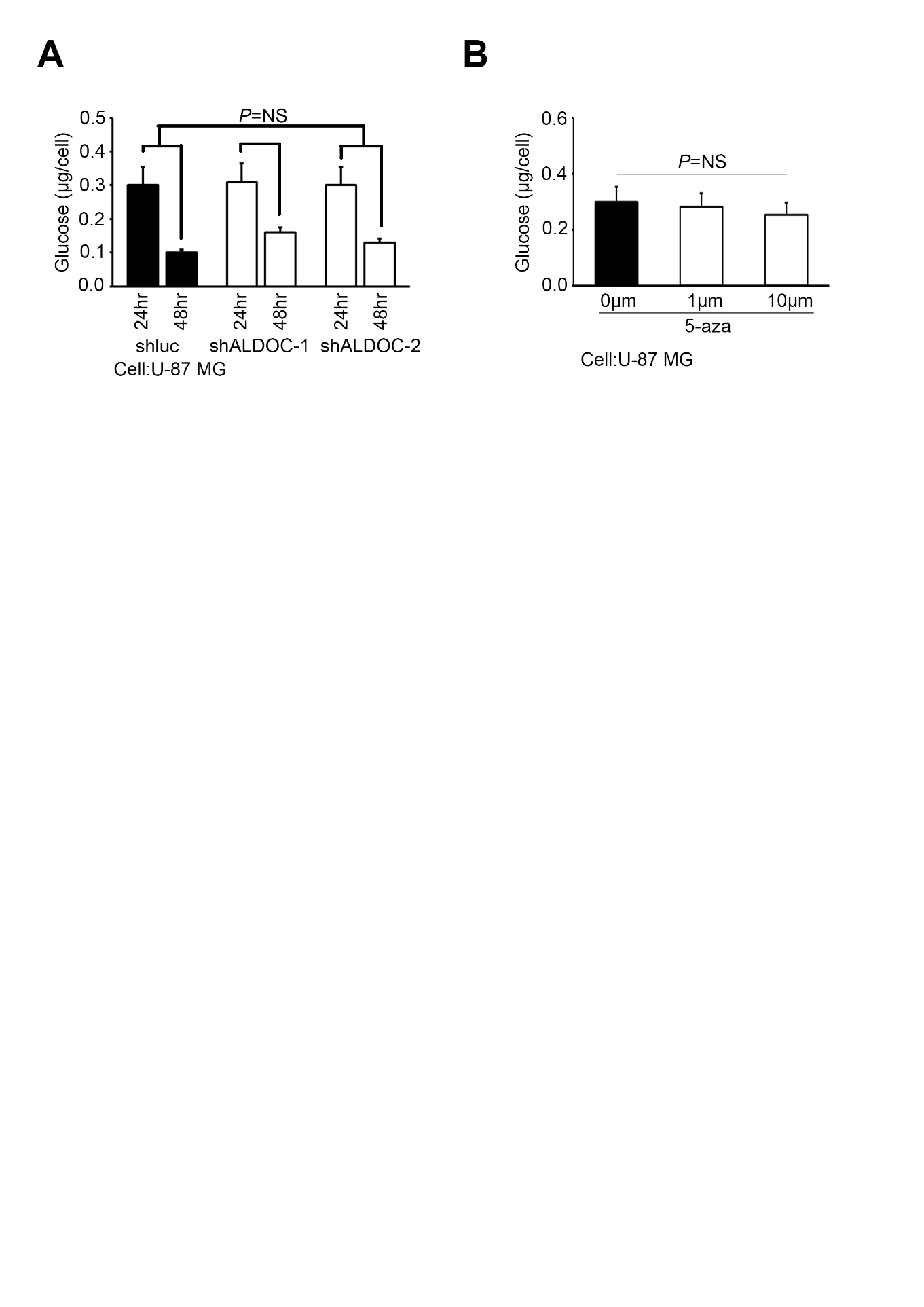
**

**Figure S3.** Metabolite dependent association analysis revealed several metabolites in GBM cells with altered ALDOC methylation using the CCLE repository. Red represents positive dependence of ALDOC on metabolite production, while blue represents negative dependence.


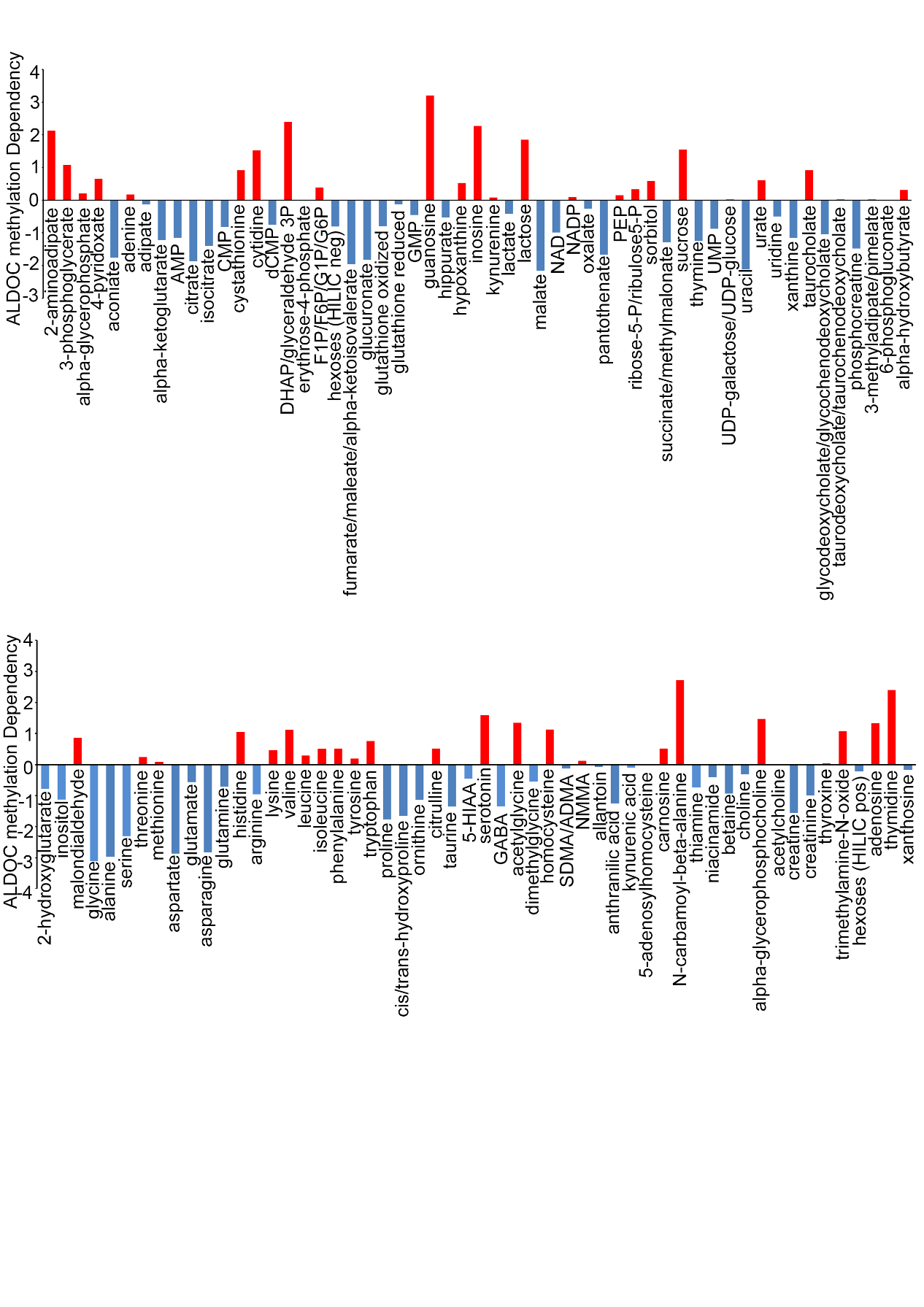


**Figure S4.** (A) Proliferation ability of ALDOC knockdown and overexpression model, respectively. (B-C) Migration/invasion ability of U-87MG cells and SW1088 cells with shLuc or shALDOC expression, respectively. The data from three independent experiments are presented in A, B, and C as the means ± SEM. The significance of the difference was analyzed using the nonparametric Mann-Whitney *U*-test. In B and C, the blue and green columns represent cellular migration and invasion abilities, respectively.

**
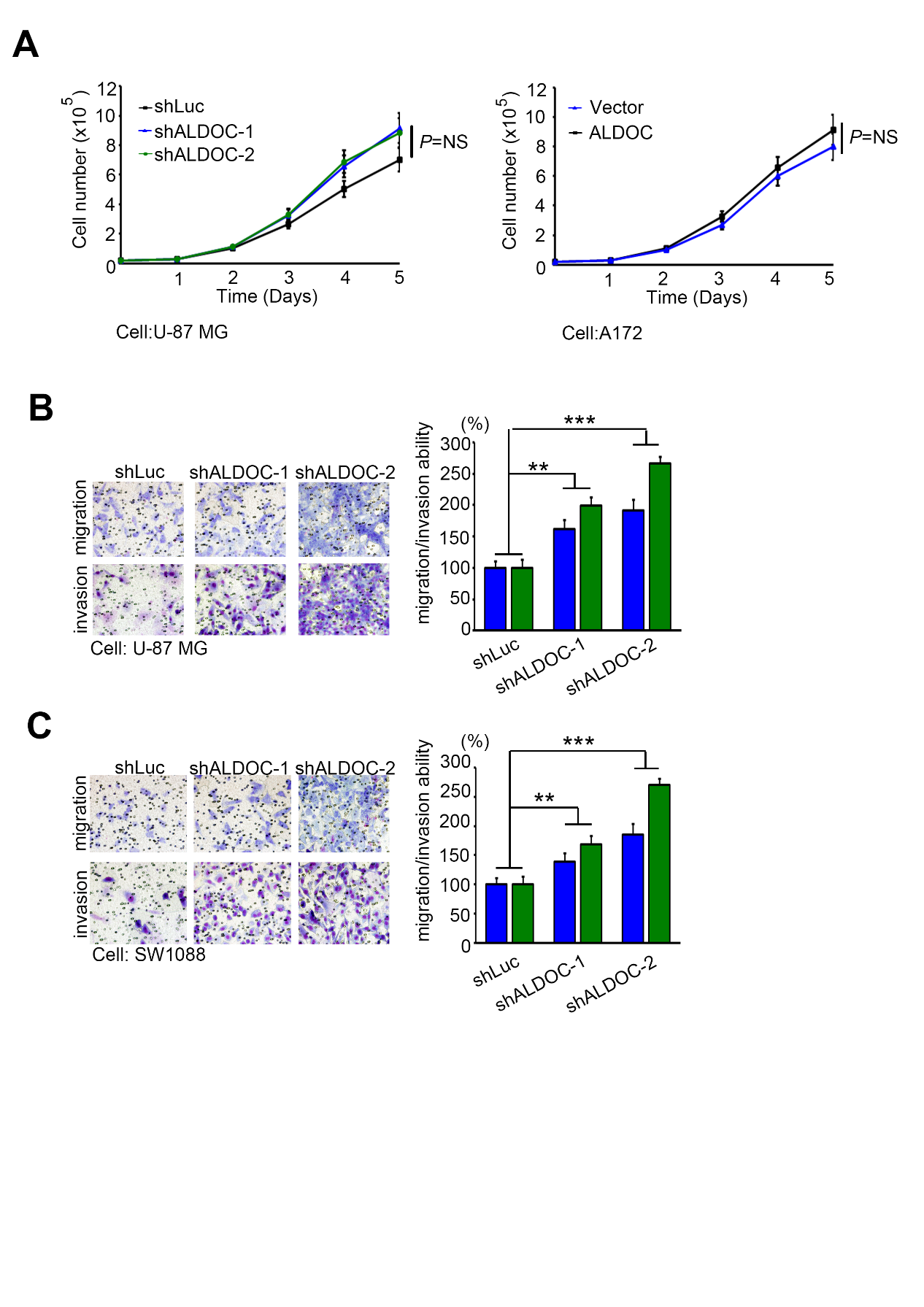
**

**Figure S5.** (A) Migration/invasion ability in U-87MG with serotonin (1μM and 10μM) treatment. (B) Proliferation ability of U-87MG with serotonin (1μM and 10μM) treatment. The data from three independent experiments are presented in A and B as the means ± SEM. The significance of the difference was analyzed using the nonparametric Mann-Whitney *U*-test.


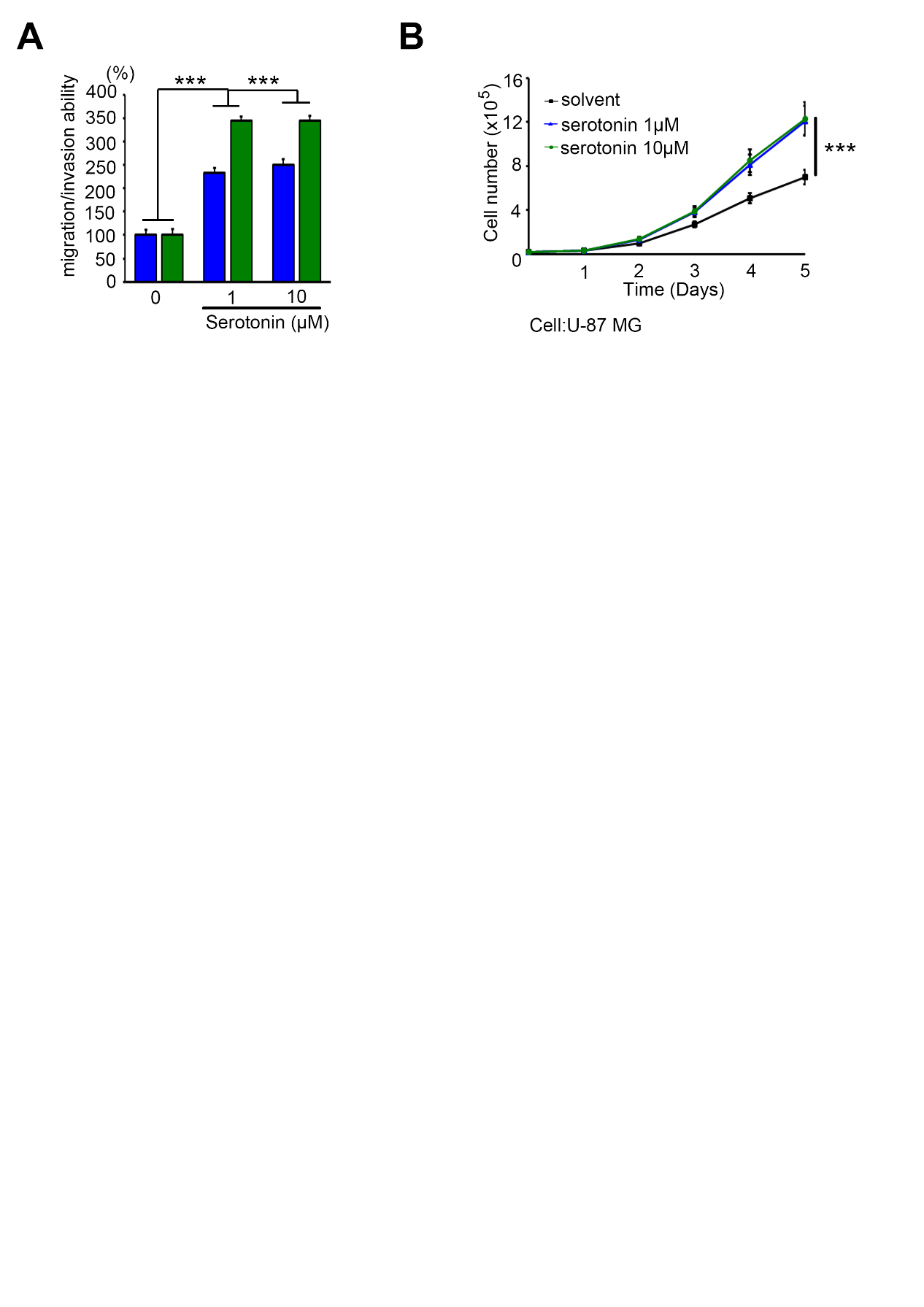


**Figure S6.** (A) Immunofluorescence assay of U-87MG with serotonin treatment (1μM and 10μM). Red: serotonin; Blue: DAPI. Scale bar: 20μM. (B) Immunofluorescence assay of U-87MG with or without ALDOC knockdown. Red: serotonin; Blue: DAPI. Scale bar: 20μM.


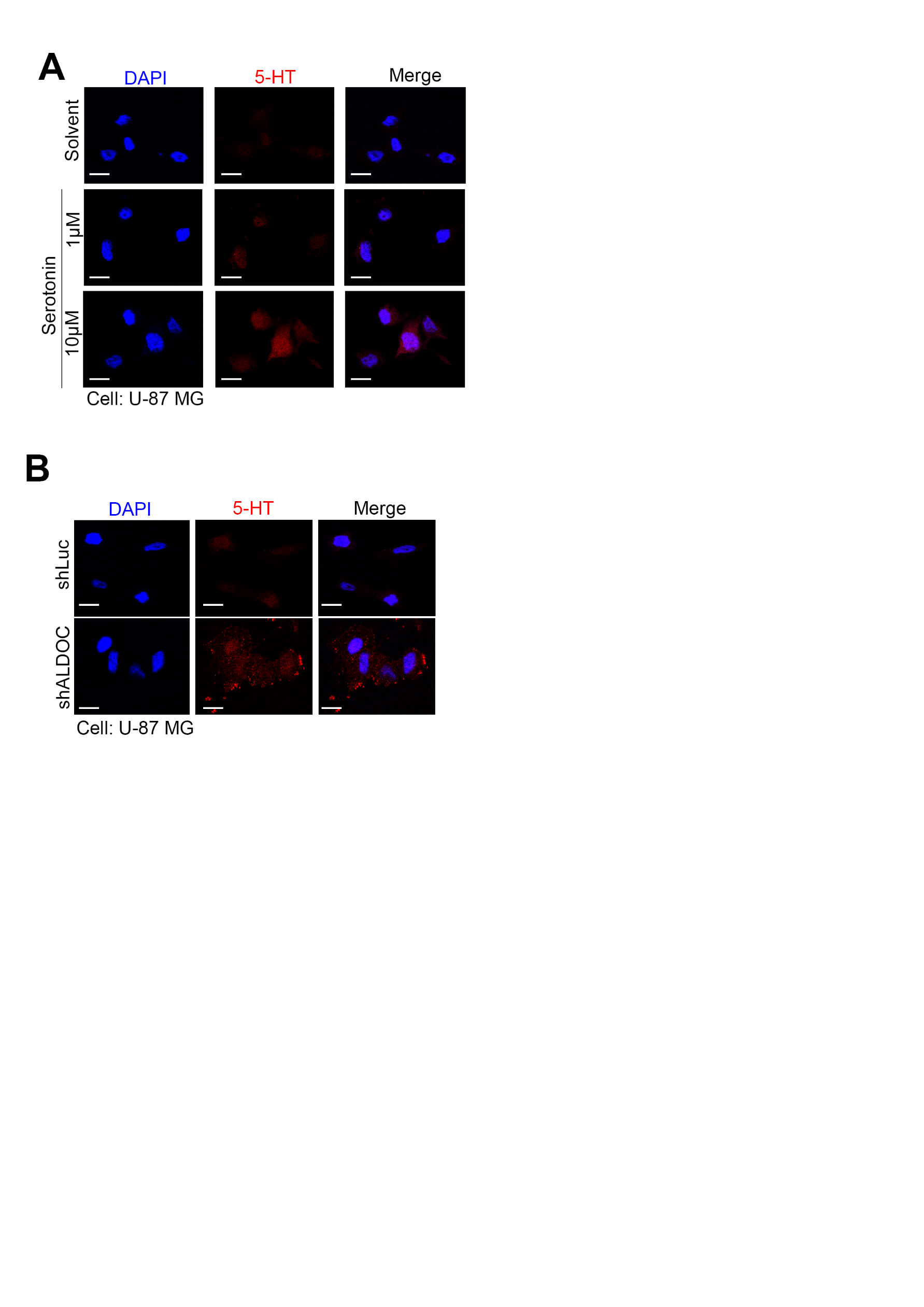


**Figure S7.** Alamar blue assay measures cell viability in U-87MG cells and T98G cell with serotonin (1μM and 10μM) treatment, respectively.

**
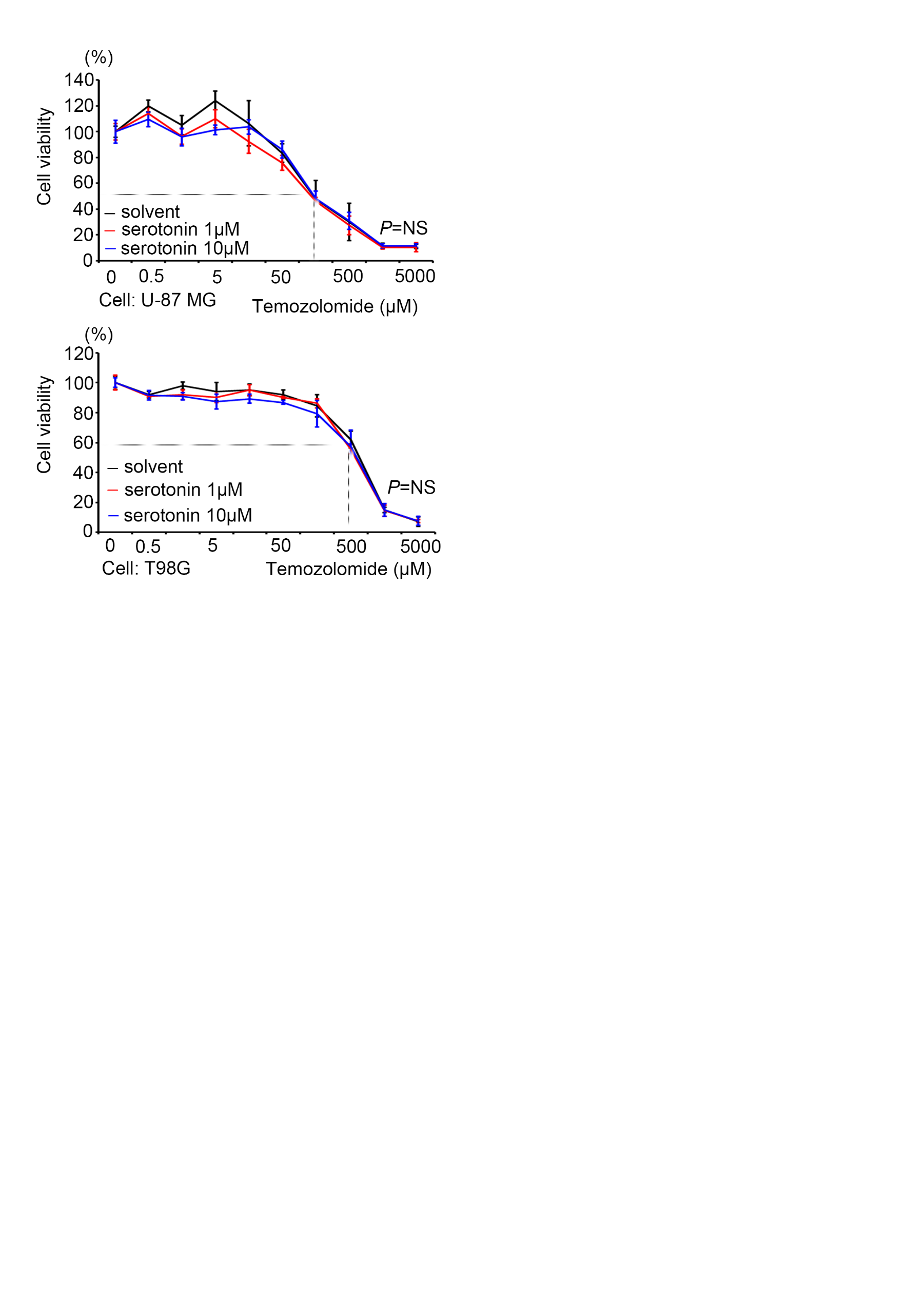
**

**Figure S8.** Giemsa staining to evaluate the migration abilities of U-87MG cells with inositol or *Myo*-inositol (1μM ~10mM) treatment. Scale bar: 100μM.


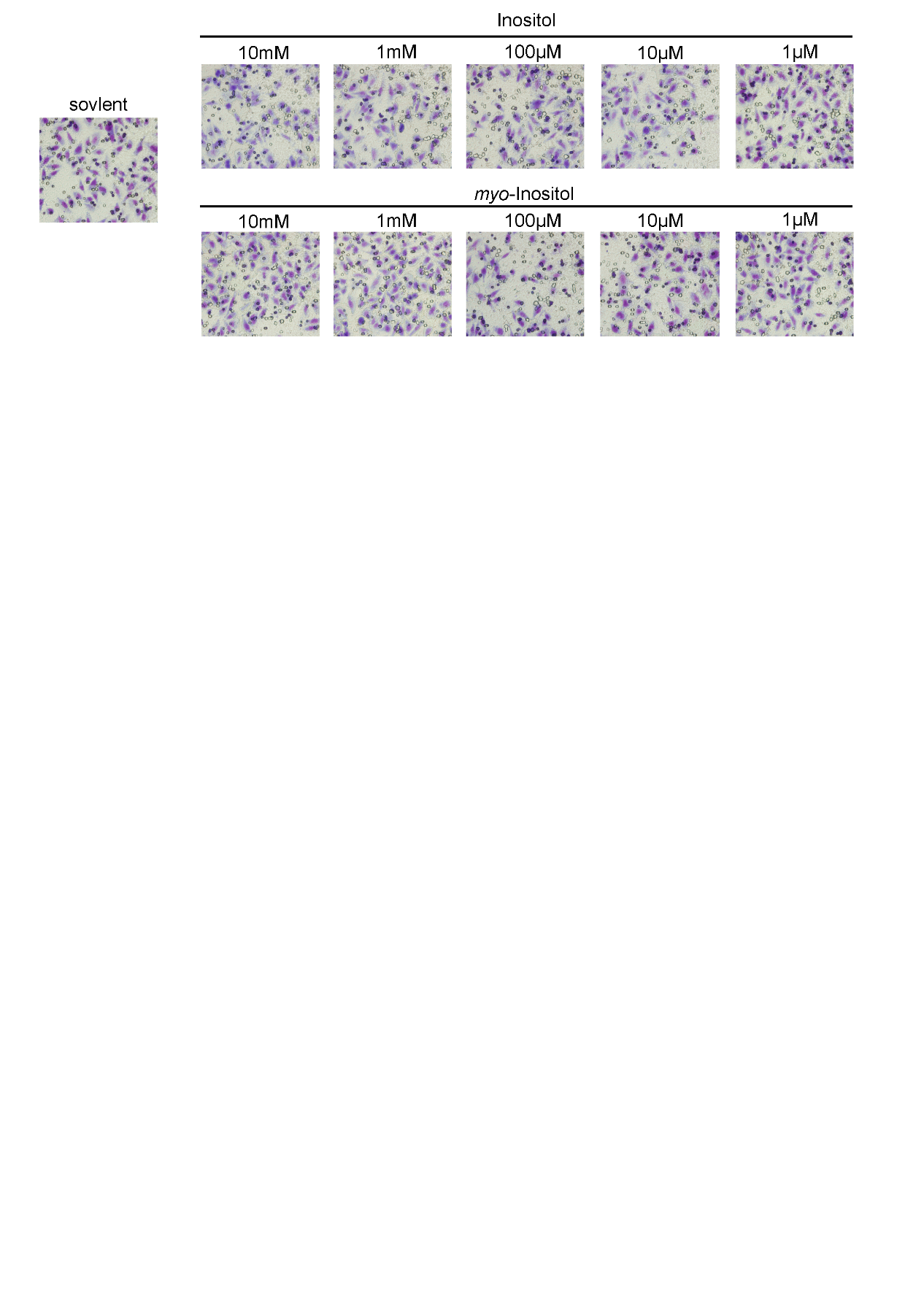


**Figure S9.** Quantification of the expression level of PPARγ downstream targets (*NFKB1A*, and *PTGS2*) in ALDOC overexpression stable cells. The data from three independent experiments as the means ± SEM. The significance of the difference was analyzed using the nonparametric Mann-Whitney *U*-test.


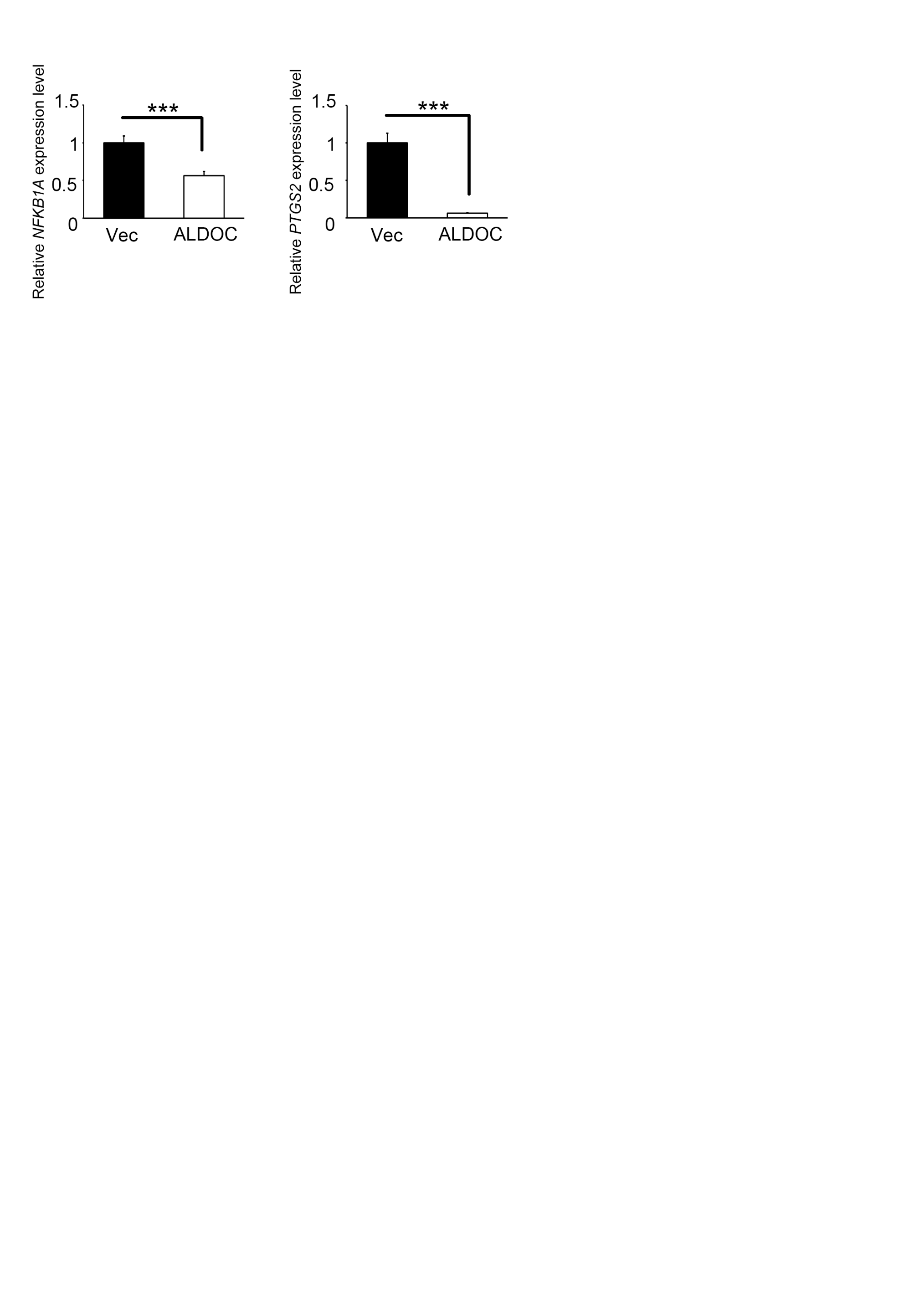


**Figure S10.** (A) Giemsa staining to evaluate the migration abilities of U-87MG shALDOC cells with 5-HT receptors inhibitors (RS-127445 and Asenapine maleate) treatment. Scale bar: 100μM. (B) Immunofluorescence assay of U-87MG shALDOC cells after Asenapine maleate treatment. Red: serotonin; Blue: DAPI. Scale bar: 20μM. (C) Migration ability of U-87MG with RS-127445 or RS-127445 combined with serotonin (1μM or 10μM) in U-87MG shALDOC cells. The data from three independent experiments are presented as the means ± SEM. The significance of the difference was analyzed using the nonparametric Mann-Whitney *U*-test.


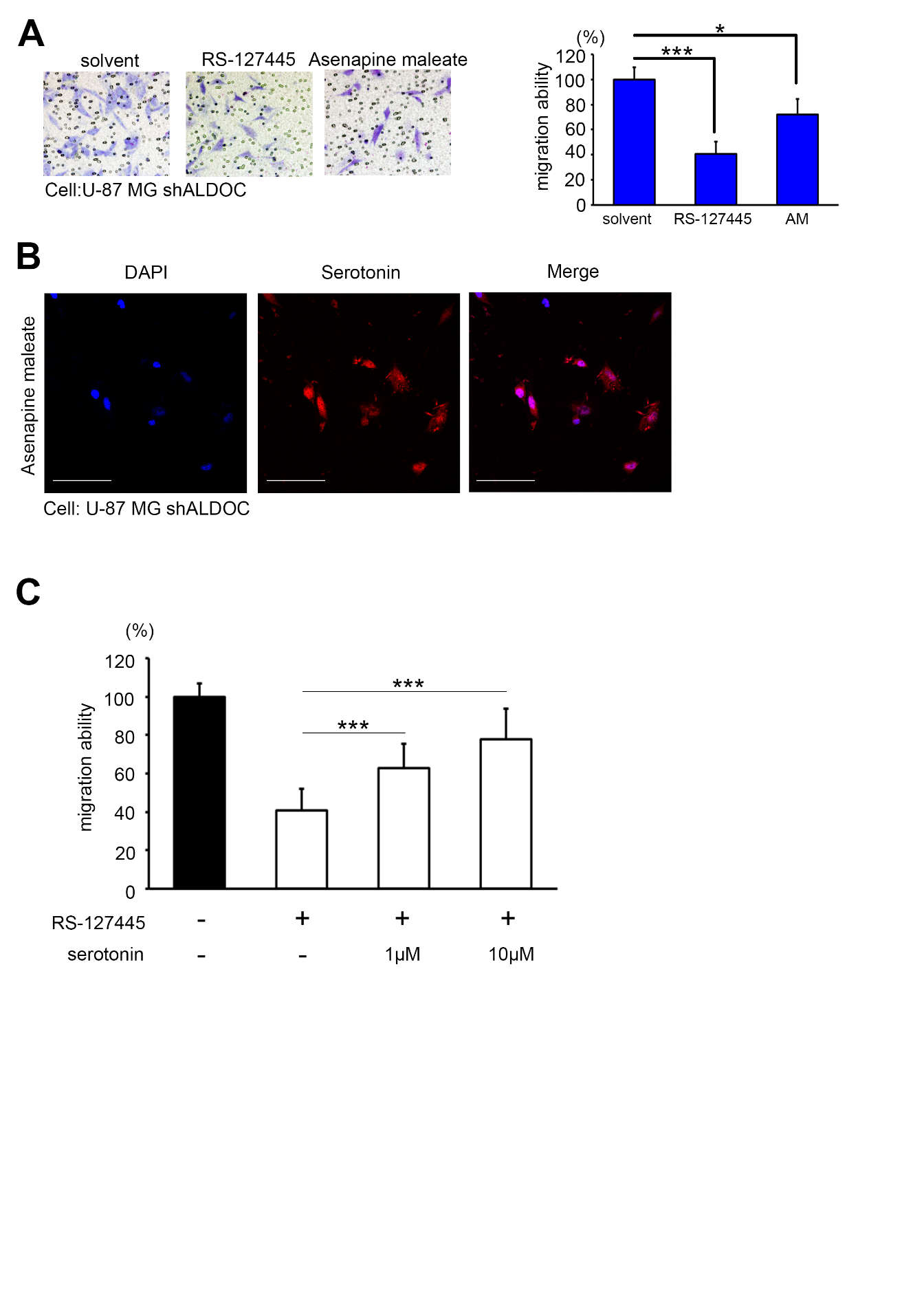


**Figure S11.** After whole-brain extraction, there was a representative IF staining of several candidate proteins in the LN-229 and LN-229 shALDOC intracranial model. Green: PTGS2; Blue: DAPI. Scale bar: 150μM.


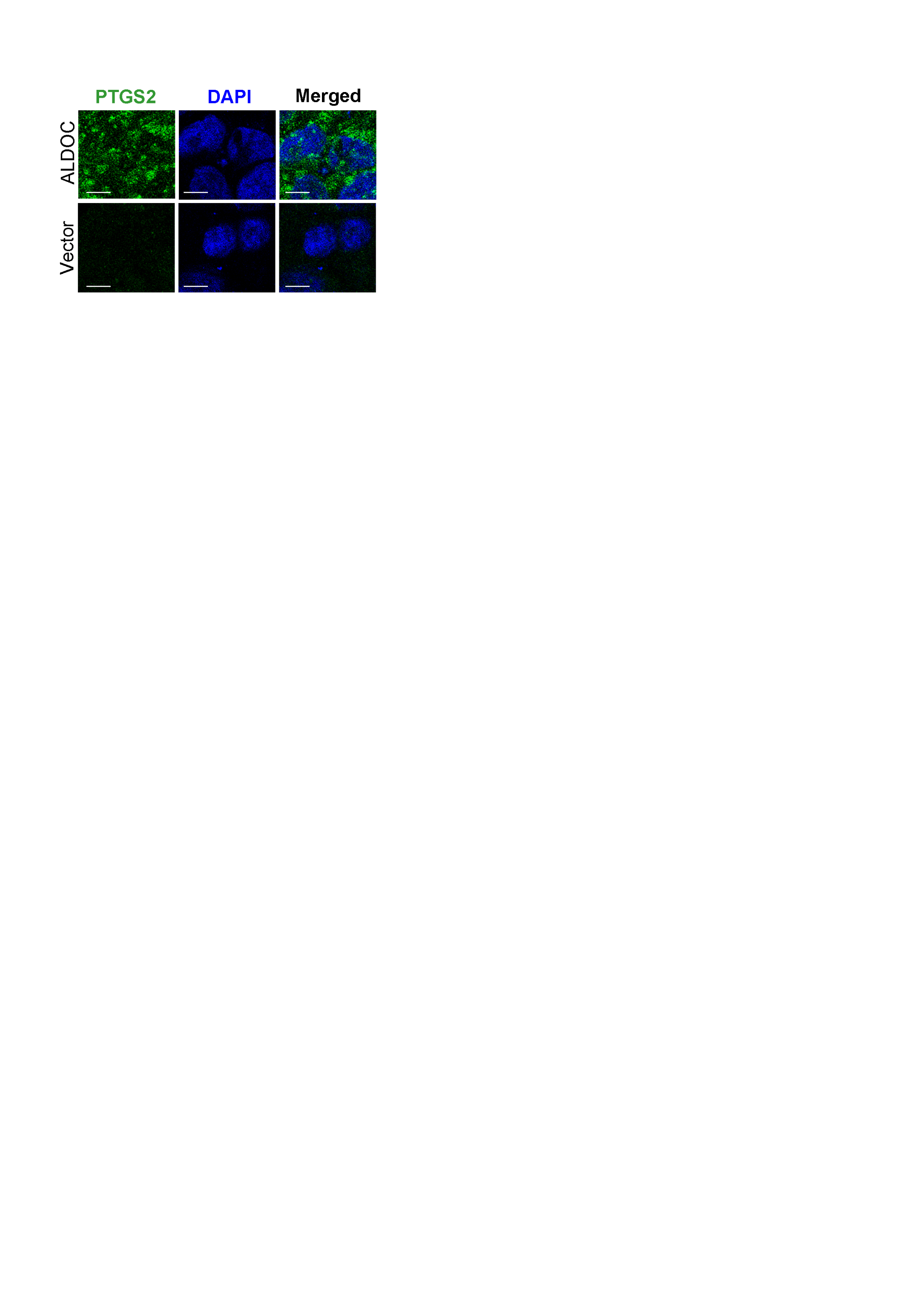


**Figure S12.** Alamar blue assay to measure cell viabilities in A172 cells with various compounds, including serotonin, RS-127445, asenapine maleate, GW0742, and pioglitazone, respectively. The data from three independent experiments as the means ± SEM.


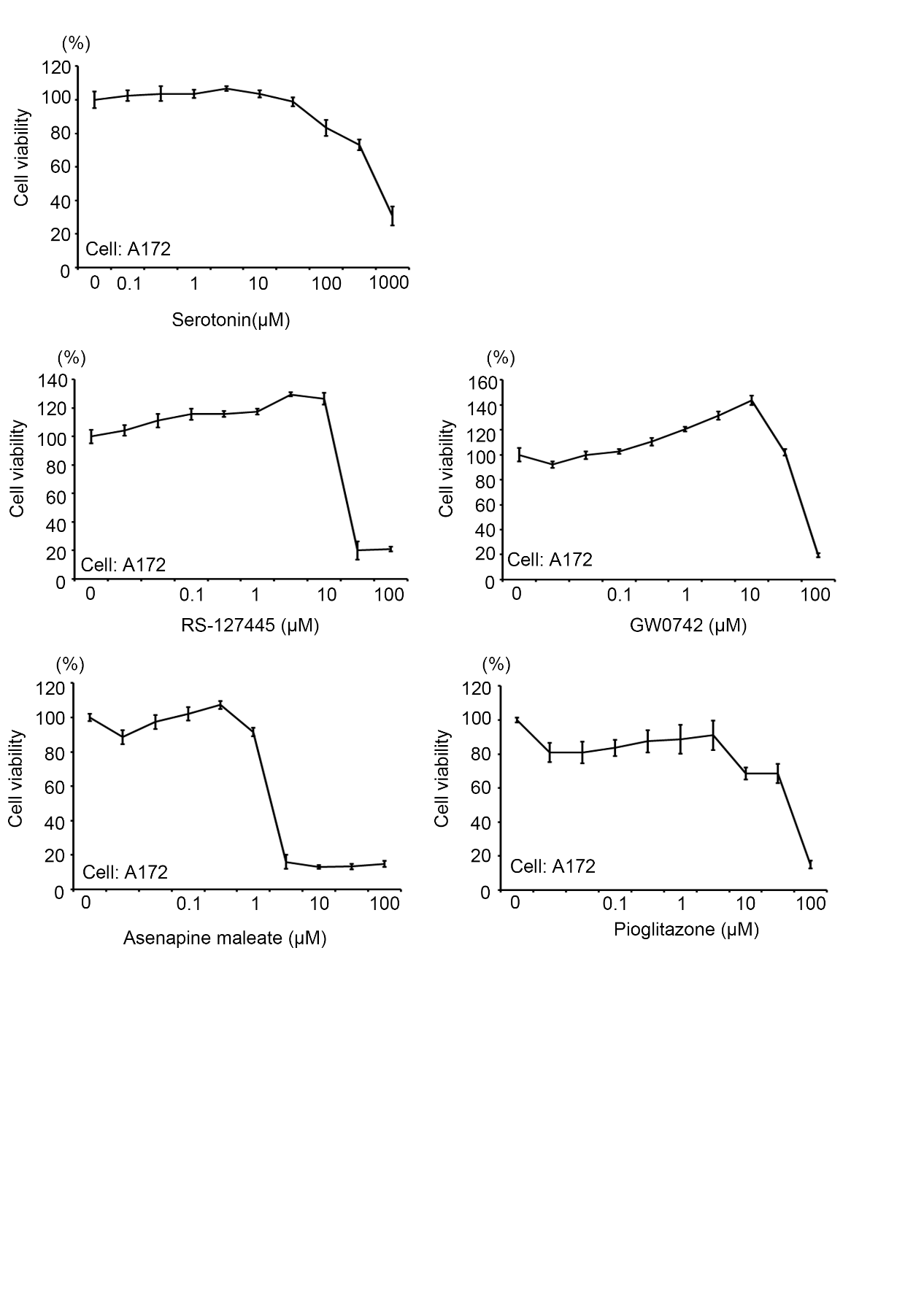


**Figure S13.** (A) Immunofluorescence assay of U-87MG shALDOC cells after PPARγ agonists (GW0742 and Pioglitazone) treatment. Red: serotonin; Blue: DAPI. Scale bar: 20μM. (B) Migration ability of U-87MG with GW0742 or GW0742 combined with serotonin (1μM or 10μM) in U-87MG shALDOC cells. The data from three independent experiments are presented as the means ± SEM. The significance of the difference was analyzed using the nonparametric Mann-Whitney *U*-test.

**
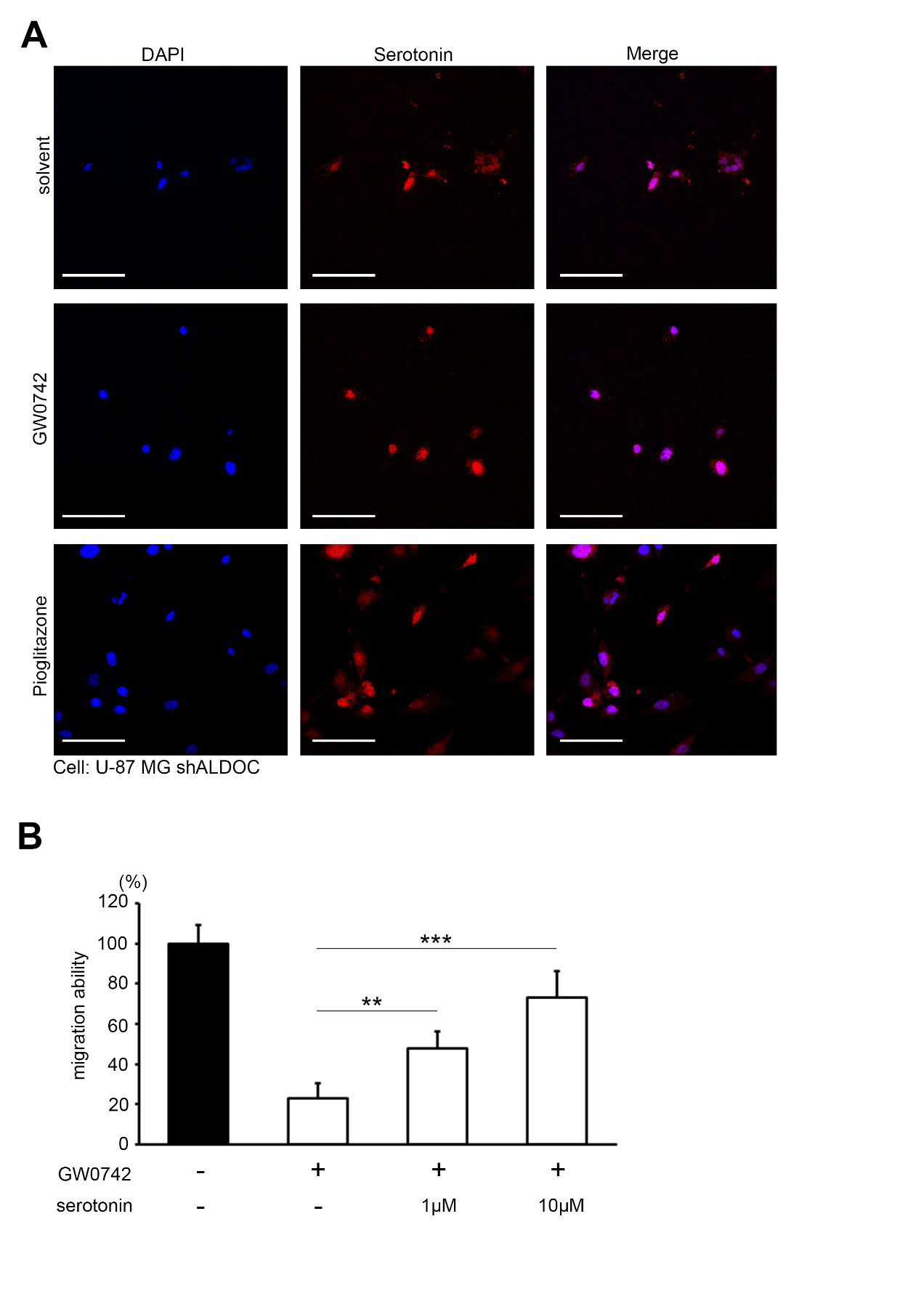
**

**Figure S14.** Protein levels of PPARγ and its downstream targets (NR2F1 and PTGS2) in U-87MG ALDOC shLuc stable cells were detected by Western blotting, with or without RS-127445/GW0742 treatment. Actin was used as an internal control.


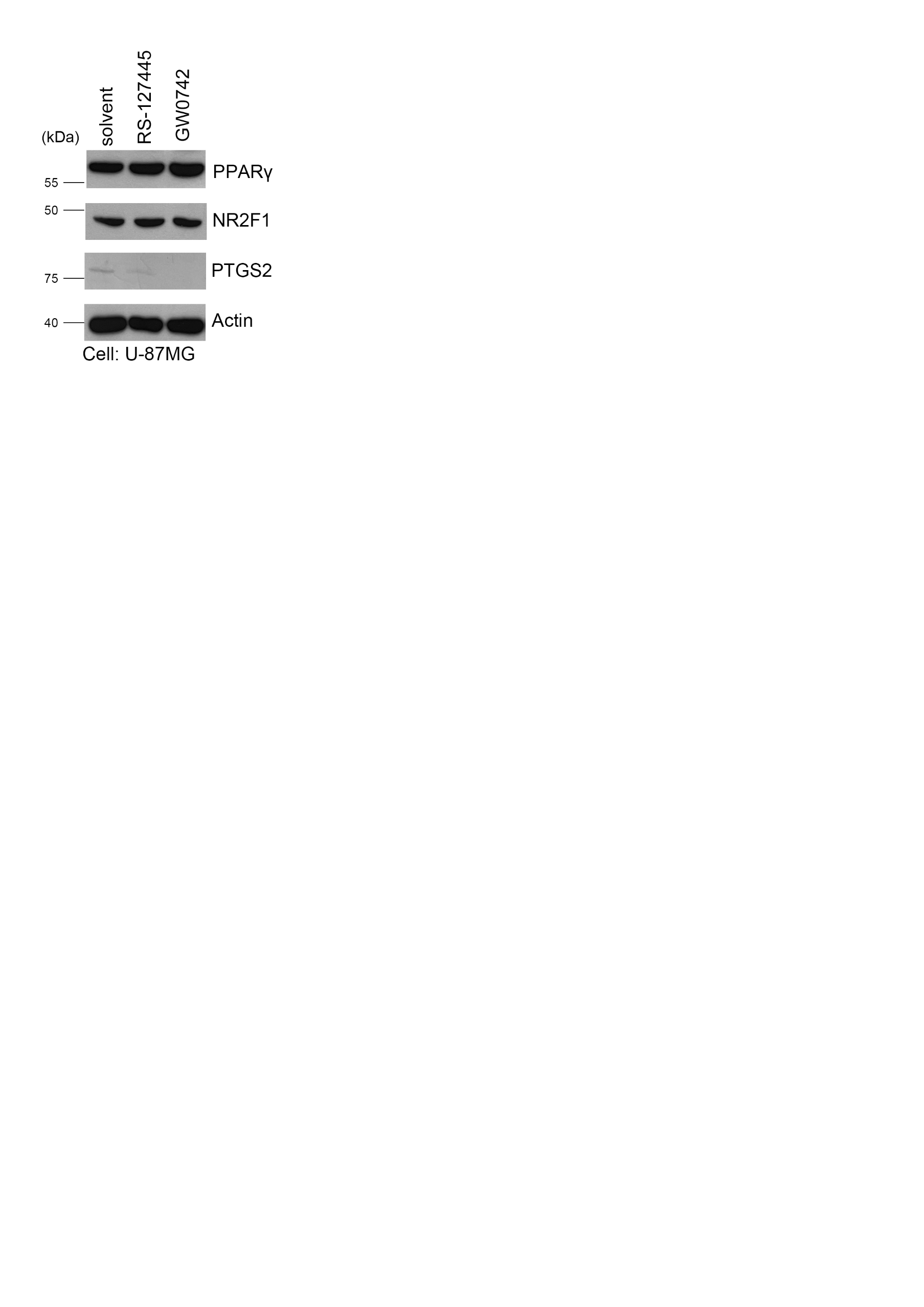


**Figure S15.** Correlation plots between Temozolomide IC50 and ALDOC/PPARG expression levels in GBM cells from the DepMap portal website.


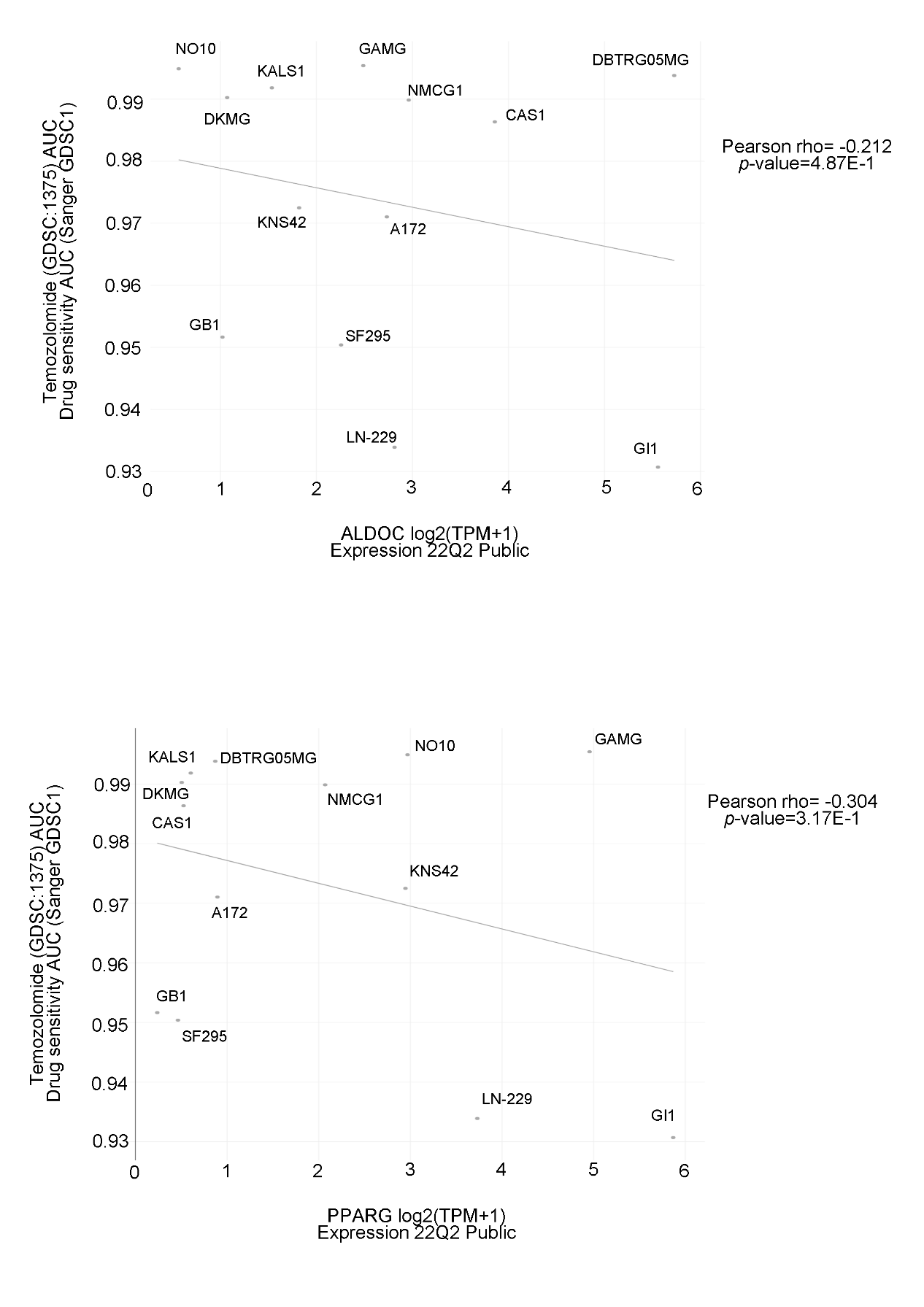


**Figure S16.** Correlation between *ALDOC* expression and *PTGS2/NR2F1/HTR2B* expression in TCGA GBM cohort. A nonparametric Spearman correlation analysis was used to evaluate the significance of the correlation. The significance of the correlation was analyzed using the nonparametric Spearman method.

**
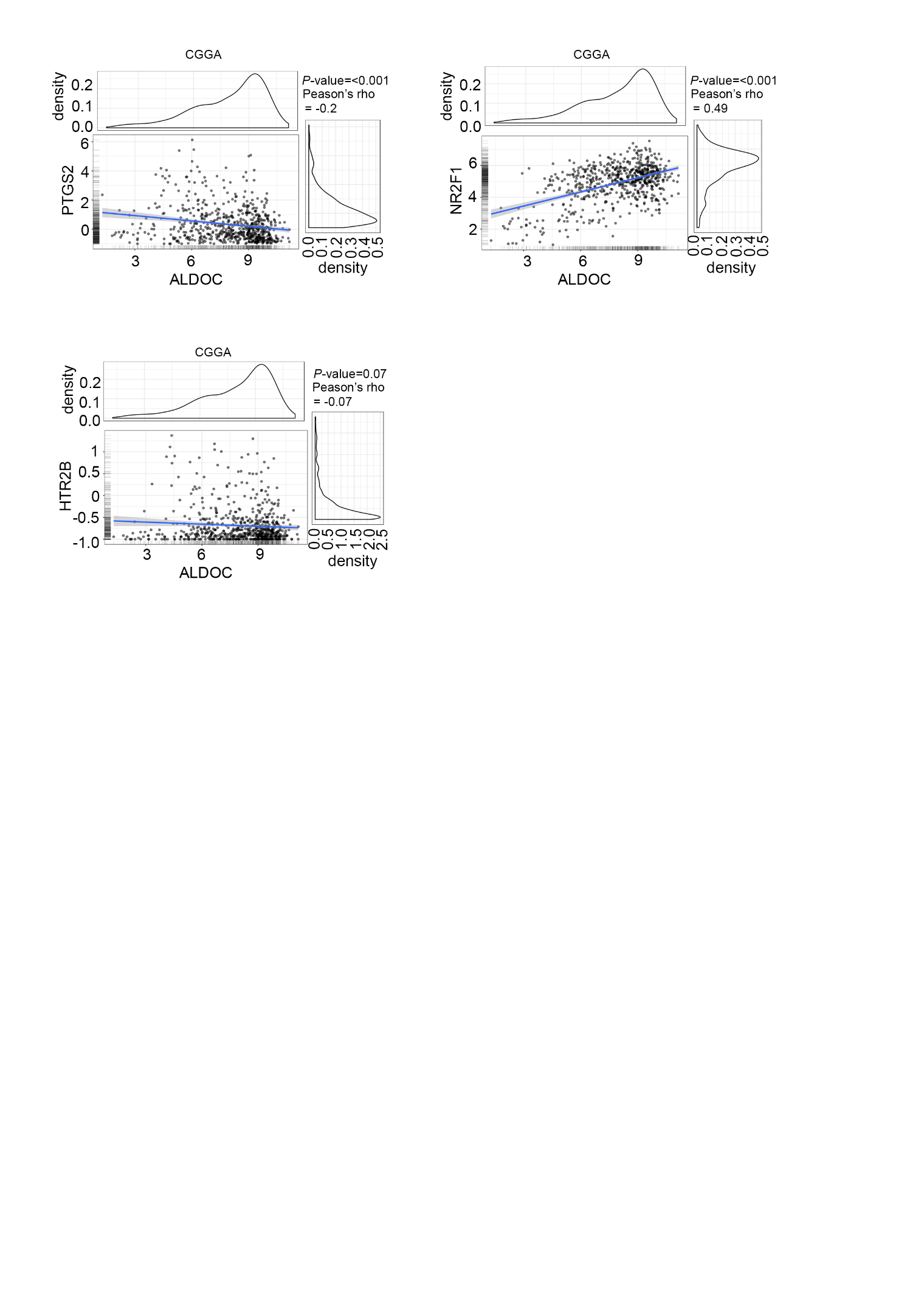
**
