# Supplementary material for: PPAR-γ agonists reactivate the ALDOC-NR2F1 axis to enhance sensitivity to temozolomide and suppress glioblastoma progression
Source: Cell Commun Signal. 2024 May 13;22:266. doi: 10.1186/s12964-024-01645-3 (PMC11089732; doi:10.1186/s12964-024-01645-3)
Supplement: Supplementary file 2 — Supplementary Material 2 [file 12964_2024_1645_MOESM2_ESM.pdf]

Supplementary Table S1. A list of transcriptome probes based on ALDOC knockdown.												
Probe Set ID	FC ([U87	Log FC ([	FC (abs) (	Regulation	FC ([U87	Log FC ([	FC (abs) (	Regulation	U87 con.C	U87 shAL	U87 shAL	Gene Symbol
1565823_at	-1.54968	-0.63197	1.549677	down	-1.58271	-0.66239	1.582705	down	0	-0.63197	-0.66239	7-Sep
230071_at	-1.52074	-0.60477	1.520735	down	-1.70225	-0.76745	1.702254	down	0	-0.60477	-0.76745	11-Sep
222689_at	-1.67461	-0.74383	1.67461	down	-1.52749	-0.61116	1.52749	down	0	-0.74383	-0.61116	ACER3
1557418_at	-1.56662	-0.64766	1.566623	down	-1.88563	-0.91505	1.885634	down	0	-0.64766	-0.91505	ACSL4
202666_s_at	-1.65061	-0.723	1.650608	down	-1.74966	-0.80708	1.749661	down	0	-0.723	-0.80708	ACTL6A
213411_at	-1.82796	-0.87024	1.827963	down	-1.65001	-0.72248	1.650012	down	0	-0.87024	-0.72248	ADAM22
232395_x_at	-1.91252	-0.93547	1.912519	down	-1.67225	-0.74179	1.672251	down	0	-0.93547	-0.74179	AGBL3
228932_at	-1.55388	-0.63588	1.55388	down	-1.5903	-0.6693	1.5903	down	0	-0.63588	-0.6693	AGO3
205002_at	-1.50088	-0.58581	1.500881	down	-1.50088	-0.58581	1.500881	down	0	-0.58581	-0.58581	AHDC1
226665_at	-1.70974	-0.77378	1.709744	down	-1.52145	-0.60545	1.521449	down	0	-0.77378	-0.60545	AHSA2
225342_at	-1.50849	-0.5931	1.508487	down	-1.73787	-0.79732	1.737871	down	0	-0.5931	-0.79732	AK4
209160_at	-1.58184	-0.6616	1.581836	down	-1.7802	-0.83204	1.780199	down	0	-0.6616	-0.83204	AKR1C3
202022_at	-2.7126	-1.43968	2.712603	down	-1.71941	-0.78191	1.719406	down	0	-1.43968	-0.78191	ALDOC
228941_at	-1.95323	-0.96586	1.953233	down	-2.08693	-1.06138	2.086932	down	0	-0.96586	-1.06138	ALG10B
242900_at	-1.7421	-0.80083	1.7421	down	-1.91807	-0.93965	1.918068	down	0	-0.80083	-0.93965	ALG10B
235926_at	-1.74437	-0.8027	1.744366	down	-1.74687	-0.80477	1.746869	down	0	-0.8027	-0.80477	ANAPC5
220076_at	-1.87975	-0.91054	1.87975	down	-1.74166	-0.80046	1.741661	down	0	-0.91054	-0.80046	ANKH
220940_at	-2.04122	-1.02943	2.041221	down	-2.08482	-1.05993	2.084824	down	0	-1.02943	-1.05993	ANKRD36B
226641_at	-2.94408	-1.55782	2.944081	down	-1.65192	-0.72414	1.651921	down	0	-1.55782	-0.72414	ANKRD44
228471_at	-2.01536	-1.01104	2.015358	down	-1.66771	-0.73787	1.667707	down	0	-1.01104	-0.73787	ANKRD44
201038_s_at	-1.55073	-0.63295	1.550734	down	-1.57873	-0.65876	1.578727	down	0	-0.63295	-0.65876	ANP32A
230652_at	-1.60292	-0.6807	1.602919	down	-1.50981	-0.59436	1.509805	down	0	-0.6807	-0.59436	ARAF
204492_at	-1.50088	-0.58581	1.500881	down	-1.5622	-0.64358	1.562196	down	0	-0.58581	-0.64358	ARHGAP11A
1558750_a_at	-1.55425	-0.63622	1.554253	down	-1.54585	-0.6284	1.545849	down	0	-0.63622	-0.6284	ARHGAP11B///LOC100288637
226576_at	-1.50539	-0.59014	1.505388	down	-2.03145	-1.02251	2.031451	down	0	-0.59014	-1.02251	ARHGAP26
233903_s_at	-1.6804	-0.7488	1.680399	down	-1.6804	-0.7488	1.680399	down	0	-0.7488	-0.7488	ARHGEF26
224797_at	-1.63489	-0.7092	1.634894	down	-1.73138	-0.79192	1.731377	down	0	-0.7092	-0.79192	ARRDC3
219973_at	-1.6216	-0.69742	1.621604	down	-1.63202	-0.70666	1.632021	down	0	-0.69742	-0.70666	ARSJ
237968_at	-1.51151	-0.59599	1.511512	down	-1.74206	-0.80079	1.742059	down	0	-0.59599	-0.80079	ATL2
226302_at	-1.76009	-0.81565	1.760086	down	-2.03761	-1.02688	2.037613	down	0	-0.81565	-1.02688	ATP8B1
235240_at	-1.51387	-0.59824	1.513873	down	-1.54982	-0.6321	1.549815	down	0	-0.59824	-0.6321	ATXN3
211379_x_at	-1.55838	-0.64005	1.558383	down	-1.51749	-0.60168	1.517485	down	0	-0.64005	-0.60168	B3GALNT1
227051_at	-1.81608	-0.86083	1.816081	down	-2.18275	-1.12615	2.182749	down	0	-0.86083	-1.12615	BACE2
232442_at	-2.03501	-1.02503	2.035007	down	-2.16185	-1.11226	2.161847	down	0	-1.02503	-1.11226	BCAR1
230722_at	-1.55148	-0.63364	1.55148	down	-1.62354	-0.69914	1.623539	down	0	-0.63364	-0.69914	BNC2
224471_s_at	-1.52005	-0.60412	1.520053	down	-1.75742	-0.81346	1.75742	down	0	-0.60412	-0.81346	BTRC
237245_at	-2.08478	-1.0599	2.084781	down	-1.86744	-0.90106	1.86744	down	0	-1.0599	-0.90106	BUB3
209183_s_at	-2.24911	-1.16936	2.249115	down	-2.99616	-1.58312	2.996161	down	0	-1.16936	-1.58312	C10orf10
65472_at	-1.80608	-0.85286	1.806079	down	-1.66215	-0.73305	1.662145	down	0	-0.85286	-0.73305	C2orf68
224605_at	-1.75364	-0.81035	1.753639	down	-2.13956	-1.09731	2.139556	down	0	-0.81035	-1.09731	C4orf3
224875_at	-1.51455	-0.59889	1.514551	down	-1.96219	-0.97246	1.962186	down	0	-0.59889	-0.97246	C5orf24
205199_at	-1.92592	-0.94554	1.925915	down	-2.39351	-1.25913	2.393508	down	0	-0.94554	-1.25913	CA9

64408_s_at	-1.77359	-0.82667	1.773588	down	-1.76563	-0.82019	1.765635	down	0	-0.82667	-0.82019	CALML4
1568600_at	-1.90276	-0.92809	1.902758	down	-1.94166	-0.95729	1.941658	down	0	-0.92809	-0.95729	CALML4
229549_at	-2.19972	-1.13732	2.199722	down	-1.6623	-0.73318	1.662296	down	0	-1.13732	-0.73318	CALU
226382_at	-1.53649	-0.61964	1.53649	down	-1.54427	-0.62692	1.544269	down	0	-0.61964	-0.62692	CAMK1D
1552701_a_at	-1.51258	-0.59701	1.512576	down	-2.60954	-1.3838	2.609542	down	0	-0.59701	-1.3838	CARD16
207006_s_at	-1.51848	-0.60263	1.518483	down	-1.51848	-0.60263	1.518483	down	0	-0.60263	-0.60263	CCDC106
228081_at	-1.52475	-0.60857	1.524749	down	-1.52475	-0.60857	1.524749	down	0	-0.60857	-0.60857	CCNG2
217523_at	-1.54317	-0.62589	1.543165	down	-1.73188	-0.79234	1.731885	down	0	-0.62589	-0.79234	CD44
201005_at	-1.69321	-0.75976	1.693208	down	-1.71919	-0.78172	1.719185	down	0	-0.75976	-0.78172	CD9
209112_at	-1.51395	-0.59832	1.513955	down	-1.66823	-0.73832	1.668228	down	0	-0.59832	-0.73832	CDKN1B
209172_s_at	-1.55736	-0.6391	1.557358	down	-1.51664	-0.60088	1.516642	down	0	-0.6391	-0.60088	CENPF
222848_at	-1.60512	-0.68268	1.60512	down	-1.55081	-0.63302	1.550808	down	0	-0.68268	-0.63302	CENPK
239322_at	-1.7774	-0.82977	1.777399	down	-1.72186	-0.78397	1.72186	down	0	-0.82977	-0.78397	CEP41
209763_at	-2.10335	-1.07269	2.103347	down	-1.99245	-0.99454	1.992447	down	0	-1.07269	-0.99454	CHRDL1
226686_at	-1.6075	-0.68482	1.607497	down	-1.8038	-0.85104	1.803801	down	0	-0.68482	-0.85104	CISD2
241696_at	-1.54326	-0.62598	1.543261	down	-1.91026	-0.93377	1.91026	down	0	-0.62598	-0.93377	CNTLN
206497_at	-1.90286	-0.92817	1.902863	down	-2.1221	-1.08549	2.122095	down	0	-0.92817	-1.08549	COA1
202311_s_at	-2.23578	-1.16078	2.235778	down	-2.46612	-1.30224	2.466123	down	0	-1.16078	-1.30224	COL1A1
213846_at	-1.50822	-0.59284	1.508216	down	-1.64134	-0.71488	1.641344	down	0	-0.59284	-0.71488	COX7C
205832_at	-2.01715	-1.01232	2.017152	down	-2.66247	-1.41276	2.662467	down	0	-1.01232	-1.41276	CPA4
217564_s_at	-2.12998	-1.09084	2.129979	down	-1.81051	-0.8564	1.810514	down	0	-1.09084	-0.8564	CPS1
238914_at	-1.59251	-0.67131	1.592514	down	-2.1329	-1.09281	2.132897	down	0	-0.67131	-1.09281	DCC
212690_at	-1.68118	-0.74947	1.681179	down	-1.54926	-0.63158	1.549261	down	0	-0.74947	-0.63158	DDHD2
218362_s_at	-1.52091	-0.60493	1.520906	down	-1.5917	-0.67057	1.591704	down	0	-0.60493	-0.67057	DIS3
1556821_x_at	-1.56404	-0.64528	1.564041	down	-1.56404	-0.64528	1.564041	down	0	-0.64528	-0.64528	DLEU2
229588_at	-1.5482	-0.63059	1.548195	down	-1.78135	-0.83297	1.781351	down	0	-0.63059	-0.83297	DNAJC10
201843_s_at	-2.1422	-1.09909	2.142202	down	-2.33589	-1.22397	2.335889	down	0	-1.09909	-1.22397	EFEMP1
230479_at	-1.77526	-0.82803	1.775256	down	-1.77526	-0.82803	1.775256	down	0	-0.82803	-0.82803	EIF3F
213570_at	-1.51162	-0.5961	1.511624	down	-1.8415	-0.88088	1.841496	down	0	-0.5961	-0.88088	EIF4E2
230178_s_at	-1.51473	-0.59906	1.514727	down	-1.68711	-0.75456	1.687113	down	0	-0.59906	-0.75456	ELP2
1569349_at	-1.50418	-0.58898	1.504184	down	-1.50418	-0.58898	1.504184	down	0	-0.58898	-0.58898	EMSY
204161_s_at	-1.56496	-0.64613	1.564963	down	-1.73161	-0.79211	1.731609	down	0	-0.64613	-0.79211	ENPP4
227803_at	-1.67406	-0.74335	1.674056	down	-2.01872	-1.01344	2.018717	down	0	-0.74335	-1.01344	ENPP5
237054_at	-1.89966	-0.92574	1.899658	down	-2.35141	-1.23353	2.35141	down	0	-0.92574	-1.23353	ENPP5
1562921_at	-1.51634	-0.60059	1.516339	down	-1.58213	-0.66187	1.58213	down	0	-0.60059	-0.66187	EP300-AS1
206070_s_at	-1.94139	-0.95709	1.941392	down	-2.05777	-1.04108	2.057766	down	0	-0.95709	-1.04108	EPHA3
231926_at	-1.50646	-0.59116	1.506455	down	-1.67816	-0.74688	1.678165	down	0	-0.59116	-0.74688	EPS15L1
226418_at	-1.55412	-0.6361	1.554117	down	-1.55412	-0.6361	1.554117	down	0	-0.6361	-0.6361	ERGIC2
224698_at	-1.54027	-0.62319	1.540274	down	-1.54027	-0.62319	1.540274	down	0	-0.62319	-0.62319	ESYT2
203989_x_at	-1.55083	-0.63304	1.550832	down	-1.96302	-0.97308	1.963023	down	0	-0.63304	-0.97308	F2R
213506_at	-1.50939	-0.59397	1.509393	down	-1.82997	-0.87182	1.829974	down	0	-0.59397	-0.87182	F2RL1
230147_at	-1.55788	-0.63958	1.557879	down	-1.58145	-0.66125	1.581449	down	0	-0.63958	-0.66125	F2RL2
228441_s_at	-1.5367	-0.61984	1.536704	down	-1.82661	-0.86917	1.826606	down	0	-0.61984	-0.86917	FAM160A1
235850_at	-1.7047	-0.76951	1.704696	down	-1.75146	-0.80856	1.751461	down	0	-0.76951	-0.80856	FAM162A

36612_at	-2.01993	-1.0143	2.019928	down	-2.43153	-1.28186	2.431527	down	0	-1.0143	-1.28186	FAM168A
224973_at	-1.50936	-0.59394	1.509359	down	-1.64288	-0.71623	1.642879	down	0	-0.59394	-0.71623	FAM46A
226811_at	-1.73645	-0.79614	1.736448	down	-1.92451	-0.94449	1.924513	down	0	-0.79614	-0.94449	FAM46C
228987_at	-1.91419	-0.93673	1.914188	down	-1.57191	-0.65251	1.571906	down	0	-0.93673	-0.65251	FAM49B
226062_x_at	-1.51958	-0.60367	1.519581	down	-1.64349	-0.71676	1.643487	down	0	-0.60367	-0.71676	FAM63A
235125_x_at	-1.52991	-0.61345	1.529914	down	-1.70903	-0.77318	1.70903	down	0	-0.61345	-0.77318	FAM73A
235318_at	-1.66302	-0.7338	1.663017	down	-2.05731	-1.04076	2.057312	down	0	-0.7338	-1.04076	FBN1
221123_x_at	-1.50359	-0.58842	1.503595	down	-1.56494	-0.64611	1.56494	down	0	-0.58842	-0.64611	FBXO16///ZNF395
232693_s_at	-1.58804	-0.66725	1.588044	down	-1.68266	-0.75075	1.682664	down	0	-0.66725	-0.75075	FBXO16///ZNF395
1559096_x_at	-1.59847	-0.67669	1.598469	down	-1.70845	-0.77268	1.708446	down	0	-0.67669	-0.77268	FBXO9
235311_at	-1.58872	-0.66787	1.588721	down	-1.86201	-0.89686	1.862009	down	0	-0.66787	-0.89686	FKBP14
223667_at	-1.83911	-0.87901	1.839108	down	-2.22977	-1.15689	2.229767	down	0	-0.87901	-1.15689	FKBP7
1558199_at	-1.55128	-0.63346	1.551283	down	-1.75359	-0.81031	1.753585	down	0	-0.63346	-0.81031	FN1
207653_at	-1.55375	-0.63575	1.55375	down	-1.55375	-0.63575	1.55375	down	0	-0.63575	-0.63575	FOXD2
243409_at	-1.64522	-0.71828	1.645221	down	-1.77892	-0.831	1.778921	down	0	-0.71828	-0.831	FOXL1
235535_x_at	-1.57515	-0.65549	1.575148	down	-1.57515	-0.65549	1.575148	down	0	-0.65549	-0.65549	FRG1///FRG1BP///FRG1CP
230904_at	-1.67839	-0.74708	1.678395	down	-1.61946	-0.69551	1.619456	down	0	-0.74708	-0.69551	FSD1L
236035_at	-1.66701	-0.73727	1.667014	down	-1.66701	-0.73727	1.667014	down	0	-0.73727	-0.73727	FZD1
204875_s_at	-1.50886	-0.59346	1.508859	down	-1.59322	-0.67195	1.593223	down	0	-0.59346	-0.67195	GMDS
242404_at	-1.82244	-0.86587	1.822444	down	-1.82244	-0.86587	1.822444	down	0	-0.86587	-0.86587	GNB5
213212_x_at	-1.51211	-0.59656	1.512106	down	-1.61944	-0.6955	1.61944	down	0	-0.59656	-0.6955	GOLGA6L4///GOLGA6L5P
243880_at	-1.76339	-0.81835	1.763387	down	-1.68459	-0.7524	1.684589	down	0	-0.81835	-0.7524	GOSR2
219313_at	-1.9285	-0.94748	1.9285	down	-1.66701	-0.73727	1.667014	down	0	-0.94748	-0.73727	GRAMD1C
240452_at	-1.5936	-0.67229	1.593603	down	-1.54683	-0.62931	1.546826	down	0	-0.67229	-0.62931	GSPT1
215599_at	-1.93964	-0.95579	1.939636	down	-1.96419	-0.97393	1.964188	down	0	-0.95579	-0.97393	GUSBP3
213054_at	-1.84358	-0.88251	1.843582	down	-1.5177	-0.60189	1.517699	down	0	-0.88251	-0.60189	HAUS5
227350_at	-1.86416	-0.89852	1.864159	down	-1.71598	-0.77903	1.715982	down	0	-0.89852	-0.77903	HELLS
208576_s_at	-2.06254	-1.04442	2.062541	down	-1.8115	-0.85719	1.811504	down	0	-1.04442	-0.85719	HIST1H3B
238900_at	-1.5622	-0.64358	1.562196	down	-1.59561	-0.67411	1.595607	down	0	-0.64358	-0.67411	HLA-DRB1///HLA-DRB3
225786_at	-1.8609	-0.896	1.860901	down	-1.58739	-0.66666	1.587394	down	0	-0.896	-0.66666	HNRNPU
235603_at	-1.61642	-0.69281	1.616424	down	-1.80151	-0.8492	1.801507	down	0	-0.69281	-0.8492	HNRNPU
244442_at	-1.52018	-0.60424	1.520178	down	-1.51367	-0.59805	1.513666	down	0	-0.60424	-0.59805	HORMAD2
230031_at	-1.63297	-0.7075	1.632967	down	-1.73712	-0.7967	1.737121	down	0	-0.7075	-0.7967	HSPA5
242639_at	-1.99369	-0.99544	1.993693	down	-1.65001	-0.72248	1.650014	down	0	-0.99544	-0.72248	ICE2
213931_at	-1.67725	-0.7461	1.677254	down	-1.80022	-0.84818	1.800224	down	0	-0.7461	-0.84818	ID2///ID2B
236823_at	-1.88591	-0.91526	1.885907	down	-1.88591	-0.91526	1.885907	down	0	-0.91526	-0.91526	IDS
204415_at	-2.11077	-1.07777	2.11077	down	-2.131	-1.09153	2.130995	down	0	-1.07777	-1.09153	IFI6
210095_s_at	-1.56667	-0.6477	1.566667	down	-1.57268	-0.65323	1.572684	down	0	-0.6477	-0.65323	IGFBP3
212143_s_at	-1.89378	-0.92127	1.893781	down	-2.01906	-1.01368	2.019058	down	0	-0.92127	-1.01368	IGFBP3
1562848_at	-1.67652	-0.74547	1.676522	down	-1.63096	-0.70572	1.63096	down	0	-0.74547	-0.70572	IST1
201474_s_at	-1.527	-0.6107	1.527004	down	-1.57061	-0.65133	1.570612	down	0	-0.6107	-0.65133	ITGA3
205885_s_at	-1.88473	-0.91436	1.884727	down	-1.61644	-0.69282	1.616439	down	0	-0.91436	-0.69282	ITGA4
213416_at	-1.67503	-0.74419	1.675032	down	-2.24266	-1.16521	2.242657	down	0	-0.74419	-1.16521	ITGA4
223412_at	-1.60812	-0.68538	1.608122	down	-2.11021	-1.07739	2.110213	down	0	-0.68538	-1.07739	KBTBD7

229298_at	-1.81926	-0.86335	1.819257	down	-1.81926	-0.86335	1.819257	down	0	-0.86335	-0.86335	KBTBD7
229970_at	-1.59508	-0.67363	1.595078	down	-1.59508	-0.67363	1.595078	down	0	-0.67363	-0.67363	KBTBD7
229850_at	-1.60044	-0.67847	1.60044	down	-1.82283	-0.86618	1.82283	down	0	-0.67847	-0.86618	KDSR
238659_at	-2.06618	-1.04697	2.066182	down	-1.95786	-0.96928	1.957858	down	0	-1.04697	-0.96928	KIAA0141
213316_at	-1.63321	-0.70771	1.633206	down	-1.80131	-0.84904	1.801306	down	0	-0.70771	-0.84904	KIAA1462
236887_at	-1.71683	-0.77975	1.716832	down	-1.62269	-0.69839	1.622688	down	0	-0.77975	-0.69839	KIN
226158_at	-1.67251	-0.74201	1.67251	down	-1.83306	-0.87426	1.833063	down	0	-0.74201	-0.87426	KLHL24
201795_at	-1.6681	-0.7382	1.668097	down	-1.76147	-0.81678	1.761467	down	0	-0.7382	-0.81678	LBR
225571_at	-1.80146	-0.84917	1.801462	down	-1.69723	-0.76318	1.697226	down	0	-0.84917	-0.76318	LIFR
222456_s_at	-1.75957	-0.81522	1.759568	down	-1.85216	-0.88921	1.852157	down	0	-0.81522	-0.88921	LIMA1
213703_at	-2.18387	-1.12688	2.183866	down	-2.45683	-1.2968	2.456833	down	0	-1.12688	-1.2968	LINC00342
204249_s_at	-2.187	-1.12896	2.187005	down	-2.1903	-1.13113	2.190304	down	0	-1.12896	-1.13113	LMO2
235060_at	-1.58035	-0.66024	1.580347	down	-1.82953	-0.87148	1.829533	down	0	-0.66024	-0.87148	LOC100190986
240467_at	-1.54592	-0.62847	1.545925	down	-1.66044	-0.73156	1.660438	down	0	-0.62847	-0.73156	LOC105373341
235151_at	-1.63886	-0.71269	1.63886	down	-1.98069	-0.986	1.980692	down	0	-0.71269	-0.986	LOC283357
229007_at	-1.65436	-0.72627	1.654357	down	-1.82856	-0.87071	1.82856	down	0	-0.72627	-0.87071	LOC283788
231478_at	-1.5234	-0.6073	1.523404	down	-1.5234	-0.6073	1.523404	down	0	-0.6073	-0.6073	LOC729966///PDE4C
1561180_at	-1.69667	-0.76271	1.696673	down	-1.75906	-0.8148	1.759055	down	0	-0.76271	-0.8148	LRP11
231840_x_at	-1.50211	-0.58699	1.502114	down	-1.61823	-0.69442	1.618229	down	0	-0.58699	-0.69442	LYRM7
221261_x_at	-1.57791	-0.65801	1.577908	down	-1.52804	-0.61168	1.528036	down	0	-0.65801	-0.61168	MAGED4///MAGED4B
223313_s_at	-1.6238	-0.69937	1.623796	down	-1.67247	-0.74198	1.672471	down	0	-0.69937	-0.74198	MAGED4///MAGED4B
228925_at	-1.61323	-0.68995	1.613227	down	-1.56315	-0.64445	1.563147	down	0	-0.68995	-0.64445	MAPKAPK5
1553759_at	-1.5341	-0.61739	1.534097	down	-1.68956	-0.75665	1.689558	down	0	-0.61739	-0.75665	MCM9
202611_s_at	-1.6127	-0.68948	1.612703	down	-1.73736	-0.7969	1.737364	down	0	-0.68948	-0.7969	MED14
202016_at	-1.52778	-0.61143	1.527778	down	-2.11178	-1.07846	2.11178	down	0	-0.61143	-1.07846	MEST
236480_at	-2.03393	-1.02427	2.033932	down	-2.39152	-1.25793	2.391519	down	0	-1.02427	-1.25793	MIR210HG
229734_at	-1.5057	-0.59043	1.505696	down	-1.69517	-0.76143	1.695167	down	0	-0.59043	-0.76143	MIR4697HG
232071_at	-1.6505	-0.7229	1.650496	down	-1.68134	-0.74961	1.681341	down	0	-0.7229	-0.74961	MRPL19
242289_at	-1.64295	-0.71628	1.642946	down	-1.64295	-0.71628	1.642946	down	0	-0.71628	-0.71628	MRPL42
209146_at	-1.52642	-0.61015	1.52642	down	-1.54412	-0.62678	1.544117	down	0	-0.61015	-0.62678	MSMO1
212338_at	-1.58488	-0.66437	1.584876	down	-1.52562	-0.60939	1.525615	down	0	-0.66437	-0.60939	MYO1D
221899_at	-1.50871	-0.59331	1.508709	down	-1.59752	-0.67583	1.597518	down	0	-0.59331	-0.67583	N4BP2L2
201774_s_at	-1.61844	-0.6946	1.618436	down	-1.79683	-0.84545	1.796828	down	0	-0.6946	-0.84545	NCAPD2
200632_s_at	-1.61889	-0.69501	1.618892	down	-1.76478	-0.81949	1.76478	down	0	-0.69501	-0.81949	NDRG1
229235_at	-1.53124	-0.6147	1.531243	down	-1.55779	-0.6395	1.55779	down	0	-0.6147	-0.6395	NFATC2IP
213032_at	-1.65799	-0.72943	1.657989	down	-1.85143	-0.88864	1.851431	down	0	-0.72943	-0.88864	NFIB
235432_at	-1.50858	-0.59319	1.508581	down	-1.86312	-0.89772	1.863115	down	0	-0.59319	-0.89772	NPHP3
215921_at	-1.5982	-0.67645	1.598203	down	-1.52444	-0.60828	1.524439	down	0	-0.67645	-0.60828	NPIPB11///NPIPB3
229956_at	-1.56984	-0.65062	1.569839	down	-1.57359	-0.65406	1.573586	down	0	-0.65062	-0.65406	NR2C1
209505_at	-1.86139	-0.89638	1.861385	down	-2.03769	-1.02693	2.03769	down	0	-0.89638	-1.02693	NR2F1
241962_at	-1.73341	-0.79362	1.733415	down	-1.99117	-0.99361	1.991166	down	0	-0.79362	-0.99361	NT5DC1
1569030_s_at	-1.55135	-0.63352	1.551351	down	-1.58611	-0.66549	1.586108	down	0	-0.63352	-0.66549	NUB1
225438_at	-1.51622	-0.60048	1.516225	down	-1.61073	-0.68771	1.61073	down	0	-0.60048	-0.68771	NUDCD1
213075_at	-1.64399	-0.7172	1.643988	down	-1.67801	-0.74675	1.678008	down	0	-0.7172	-0.74675	OLFML2A

200816_s_at	-1.50204	-0.58693	1.502043	down		-1.54056	-0.62345	1.540559	down	0	-0.58693	-0.62345	PAFAH1B1
230100_x_at	-1.5616	-0.64302	1.561598	down		-1.50209	-0.58697	1.502085	down	0	-0.64302	-0.58697	PAK1
224212_s_at	-1.52871	-0.61231	1.528708	down		-1.52871	-0.61231	1.528708	down	0	-0.61231	-0.61231	PCDHA1///PCDHA10///PCDHA11
231726_at	-1.50769	-0.59234	1.507693	down		-1.74787	-0.80559	1.747866	down	0	-0.59234	-0.80559	PCDHB14
232099_at	-1.5041	-0.5889	1.504098	down		-1.76894	-0.82289	1.768942	down	0	-0.5889	-0.82289	PCDHB16
228905_at	-1.70272	-0.76784	1.70272	down		-1.70887	-0.77304	1.708868	down	0	-0.76784	-0.77304	PCM1
226119_at	-1.69442	-0.7608	1.694424	down		-1.58565	-0.66507	1.585647	down	0	-0.7608	-0.66507	PCMTD1
232521_at	-1.57115	-0.65182	1.571154	down		-1.57115	-0.65182	1.571154	down	0	-0.65182	-0.65182	PCSK7
229453_at	-1.60715	-0.68451	1.607154	down		-1.96805	-0.97677	1.968049	down	0	-0.68451	-0.97677	PDIA3
226452_at	-1.5594	-0.64099	1.559401	down		-1.92952	-0.94824	1.929519	down	0	-0.64099	-0.94824	PDK1
228499_at	-1.80495	-0.85196	1.804949	down		-1.9545	-0.9668	1.954498	down	0	-0.85196	-0.9668	PFKFB4
1558365_at	-2.13061	-1.09127	2.130613	down		-2.42954	-1.28069	2.429544	down	0	-1.09127	-1.28069	PGK1
229705_at	-1.57392	-0.65436	1.573923	down		-1.99718	-0.99797	1.997183	down	0	-0.65436	-0.99797	PIK3C3
239300_at	-1.57663	-0.65685	1.576631	down		-1.60698	-0.68435	1.606979	down	0	-0.65685	-0.68435	PIK3C3
213700_s_at	-1.50055	-0.58549	1.500547	down		-1.50055	-0.58549	1.500547	down	0	-0.58549	-0.58549	PKM
238992_at	-1.50264	-0.5875	1.502643	down		-1.64078	-0.71438	1.640778	down	0	-0.5875	-0.71438	POLI
214978_s_at	-1.51806	-0.60223	1.518058	down		-1.86112	-0.89617	1.861122	down	0	-0.60223	-0.89617	PPFIA4
203407_at	-2.25697	-1.17439	2.256974	down		-3.29695	-1.72113	3.296954	down	0	-1.17439	-1.72113	PPL
201407_s_at	-1.54794	-0.63035	1.547939	down		-1.71707	-0.77995	1.717074	down	0	-0.63035	-0.77995	PPP1CB
201408_at	-1.68433	-0.75218	1.684333	down		-2.08821	-1.06227	2.088212	down	0	-0.75218	-1.06227	PPP1CB
214917_at	-1.75382	-0.8105	1.753822	down		-1.76093	-0.81633	1.760926	down	0	-0.8105	-0.81633	PRKAA1
225984_at	-1.65289	-0.72499	1.652893	down		-1.52356	-0.60745	1.523563	down	0	-0.72499	-0.60745	PRKAA1
238441_at	-1.60667	-0.68408	1.606672	down		-1.62481	-0.70027	1.624811	down	0	-0.68408	-0.70027	PRKAA2
219383_at	-1.89067	-0.9189	1.890667	down		-1.72073	-0.78302	1.72073	down	0	-0.9189	-0.78302	PRR5L
241774_at	-2.34723	-1.23096	2.347229	down		-2.12761	-1.08923	2.127607	down	0	-1.23096	-1.08923	PSMA3-AS1
227944_at	-1.52363	-0.60751	1.52363	down		-1.52896	-0.61255	1.528962	down	0	-0.60751	-0.61255	PTPN3
225396_at	-1.5059	-0.59063	1.505899	down		-1.62214	-0.6979	1.622138	down	0	-0.59063	-0.6979	RBBP4
228455_at	-1.91221	-0.93524	1.912207	down		-1.51497	-0.59929	1.514969	down	0	-0.93524	-0.59929	RBM15
205158_at	-1.71345	-0.77691	1.713455	down		-1.63437	-0.70873	1.634366	down	0	-0.77691	-0.70873	RNASE4
213397_x_at	-2.20659	-1.14182	2.206586	down		-2.41841	-1.27406	2.418408	down	0	-1.14182	-1.27406	RNASE4
238949_at	-1.59512	-0.67366	1.595118	down		-1.74374	-0.80218	1.743737	down	0	-0.67366	-0.80218	RNF145
1556062_at	-1.77622	-0.82881	1.776222	down		-1.81363	-0.85888	1.813631	down	0	-0.82881	-0.85888	RPP30
209006_s_at	-1.70075	-0.76617	1.700749	down		-1.52504	-0.60885	1.525044	down	0	-0.76617	-0.60885	RSRP1
219957_at	-1.5444	-0.62704	1.544396	down		-1.50646	-0.59116	1.506455	down	0	-0.62704	-0.59116	RUFY2
219691_at	-1.59416	-0.67279	1.594155	down		-1.54881	-0.63116	1.548812	down	0	-0.67279	-0.63116	SAMD9
234987_at	-1.6147	-0.69126	1.614696	down		-1.8903	-0.91862	1.890302	down	0	-0.69126	-0.91862	SAMHD1
200832_s_at	-2.27369	-1.18504	2.273692	down		-2.24835	-1.16886	2.248346	down	0	-1.18504	-1.16886	SCD
202375_at	-1.63039	-0.70521	1.630387	down		-1.57756	-0.65769	1.577559	down	0	-0.70521	-0.65769	SEC24D
215641_at	-1.67374	-0.74308	1.673744	down		-2.1663	-1.11523	2.1663	down	0	-0.74308	-1.11523	SEC24D
1552789_at	-1.54037	-0.62327	1.540367	down		-1.71354	-0.77698	1.713542	down	0	-0.62327	-0.77698	SEC62
1552790_a_at	-1.50967	-0.59423	1.509669	down		-1.559	-0.64062	1.559001	down	0	-0.59423	-0.64062	SEC62
235395_at	-1.53755	-0.62063	1.537546	down		-1.53755	-0.62063	1.537546	down	0	-0.62063	-0.62063	SEC63
221768_at	-1.83077	-0.87245	1.830769	down		-2.0528	-1.0376	2.052804	down	0	-0.87245	-1.0376	SFPQ
1553292_s_at	-2.0201	-1.01443	2.020098	down		-1.64295	-0.71629	1.642946	down	0	-1.01443	-0.71629	SGK494

231938_at	-1.61889	-0.69501	1.618892	down	-1.61889	-0.69501	1.618892	down	0	-0.69501	-0.69501	SGOL1
202856_s_at	-1.63076	-0.70554	1.630759	down	-1.90984	-0.93346	1.909845	down	0	-0.70554	-0.93346	SLC16A3
223698_at	-1.88805	-0.91689	1.888045	down	-1.56361	-0.64488	1.563611	down	0	-0.91689	-0.64488	SLC25A36
201250_s_at	-1.57217	-0.65276	1.572169	down	-1.80537	-0.8523	1.805372	down	0	-0.65276	-0.8523	SLC2A1
202498_s_at	-1.53214	-0.61554	1.532136	down	-1.7129	-0.77644	1.712899	down	0	-0.61554	-0.77644	SLC2A3
202499_s_at	-1.63066	-0.70546	1.630661	down	-2.01043	-1.00751	2.010433	down	0	-0.70546	-1.00751	SLC2A3
243166_at	-1.58099	-0.66083	1.580987	down	-1.78054	-0.83231	1.780538	down	0	-0.66083	-0.83231	SLC30A5
227620_at	-1.78587	-0.83663	1.785874	down	-2.07835	-1.05544	2.07835	down	0	-0.83663	-1.05544	SLC44A1
235725_at	-1.70804	-0.77234	1.708037	down	-1.70804	-0.77234	1.708037	down	0	-0.77234	-0.77234	SMAD4
1565703_at	-1.93867	-0.95507	1.938674	down	-1.59515	-0.67369	1.595153	down	0	-0.95507	-0.67369	SMAD4
236535_at	-1.66741	-0.73761	1.667408	down	-1.82397	-0.86708	1.823969	down	0	-0.73761	-0.86708	SMC6
212579_at	-1.58249	-0.6622	1.582493	down	-1.6499	-0.72238	1.649897	down	0	-0.6622	-0.72238	SMCHD1
212921_at	-1.86534	-0.89944	1.865335	down	-1.50646	-0.59116	1.506455	down	0	-0.89944	-0.59116	SMYD2
1556629_a_at	-1.73994	-0.79904	1.739941	down	-2.057	-1.04054	2.056996	down	0	-0.79904	-1.04054	SNAP25
222286_at	-1.83953	-0.87934	1.839529	down	-1.8331	-0.87428	1.833098	down	0	-0.87934	-0.87428	SNAPC3
229712_at	-2.03234	-1.02315	2.032345	down	-2.15605	-1.10839	2.156048	down	0	-1.02315	-1.10839	SNAPC3
242146_at	-1.60692	-0.6843	1.606918	down	-2.00405	-1.00292	2.004053	down	0	-0.6843	-1.00292	SNRPA1
213364_s_at	-1.57889	-0.65892	1.578895	down	-1.67045	-0.74024	1.670454	down	0	-0.65892	-0.74024	SNX1
232049_at	-1.89201	-0.91992	1.892014	down	-1.86118	-0.89621	1.861176	down	0	-0.91992	-0.89621	SNX2
207711_at	-1.68215	-0.7503	1.682146	down	-1.81337	-0.85867	1.813372	down	0	-0.7503	-0.85867	SOGA1
229075_at	-1.50597	-0.59069	1.50597	down	-1.59087	-0.66982	1.590871	down	0	-0.59069	-0.66982	SPATA5
230885_at	-1.5999	-0.67798	1.599898	down	-1.63375	-0.70818	1.633746	down	0	-0.67798	-0.70818	SPG7
235611_at	-1.58484	-0.66433	1.584837	down	-1.61375	-0.69041	1.613746	down	0	-0.66433	-0.69041	SREK1
200685_at	-1.62067	-0.69659	1.620668	down	-1.68187	-0.75006	1.681866	down	0	-0.69659	-0.75006	SRSF11
213742_at	-1.7484	-0.80604	1.7484	down	-1.78543	-0.83627	1.785431	down	0	-0.80604	-0.83627	SRSF11
238483_at	-1.74642	-0.8044	1.746424	down	-2.26037	-1.17656	2.260368	down	0	-0.8044	-1.17656	SSBP2
238484_s_at	-1.55848	-0.64014	1.55848	down	-1.79687	-0.84549	1.79687	down	0	-0.64014	-0.84549	SSBP2
238861_at	-1.54749	-0.62993	1.547491	down	-1.65223	-0.72442	1.652234	down	0	-0.62993	-0.72442	SSBP2
1558604_a_at	-1.61142	-0.68834	1.611424	down	-1.69303	-0.75961	1.693028	down	0	-0.68834	-0.75961	SSBP2
225435_at	-1.60409	-0.68175	1.60409	down	-1.66709	-0.73733	1.667088	down	0	-0.68175	-0.73733	SSR1
226390_at	-1.772	-0.82538	1.772	down	-1.62564	-0.70101	1.625639	down	0	-0.82538	-0.70101	STARD4
229513_at	-1.55674	-0.63853	1.556745	down	-1.7011	-0.76647	1.701098	down	0	-0.63853	-0.76647	STRBP
215287_at	-1.5694	-0.65021	1.569401	down	-1.77161	-0.82506	1.771613	down	0	-0.65021	-0.82506	STRN
202761_s_at	-2.08828	-1.06232	2.088283	down	-1.73986	-0.79897	1.739858	down	0	-1.06232	-0.79897	SYNE2
228483_s_at	-1.84723	-0.88536	1.847228	down	-2.13436	-1.0938	2.134358	down	0	-0.88536	-1.0938	TAF9B
238067_at	-1.55667	-0.63847	1.556674	down	-1.85467	-0.89117	1.854674	down	0	-0.63847	-0.89117	TBC1D8B
235890_at	-1.63941	-0.71318	1.63941	down	-1.53798	-0.62103	1.537976	down	0	-0.71318	-0.62103	TBL1XR1
201449_at	-1.50456	-0.58934	1.504556	down	-1.98371	-0.9882	1.98371	down	0	-0.58934	-0.9882	TIA1
224560_at	-1.9816	-0.98666	1.981597	down	-2.23237	-1.15858	2.23237	down	0	-0.98666	-1.15858	TIMP2
201150_s_at	-2.31234	-1.20935	2.312337	down	-2.63954	-1.40029	2.639539	down	0	-1.20935	-1.40029	TIMP3
217974_at	-1.60913	-0.68628	1.609133	down	-1.61469	-0.69126	1.614689	down	0	-0.68628	-0.69126	TM7SF3
235798_at	-1.53789	-0.62095	1.537887	down	-1.7403	-0.79934	1.740303	down	0	-0.62095	-0.79934	TMEM170B
228255_at	-1.63677	-0.71086	1.636774	down	-1.67263	-0.74212	1.672631	down	0	-0.71086	-0.74212	TMEM237
234980_at	-1.62791	-0.70302	1.627913	down	-1.56019	-0.64172	1.560193	down	0	-0.70302	-0.64172	TMEM56

237515_at	-2.01951	-1.014	2.019506	down	-1.50759	-0.59225	1.507592	down	0	-1.014	-0.59225	TMEM56		
232023_at	-1.51801	-0.60218	1.518005	down	-1.62817	-0.70325	1.628171	down	0	-0.60218	-0.70325	TMEM67		
237169_at	-1.73134	-0.79189	1.731342	down	-2.21179	-1.14521	2.211785	down	0	-0.79189	-1.14521	TNC		
241858_at	-1.51403	-0.59839	1.514029	down	-1.75193	-0.80895	1.751933	down	0	-0.59839	-0.80895	TNNI3K		
230779_at	-1.56556	-0.64668	1.56556	down	-1.88628	-0.91554	1.886277	down	0	-0.64668	-0.91554	TNRC6B		
230241_at	-1.57285	-0.65338	1.572846	down	-1.77399	-0.827	1.773992	down	0	-0.65338	-0.827	TOR1AIP2		
201691_s_at	-1.93721	-0.95398	1.937206	down	-1.80768	-0.85414	1.807676	down	0	-0.95398	-0.85414	TPD52		
230211_at	-1.74956	-0.80699	1.749557	down	-1.6183	-0.69448	1.618299	down	0	-0.80699	-0.69448	TRIP11		
235561_at	-2.54403	-1.34711	2.544026	down	-2.02943	-1.02107	2.029428	down	0	-1.34711	-1.02107	TXNL1		
238868_at	-1.60692	-0.6843	1.606917	down	-2.31538	-1.21125	2.315384	down	0	-0.6843	-1.21125	UACA		
1555560_at	-2.1536	-1.10675	2.153603	down	-2.36034	-1.23899	2.360339	down	0	-1.10675	-1.23899	UGGT2		
229573_at	-1.74642	-0.8044	1.746416	down	-2.03769	-1.02693	2.037686	down	0	-0.8044	-1.02693	USP9X		
213686_at	-1.61878	-0.69491	1.618784	down	-1.61232	-0.68914	1.61232	down	0	-0.69491	-0.68914	VPS13A		
235625_at	-1.73055	-0.79123	1.730551	down	-2.22683	-1.15499	2.226831	down	0	-0.79123	-1.15499	VPS41		
230267_at	-1.77877	-0.83088	1.77877	down	-2.11434	-1.08021	2.114338	down	0	-0.83088	-1.08021	WSB1		
206698_at	-1.51151	-0.59599	1.511512	down	-1.69761	-0.76351	1.69761	down	0	-0.59599	-0.76351	XK		
242443_at	-1.76256	-0.81768	1.762565	down	-1.60518	-0.68273	1.605179	down	0	-0.81768	-0.68273	ZC3H14		
206169_x_at	-1.53072	-0.61421	1.530725	down	-1.9016	-0.92721	1.901599	down	0	-0.61421	-0.92721	ZC3H7B		
212366_at	-1.5144	-0.59874	1.514398	down	-1.68336	-0.75135	1.683363	down	0	-0.59874	-0.75135	ZNF292		
218149_s_at	-1.58914	-0.66825	1.589144	down	-1.74512	-0.80333	1.74512	down	0	-0.66825	-0.80333	ZNF395		
219848_s_at	-1.58716	-0.66645	1.587162	down	-1.67298	-0.74242	1.672977	down	0	-0.66645	-0.74242	ZNF432		
228630_at	-1.50008	-0.58504	1.500079	down	-1.50906	-0.59365	1.509057	down	0	-0.58504	-0.59365	ZNF84		
237335_at	-1.56421	-0.64544	1.564215	down	-1.84351	-0.88246	1.843512	down	0	-0.64544	-0.88246	ZP1		
209446_s_at	-1.80519	-0.85215	1.80519	down	-1.7824	-0.83382	1.782399	down	0	-0.85215	-0.83382			
213500_at	-1.61049	-0.6875	1.610494	down	-1.72554	-0.78705	1.725539	down	0	-0.6875	-0.78705			
213605_s_at	-2.10002	-1.0704	2.100021	down	-2.08878	-1.06266	2.088777	down	0	-1.0704	-1.06266			
216147_at	-1.55089	-0.63309	1.550886	down	-1.52349	-0.60738	1.523489	down	0	-0.63309	-0.60738			
217653_x_at	-1.54239	-0.62517	1.542388	down	-1.83336	-0.87449	1.833363	down	0	-0.62517	-0.87449			
31799_at	-1.83827	-0.87835	1.838272	down	-1.91175	-0.9349	1.911752	down	0	-0.87835	-0.9349			
223839_s_at	-1.81599	-0.86075	1.815988	down	-2.05217	-1.03715	2.052173	down	0	-0.86075	-1.03715			
227576_at	-1.6642	-0.73483	1.664199	down	-1.6642	-0.73483	1.664199	down	0	-0.73483	-0.73483			
227663_at	-1.55128	-0.63346	1.551283	down	-1.55128	-0.63346	1.551283	down	0	-0.63346	-0.63346			
229434_at	-1.95419	-0.96657	1.954192	down	-1.91228	-0.93529	1.91228	down	0	-0.96657	-0.93529			
229692_at	-1.54337	-0.62608	1.543366	down	-1.69563	-0.76182	1.695633	down	0	-0.62608	-0.76182			
229795_at	-1.51047	-0.595	1.510469	down	-1.51047	-0.595	1.510469	down	0	-0.595	-0.595			
230927_at	-1.60917	-0.68632	1.60917	down	-1.83853	-0.87856	1.838535	down	0	-0.68632	-0.87856			
231069_at	-1.71304	-0.77656	1.713036	down	-1.76599	-0.82047	1.765985	down	0	-0.77656	-0.82047			
231890_at	-1.72667	-0.78799	1.726666	down	-1.94056	-0.95648	1.940565	down	0	-0.78799	-0.95648			
231979_at	-1.50999	-0.59454	1.509994	down	-1.6446	-0.71774	1.644605	down	0	-0.59454	-0.71774			
232174_at	-2.20539	-1.14103	2.205388	down	-2.05271	-1.03753	2.052707	down	0	-1.14103	-1.03753			
232347_x_at	-1.61128	-0.6882	1.611277	down	-1.62219	-0.69794	1.622187	down	0	-0.6882	-0.69794			
232453_at	-1.81753	-0.86198	1.817533	down	-2.31336	-1.20999	2.313365	down	0	-0.86198	-1.20999			
232541_at	-2.30749	-1.20632	2.307487	down	-1.7754	-0.82814	1.775396	down	0	-1.20632	-0.82814			
232569_at	-1.52651	-0.61023	1.526507	down	-1.67221	-0.74176	1.672214	down	0	-0.61023	-0.74176			

232600_at	-1.82652	-0.8691	1.826524	down	-2.30099	-1.20225	2.300989	down	0	-0.8691	-1.20225	
232889_at	-1.65001	-0.72248	1.650012	down	-1.97831	-0.98427	1.97831	down	0	-0.72248	-0.98427	
233090_at	-1.8893	-0.91786	1.889305	down	-1.72722	-0.78845	1.72722	down	0	-0.91786	-0.78845	
233364_s_at	-1.85848	-0.89413	1.858484	down	-2.0596	-1.04237	2.059602	down	0	-0.89413	-1.04237	
233388_at	-1.6792	-0.74777	1.679196	down	-1.6792	-0.74777	1.679196	down	0	-0.74777	-0.74777	
233518_at	-1.5447	-0.62733	1.544701	down	-1.5194	-0.6035	1.5194	down	0	-0.62733	-0.6035	
233607_at	-1.50949	-0.59406	1.509493	down	-1.50949	-0.59406	1.509493	down	0	-0.59406	-0.59406	
234997_x_at	-1.83704	-0.87738	1.837038	down	-1.8091	-0.85527	1.809101	down	0	-0.87738	-0.85527	
235028_at	-3.62515	-1.85804	3.625148	down	-2.64837	-1.40511	2.648373	down	0	-1.85804	-1.40511	
235159_at	-1.74716	-0.80501	1.747162	down	-1.99152	-0.99387	1.991522	down	0	-0.80501	-0.99387	
235286_at	-1.88015	-0.91085	1.880152	down	-1.85148	-0.88868	1.851477	down	0	-0.91085	-0.88868	
235419_at	-1.57454	-0.65493	1.574541	down	-2.01652	-1.01187	2.016524	down	0	-0.65493	-1.01187	
235513_at	-1.68459	-0.7524	1.68459	down	-1.70591	-0.77054	1.705914	down	0	-0.7524	-0.77054	
235581_at	-1.8284	-0.87058	1.828404	down	-1.81891	-0.86307	1.818907	down	0	-0.87058	-0.86307	
235629_at	-2.13412	-1.09364	2.13412	down	-3.20185	-1.6789	3.201845	down	0	-1.09364	-1.6789	
235693_at	-1.86034	-0.89557	1.860344	down	-1.51842	-0.60257	1.518423	down	0	-0.89557	-0.60257	
235803_at	-1.52046	-0.60451	1.520462	down	-2.11535	-1.0809	2.115349	down	0	-0.60451	-1.0809	
236511_at	-1.59273	-0.67151	1.592734	down	-1.59273	-0.67151	1.592734	down	0	-0.67151	-0.67151	
238146_at	-1.54578	-0.62833	1.545776	down	-1.79352	-0.84279	1.793517	down	0	-0.62833	-0.84279	
238299_at	-1.55227	-0.63438	1.552271	down	-1.57389	-0.65434	1.573892	down	0	-0.63438	-0.65434	
238718_at	-1.65855	-0.72993	1.658553	down	-1.92985	-0.94849	1.929851	down	0	-0.72993	-0.94849	
238774_at	-2.11792	-1.08265	2.117921	down	-1.60518	-0.68273	1.605179	down	0	-1.08265	-0.68273	
239184_at	-1.51652	-0.60076	1.516518	down	-1.95311	-0.96577	1.953107	down	0	-0.60076	-0.96577	
239516_at	-2.15971	-1.11084	2.159708	down	-1.88011	-0.91081	1.880107	down	0	-1.11084	-0.91081	
239735_at	-1.58176	-0.66153	1.581763	down	-1.58176	-0.66153	1.581763	down	0	-0.66153	-0.66153	
240165_at	-1.64872	-0.72135	1.648719	down	-1.85891	-0.89445	1.858906	down	0	-0.72135	-0.89445	
241864_x_at	-1.68935	-0.75646	1.689346	down	-1.9611	-0.97166	1.961096	down	0	-0.75646	-0.97166	
241885_at	-1.54159	-0.62442	1.541587	down	-1.54159	-0.62442	1.541587	down	0	-0.62442	-0.62442	
241991_at	-1.56015	-0.64168	1.560149	down	-1.50949	-0.59406	1.509493	down	0	-0.64168	-0.59406	
242052_at	-2.3525	-1.2342	2.352505	down	-1.73994	-0.79904	1.739941	down	0	-1.2342	-0.79904	
242068_at	-1.99273	-0.99475	1.99273	down	-1.54255	-0.62532	1.542552	down	0	-0.99475	-0.62532	
242233_at	-1.55437	-0.63633	1.554369	down	-1.75875	-0.81455	1.75875	down	0	-0.63633	-0.81455	
242428_at	-1.55676	-0.63854	1.556758	down	-1.52544	-0.60922	1.525437	down	0	-0.63854	-0.60922	
242471_at	-3.1728	-1.66576	3.172802	down	-2.41896	-1.27439	2.418957	down	0	-1.66576	-1.27439	
242476_at	-1.88686	-0.91599	1.886863	down	-1.56404	-0.64528	1.564042	down	0	-0.91599	-0.64528	
242606_at	-1.76148	-0.81679	1.76148	down	-1.88878	-0.91745	1.888777	down	0	-0.81679	-0.91745	
242607_at	-2.04891	-1.03485	2.048907	down	-1.67165	-0.74127	1.671651	down	0	-1.03485	-0.74127	
242688_at	-1.76231	-0.81747	1.762311	down	-1.53345	-0.61678	1.533451	down	0	-0.81747	-0.61678	
242691_at	-1.52749	-0.61116	1.52749	down	-1.52749	-0.61116	1.52749	down	0	-0.61116	-0.61116	
242725_at	-1.65104	-0.72337	1.651037	down	-1.82629	-0.86892	1.826294	down	0	-0.72337	-0.86892	
242772_x_at	-1.52796	-0.61161	1.527964	down	-1.52796	-0.61161	1.527964	down	0	-0.61161	-0.61161	
243023_at	-2.11398	-1.07996	2.113979	down	-2.11398	-1.07996	2.113979	down	0	-1.07996	-1.07996	
243378_at	-1.98793	-0.99126	1.987926	down	-2.2643	-1.17907	2.264301	down	0	-0.99126	-1.17907	
243768_at	-1.66363	-0.73433	1.66363	down	-1.66363	-0.73433	1.66363	down	0	-0.73433	-0.73433	

244014_x_at	-1.53214	-0.61554	1.532136	down	-1.6169	-0.69323	1.616896	down	0	-0.61554	-0.69323	
244503_at	-1.68794	-0.75527	1.687944	down	-2.07145	-1.05064	2.071451	down	0	-0.75527	-1.05064	
1555897_at	-1.65874	-0.73009	1.658744	down	-1.65874	-0.73009	1.658744	down	0	-0.73009	-0.73009	
1555973_at	-1.57115	-0.65182	1.571154	down	-1.59198	-0.67082	1.591982	down	0	-0.65182	-0.67082	
1556081_at	-1.90045	-0.92634	1.900453	down	-2.321	-1.21475	2.320998	down	0	-0.92634	-1.21475	
1558445_at	-1.96431	-0.97402	1.96431	down	-2.12291	-1.08604	2.122905	down	0	-0.97402	-1.08604	
1558605_at	-2.16109	-1.11176	2.161087	down	-1.61299	-0.68973	1.612985	down	0	-1.11176	-0.68973	
1558854_a_at	-1.62003	-0.69602	1.620031	down	-1.62003	-0.69602	1.620031	down	0	-0.69602	-0.69602	
1559006_at	-1.97746	-0.98365	1.977461	down	-2.72075	-1.44401	2.720752	down	0	-0.98365	-1.44401	
1559007_s_at	-1.80863	-0.8549	1.808627	down	-1.72882	-0.78979	1.72882	down	0	-0.8549	-0.78979	
1559776_at	-1.62334	-0.69896	1.623337	down	-1.56619	-0.64726	1.566189	down	0	-0.69896	-0.64726	
1560048_at	-1.87063	-0.90352	1.87063	down	-1.87063	-0.90352	1.87063	down	0	-0.90352	-0.90352	
1562529_s_at	-1.59675	-0.67514	1.596747	down	-1.81253	-0.858	1.812528	down	0	-0.67514	-0.858	
1563467_at	-1.70428	-0.76917	1.704284	down	-1.54102	-0.62388	1.541017	down	0	-0.76917	-0.62388	
1565577_s_at	-1.95966	-0.9706	1.959662	down	-2.2638	-1.17874	2.263795	down	0	-0.9706	-1.17874	
1565886_at	-1.60064	-0.67865	1.600644	down	-1.51403	-0.59839	1.514029	down	0	-0.67865	-0.59839	
1569129_s_at	-1.65758	-0.72908	1.657577	down	-1.66859	-0.73863	1.668587	down	0	-0.72908	-0.73863	
1569167_at	-1.70257	-0.76771	1.702567	down	-1.73122	-0.79179	1.731217	down	0	-0.76771	-0.79179	
213353_at	1.564984	0.646148	1.564984	up	1.591914	0.670762	1.591914	up	0	0.646148	0.670762	ABCA5
208561_at	2.700569	1.433263	2.700569	up	2.891068	1.531602	2.891068	up	0	1.433263	1.531602	ABCC9
208562_s_at	1.800242	0.848191	1.800242	up	1.755287	0.811707	1.755287	up	0	0.848191	0.811707	ABCC9
205566_at	1.824924	0.867836	1.824924	up	1.871669	0.904325	1.871669	up	0	0.867836	0.904325	ABHD2
220606_s_at	1.572217	0.652801	1.572217	up	1.572217	0.652801	1.572217	up	0	0.652801	0.652801	ADPRM
223401_at	1.787679	0.838088	1.787679	up	1.606735	0.684132	1.606735	up	0	0.838088	0.684132	ADPRM
204183_s_at	1.503328	0.58816	1.503328	up	1.555349	0.637238	1.555349	up	0	0.58816	0.637238	ADRBK2
229354_at	1.586358	0.665718	1.586358	up	1.719771	0.782216	1.719771	up	0	0.665718	0.782216	AHRR
220987_s_at	1.566352	0.647409	1.566352	up	1.566352	0.647409	1.566352	up	0	0.647409	0.647409	AKIP1///NUAK2
222383_s_at	1.899509	0.925627	1.899509	up	2.123148	1.086205	2.123148	up	0	0.925627	1.086205	ALOXE3
228415_at	1.786662	0.837267	1.786662	up	1.861887	0.896766	1.861887	up	0	0.837267	0.896766	AP1S2
209913_x_at	1.892622	0.920386	1.892622	up	1.898445	0.924818	1.898445	up	0	0.920386	0.924818	AP5Z1
213419_at	1.726333	0.787711	1.726333	up	1.524208	0.608059	1.524208	up	0	0.787711	0.608059	APBB2
40148_at	1.576239	0.656486	1.576239	up	1.552831	0.634901	1.552831	up	0	0.656486	0.634901	APBB2
213618_at	2.593432	1.374863	2.593432	up	1.952754	0.96551	1.952754	up	0	1.374863	0.96551	ARAP2
1555902_at	1.559431	0.64102	1.559431	up	1.730632	0.791299	1.730632	up	0	0.64102	0.791299	ARMCX5
209824_s_at	1.717092	0.779967	1.717092	up	1.604462	0.682089	1.604462	up	0	0.779967	0.682089	ARNTL
225283_at	1.770431	0.824101	1.770431	up	1.513684	0.598064	1.513684	up	0	0.824101	0.598064	ARRDC4
202672_s_at	4.816474	2.267977	4.816474	up	4.642235	2.214819	4.642235	up	0	2.267977	2.214819	ATF3
1554980_a_at	4.11003	2.039149	4.11003	up	3.734306	1.90084	3.734306	up	0	2.039149	1.90084	ATF3
218987_at	1.534876	0.618122	1.534876	up	1.614903	0.691448	1.614903	up	0	0.618122	0.691448	ATF7IP
212471_at	1.515716	0.6	1.515716	up	1.546693	0.629187	1.546693	up	0	0.6	0.629187	AVL9
203304_at	1.58986	0.6689	1.58986	up	1.662812	0.733625	1.662812	up	0	0.6689	0.733625	BAMBI
207671_s_at	1.737313	0.796858	1.737313	up	1.640628	0.714248	1.640628	up	0	0.796858	0.714248	BEST1
218332_at	1.566254	0.647318	1.566254	up	1.580193	0.660101	1.580193	up	0	0.647318	0.660101	BEX1
210538_s_at	3.146209	1.653615	3.146209	up	3.195384	1.675989	3.195384	up	0	1.653615	1.675989	BIRC3

242579_at	1.533451	0.616782	1.533451	up	1.675466	0.744562	1.675466	up	0	0.616782	0.744562	BMPR1B
1555773_at	1.585075	0.664551	1.585075	up	1.596439	0.674857	1.596439	up	0	0.664551	0.674857	BPIFC
236402_at	1.921019	0.941872	1.921019	up	1.843096	0.882131	1.843096	up	0	0.941872	0.882131	BRAF
227099_s_at	1.886405	0.915639	1.886405	up	1.604184	0.681839	1.604184	up	0	0.915639	0.681839	C11orf96
222767_s_at	1.549938	0.632211	1.549938	up	1.699365	0.764996	1.699365	up	0	0.632211	0.764996	C12orf49
235026_at	1.50058	0.58552	1.50058	up	1.576161	0.656415	1.576161	up	0	0.58552	0.656415	C12orf66
217767_at	2.507407	1.326196	2.507407	up	2.378602	1.250114	2.378602	up	0	1.326196	1.250114	C3
218541_s_at	2.006092	1.004388	2.006092	up	1.828986	0.871044	1.828986	up	0	1.004388	0.871044	C8orf4
232270_at	1.541078	0.62394	1.541078	up	1.616028	0.692452	1.616028	up	0	0.62394	0.692452	C9orf3
209030_s_at	1.686829	0.754314	1.686829	up	1.84304	0.882087	1.84304	up	0	0.754314	0.882087	CADM1
225331_at	1.525491	0.609274	1.525491	up	1.566795	0.647817	1.566795	up	0	0.609274	0.647817	CCDC50
209378_s_at	1.890011	0.918395	1.890011	up	2.119906	1.084	2.119906	up	0	0.918395	1.084	CCSER2
228910_at	1.537667	0.620743	1.537667	up	1.581571	0.661358	1.581571	up	0	0.620743	0.661358	CD82
212942_s_at	2.029405	1.021057	2.029405	up	1.896269	0.923163	1.896269	up	0	1.021057	0.923163	CEMIP
206861_s_at	1.636015	0.710186	1.636015	up	1.712024	0.775703	1.712024	up	0	0.710186	0.775703	CGGBP1
206932_at	2.280811	1.189547	2.280811	up	2.248088	1.168698	2.248088	up	0	1.189547	1.168698	CH25H
219270_at	2.140295	1.09781	2.140295	up	2.174358	1.120589	2.174358	up	0	1.09781	1.120589	CHAC1
219634_at	2.121744	1.085251	2.121744	up	2.133468	1.093201	2.133468	up	0	1.085251	1.093201	CHST11
222549_at	1.972224	0.979823	1.972224	up	1.54943	0.631738	1.54943	up	0	0.979823	0.631738	CLDN1
208096_s_at	2.024985	1.017911	2.024985	up	2.201455	1.138457	2.201455	up	0	1.017911	1.138457	COL21A1
201942_s_at	1.685027	0.752771	1.685027	up	1.842484	0.881652	1.842484	up	0	0.752771	0.881652	CPD
207442_at	2.023101	1.016568	2.023101	up	2.303799	1.204015	2.303799	up	0	1.016568	1.204015	CSF3
206085_s_at	2.173237	1.119845	2.173237	up	2.240097	1.163561	2.240097	up	0	1.119845	1.163561	CTH
217127_at	1.979575	0.98519	1.979575	up	1.898479	0.924844	1.898479	up	0	0.98519	0.924844	CTH
204470_at	2.306001	1.205393	2.306001	up	2.291224	1.196118	2.291224	up	0	1.205393	1.196118	CXCL1
209774_x_at	4.152156	2.053861	4.152156	up	3.799508	1.925813	3.799508	up	0	2.053861	1.925813	CXCL2
207850_at	2.751014	1.459963	2.751014	up	2.42954	1.280683	2.42954	up	0	1.459963	1.280683	CXCL3
214974_x_at	1.810511	0.856397	1.810511	up	1.810511	0.856397	1.810511	up	0	0.856397	0.856397	CXCL5
202859_x_at	8.254849	3.045242	8.254849	up	7.551652	2.916792	7.551652	up	0	3.045242	2.916792	CXCL8
211506_s_at	9.104867	3.186638	9.104867	up	8.555291	3.096817	8.555291	up	0	3.186638	3.096817	CXCL8
221903_s_at	1.910666	0.934075	1.910666	up	1.862456	0.897206	1.862456	up	0	0.934075	0.897206	CYLD
222142_at	1.567429	0.6484	1.567429	up	1.558175	0.639857	1.558175	up	0	0.6484	0.639857	CYLD
205749_at	2.331017	1.22096	2.331017	up	2.341274	1.227294	2.341274	up	0	1.22096	1.227294	CYP1A1
203890_s_at	2.291737	1.196442	2.291737	up	1.64258	0.715964	1.64258	up	0	1.196442	0.715964	DAPK3
201893_x_at	1.650385	0.722803	1.650385	up	1.728692	0.789681	1.728692	up	0	0.722803	0.789681	DCN
211813_x_at	1.829167	0.871187	1.829167	up	1.84608	0.884465	1.84608	up	0	0.871187	0.884465	DCN
209383_at	1.716542	0.779505	1.716542	up	1.660926	0.731988	1.660926	up	0	0.779505	0.731988	DDIT3
212527_at	1.58656	0.665902	1.58656	up	1.535098	0.618331	1.535098	up	0	0.665902	0.618331	DESI1
206806_at	1.842748	0.881859	1.842748	up	1.805186	0.852147	1.805186	up	0	0.881859	0.852147	DGKI
206463_s_at	2.461519	1.299549	2.461519	up	2.516365	1.331341	2.516365	up	0	1.299549	1.331341	DHRS2
214079_at	3.509491	1.811262	3.509491	up	3.551381	1.82838	3.551381	up	0	1.811262	1.82838	DHRS2
200664_s_at	1.995763	0.996941	1.995763	up	2.824477	1.497984	2.824477	up	0	0.996941	1.497984	DNAJB1
200666_s_at	1.664273	0.734892	1.664273	up	2.238227	1.162356	2.238227	up	0	0.734892	1.162356	DNAJB1
212611_at	1.814021	0.859191	1.814021	up	2.069327	1.049162	2.069327	up	0	0.859191	1.049162	DTX4

214446_at	1.675556	0.744639	1.675556	up	1.786642	0.837251	1.786642	up	0	0.744639	0.837251	ELL2
225698_at	1.958952	0.970082	1.958952	up	1.932721	0.950633	1.932721	up	0	0.970082	0.950633	EPB41L4A-AS1
227755_at	2.337646	1.225056	2.337646	up	2.445849	1.290336	2.445849	up	0	1.225056	1.290336	ERN1
215594_at	2.189075	1.130321	2.189075	up	1.8757	0.907429	1.8757	up	0	1.130321	0.907429	ERV9-1
201328_at	1.536993	0.620111	1.536993	up	1.536993	0.620111	1.536993	up	0	0.620111	0.620111	ETS2
224225_s_at	1.990035	0.992794	1.990035	up	1.832505	0.873817	1.832505	up	0	0.992794	0.873817	ETV7
223058_at	1.533356	0.616693	1.533356	up	1.699212	0.764865	1.699212	up	0	0.616693	0.764865	FAM107B
221959_at	1.66883	0.738837	1.66883	up	1.739837	0.798952	1.739837	up	0	0.738837	0.798952	FAM110B
229946_at	1.683603	0.751552	1.683603	up	1.580075	0.659993	1.580075	up	0	0.751552	0.659993	FAM168B
229152_at	2.225464	1.154106	2.225464	up	1.702219	0.767417	1.702219	up	0	1.154106	0.767417	FDCSP
231382_at	1.72235	0.784379	1.72235	up	1.931883	0.950007	1.931883	up	0	0.784379	0.950007	FGF18
204421_s_at	1.805695	0.852554	1.805695	up	1.846205	0.884563	1.846205	up	0	0.852554	0.884563	FGF2
226769_at	1.589844	0.668885	1.589844	up	1.655785	0.727515	1.655785	up	0	0.668885	0.727515	FIBIN
204135_at	2.18896	1.130246	2.18896	up	2.144784	1.100833	2.144784	up	0	1.130246	1.100833	FILIP1L
1554966_a_at	2.447275	1.291176	2.447275	up	2.149028	1.103684	2.149028	up	0	1.291176	1.103684	FILIP1L
213524_s_at	2.184301	1.127172	2.184301	up	2.435544	1.284244	2.435544	up	0	1.127172	1.284244	G0S2
203725_at	2.647621	1.404697	2.647621	up	2.556834	1.354359	2.556834	up	0	1.404697	1.354359	GADD45A
207574_s_at	1.773154	0.826318	1.773154	up	1.885514	0.914958	1.885514	up	0	0.826318	0.914958	GADD45B
209305_s_at	2.081285	1.057475	2.081285	up	1.953699	0.966208	1.953699	up	0	1.057475	0.966208	GADD45B
204121_at	1.7089	0.773068	1.7089	up	1.7089	0.773068	1.7089	up	0	0.773068	0.773068	GADD45G
209602_s_at	1.68151	0.749757	1.68151	up	1.605565	0.683081	1.605565	up	0	0.749757	0.683081	GATA3
209604_s_at	2.1743	1.120551	2.1743	up	2.173592	1.120081	2.173592	up	0	1.120551	1.120081	GATA3
203158_s_at	1.73982	0.798938	1.73982	up	1.905948	0.930509	1.905948	up	0	0.798938	0.930509	GLS
219078_at	1.584254	0.663804	1.584254	up	1.612607	0.689395	1.612607	up	0	0.663804	0.689395	GPATCH2
223789_s_at	1.610995	0.687952	1.610995	up	1.610995	0.687952	1.610995	up	0	0.687952	0.687952	GTPBP2
204235_s_at	1.506834	0.59152	1.506834	up	1.631181	0.705917	1.631181	up	0	0.59152	0.705917	GULP1
205659_at	2.073684	1.052196	2.073684	up	1.816308	0.861009	1.816308	up	0	1.052196	0.861009	HDAC9
230968_at	1.666925	0.737189	1.666925	up	1.616064	0.692484	1.616064	up	0	0.737189	0.692484	HDAC9
232080_at	1.658517	0.729894	1.658517	up	1.623941	0.699499	1.623941	up	0	0.729894	0.699499	HECW2
203394_s_at	1.906617	0.931015	1.906617	up	2.515835	1.331038	2.515835	up	0	0.931015	1.331038	HES1
203395_s_at	1.643638	0.716893	1.643638	up	1.995517	0.996762	1.995517	up	0	0.716893	0.996762	HES1
215071_s_at	2.631793	1.396046	2.631793	up	2.275119	1.185942	2.275119	up	0	1.396046	1.185942	HIST1H2AC
214472_at	3.187055	1.672224	3.187055	up	2.068046	1.048268	2.068046	up	0	1.672224	1.048268	HIST1H2AD///HIST1H3D
214455_at	2.751158	1.460039	2.751158	up	2.247915	1.168587	2.247915	up	0	1.460039	1.168587	HIST1H2BC
209911_x_at	1.970755	0.978748	1.970755	up	1.59968	0.677783	1.59968	up	0	0.978748	0.677783	HIST1H2BD
214502_at	2.841921	1.506867	2.841921	up	1.991983	0.994205	1.991983	up	0	1.506867	0.994205	HIST1H2BJ///LOC105374995
208180_s_at	2.466506	1.302469	2.466506	up	1.883852	0.913685	1.883852	up	0	1.302469	0.913685	HIST1H4H
207046_at	2.078147	1.055298	2.078147	up	1.935679	0.952839	1.935679	up	0	1.055298	0.952839	HIST2H4A///HIST2H4B
204753_s_at	1.909987	0.933563	1.909987	up	2.396212	1.260756	2.396212	up	0	0.933563	1.260756	HLF
204755_x_at	1.516147	0.60041	1.516147	up	1.588587	0.667744	1.588587	up	0	0.60041	0.667744	HLF
203665_at	1.867196	0.900873	1.867196	up	1.72993	0.790713	1.72993	up	0	0.900873	0.790713	HMOX1
200799_at	4.880279	2.286964	4.880279	up	7.555505	2.917528	7.555505	up	0	2.286964	2.917528	HSPA1A///HSPA1B
200800_s_at	2.372331	1.246306	2.372331	up	3.083957	1.624783	3.083957	up	0	1.246306	1.624783	HSPA1A///HSPA1B
202581_at	1.956412	0.96821	1.956412	up	2.684993	1.424918	2.684993	up	0	0.96821	1.424918	HSPA1A///HSPA1B

117_at	4.91763	2.297963	4.91763	up	8.506181	3.088512	8.506181	up	0	2.297963	3.088512	HSPA6
213418_at	11.884	3.570949	11.884	up	22.31338	4.479837	22.31338	up	0	3.570949	4.479837	HSPA6
219284_at	2.564611	1.35874	2.564611	up	2.378847	1.250262	2.378847	up	0	1.35874	1.250262	HSPBAP1
208744_x_at	1.580477	0.66036	1.580477	up	1.943856	0.958921	1.943856	up	0	0.66036	0.958921	HSPH1
202439_s_at	1.638055	0.711984	1.638055	up	1.6483	0.720979	1.6483	up	0	0.711984	0.720979	IDS
217432_s_at	1.735033	0.794963	1.735033	up	1.913808	0.936446	1.913808	up	0	0.794963	0.936446	IDS
210118_s_at	3.70492	1.889442	3.70492	up	3.423411	1.775435	3.423411	up	0	1.889442	1.775435	IL1A
205067_at	4.767488	2.253229	4.767488	up	5.044926	2.334833	5.044926	up	0	2.253229	2.334833	IL1B
39402_at	4.492022	2.167365	4.492022	up	4.78788	2.259387	4.78788	up	0	2.167365	2.259387	IL1B
207526_s_at	4.031469	2.011306	4.031469	up	6.70347	2.744908	6.70347	up	0	2.011306	2.744908	IL1RL1
1555431_a_at	1.542331	0.625113	1.542331	up	1.700831	0.76624	1.700831	up	0	0.625113	0.76624	IL31RA
205207_at	3.278098	1.712859	3.278098	up	3.386521	1.759804	3.386521	up	0	1.712859	1.759804	IL6
210587_at	2.025027	1.017941	2.025027	up	1.824251	0.867304	1.824251	up	0	1.017941	0.867304	INHBE
205376_at	2.133441	1.093182	2.133441	up	2.140502	1.097949	2.140502	up	0	1.093182	1.097949	INPP4B
237056_at	2.2982	1.200504	2.2982	up	1.559147	0.640757	1.559147	up	0	1.200504	0.640757	INSC
202531_at	2.127229	1.088975	2.127229	up	2.065395	1.046418	2.065395	up	0	1.088975	1.046418	IRF1
209743_s_at	1.60197	0.679847	1.60197	up	1.65121	0.723523	1.65121	up	0	0.679847	0.723523	ITCH
209744_x_at	1.647742	0.72049	1.647742	up	1.770269	0.823969	1.770269	up	0	0.72049	0.823969	ITCH
217094_s_at	1.648474	0.721131	1.648474	up	1.724017	0.785774	1.724017	up	0	0.721131	0.785774	ITCH
211339_s_at	1.605287	0.682831	1.605287	up	1.725679	0.787164	1.725679	up	0	0.682831	0.787164	ITK
226267_at	1.542753	0.625507	1.542753	up	1.525068	0.608873	1.525068	up	0	0.625507	0.608873	JDP2
241985_at	1.759215	0.814931	1.759215	up	2.102007	1.071768	2.102007	up	0	0.814931	1.071768	JMY
242855_at	1.504302	0.589095	1.504302	up	1.94389	0.958946	1.94389	up	0	0.589095	0.958946	KCP
211124_s_at	1.692377	0.759051	1.692377	up	1.789196	0.839311	1.789196	up	0	0.759051	0.839311	KITLG
202393_s_at	1.686332	0.753888	1.686332	up	1.64543	0.718465	1.64543	up	0	0.753888	0.718465	KLF10
221985_at	2.035907	1.025672	2.035907	up	1.927689	0.946872	1.927689	up	0	1.025672	0.946872	KLHL24
221986_s_at	1.567742	0.648688	1.567742	up	1.740164	0.799223	1.740164	up	0	0.648688	0.799223	KLHL24
200770_s_at	1.567619	0.648575	1.567619	up	1.567619	0.648575	1.567619	up	0	0.648575	0.648575	LAMC1
227013_at	1.55203	0.634156	1.55203	up	1.567695	0.648644	1.567695	up	0	0.634156	0.648644	LATS2
205266_at	2.792918	1.481773	2.792918	up	2.830286	1.500948	2.830286	up	0	1.481773	1.500948	LIF
1566986_at	2.206442	1.141722	2.206442	up	2.357796	1.237439	2.357796	up	0	1.141722	1.237439	LINC00674
243905_at	1.896481	0.923325	1.896481	up	2.056581	1.040248	2.056581	up	0	0.923325	1.040248	LINC00944
215223_s_at	1.934307	0.951817	1.934307	up	2.030337	1.021719	2.030337	up	0	0.951817	1.021719	LOC100129518///SOD2
236042_at	1.529364	0.612932	1.529364	up	1.662296	0.733177	1.662296	up	0	0.612932	0.733177	LOC100130219
231089_at	1.606852	0.684237	1.606852	up	1.780604	0.832366	1.780604	up	0	0.684237	0.832366	LOC100505664
210365_at	1.559969	0.641517	1.559969	up	2.063821	1.045318	2.063821	up	0	0.641517	1.045318	LOC100506403///RUNX1
229872_s_at	1.877931	0.909144	1.877931	up	1.708517	0.772744	1.708517	up	0	0.909144	0.772744	LOC100996720///LOC100996740///LOC642441///LOC730257
1562274_at	1.79985	0.847876	1.79985	up	1.52638	0.610115	1.52638	up	0	0.847876	0.610115	LOC101928446
231412_at	2.497162	1.32029	2.497162	up	2.287993	1.194083	2.287993	up	0	1.32029	1.194083	LOC101929709
1561667_at	1.806453	0.85316	1.806453	up	1.792225	0.841752	1.792225	up	0	0.85316	0.841752	LOC105370642
1561342_at	1.532909	0.616272	1.532909	up	1.532909	0.616272	1.532909	up	0	0.616272	0.616272	LOC105372742///LOC150005
220009_at	1.563082	0.644393	1.563082	up	1.671566	0.7412	1.671566	up	0	0.644393	0.7412	LONRF3
209840_s_at	2.01244	1.008946	2.01244	up	1.863146	0.89774	1.863146	up	0	1.008946	0.89774	LRRN3
209841_s_at	2.000663	1.000478	2.000663	up	1.976128	0.982676	1.976128	up	0	1.000478	0.982676	LRRN3

201744_s_at	1.631124	0.705867	1.631124	up	1.546536	0.629041	1.546536	up		0	0.705867	0.629041	LUM
205193_at	1.50665	0.591344	1.50665	up	1.50665	0.591344	1.50665	up		0	0.591344	0.591344	MAFF
36711_at	1.67202	0.741592	1.67202	up	1.661974	0.732898	1.661974	up		0	0.741592	0.732898	MAFF
205192_at	2.332789	1.222056	2.332789	up	2.317547	1.212598	2.317547	up		0	1.222056	1.212598	MAP3K14
241541_at	1.553093	0.635144	1.553093	up	1.553093	0.635144	1.553093	up		0	0.635144	0.635144	MIB2
204423_at	1.644347	0.717515	1.644347	up	1.621056	0.696934	1.621056	up		0	0.717515	0.696934	MKLN1
204475_at	2.048603	1.03464	2.048603	up	2.353874	1.235037	2.353874	up		0	1.03464	1.235037	MMP1
204575_s_at	1.641119	0.71468	1.641119	up	1.545369	0.627952	1.545369	up		0	0.71468	0.627952	MMP19
224385_s_at	1.606408	0.683838	1.606408	up	1.630854	0.705628	1.630854	up		0	0.683838	0.705628	MOV10L1
1552489_s_at	1.642239	0.715664	1.642239	up	1.573813	0.654264	1.573813	up		0	0.715664	0.654264	MPP4
230361_at	1.720611	0.782921	1.720611	up	2.057626	1.040981	2.057626	up		0	0.782921	1.040981	MROH1
209928_s_at	1.934817	0.952197	1.934817	up	1.697776	0.763646	1.697776	up		0	0.952197	0.763646	MSC
228846_at	2.238687	1.162653	2.238687	up	2.698633	1.432229	2.698633	up		0	1.162653	1.432229	MXD1
219728_at	1.533734	0.617048	1.533734	up	1.929836	0.948478	1.929836	up		0	0.617048	0.948478	MYOT
1568864_at	1.907769	0.931886	1.907769	up	1.775632	0.828332	1.775632	up		0	0.931886	0.828332	MZF1-AS1
225344_at	1.707667	0.772027	1.707667	up	1.679967	0.748433	1.679967	up		0	0.772027	0.748433	NCOA7
212445_s_at	1.58095	0.660791	1.58095	up	1.526169	0.609915	1.526169	up		0	0.660791	0.609915	NEDD4L
232593_at	6.017009	2.589047	6.017009	up	4.360928	2.124635	4.360928	up		0	2.589047	2.124635	NEURL3
203574_at	2.042602	1.030408	2.042602	up	2.027069	1.019395	2.027069	up		0	1.030408	1.019395	NFIL3
201502_s_at	1.774949	0.827778	1.774949	up	1.684756	0.75254	1.684756	up		0	0.827778	0.75254	NFKBIA
203927_at	1.963297	0.973279	1.963297	up	2.034947	1.024992	2.034947	up		0	0.973279	1.024992	NFKBIE
223218_s_at	1.70233	0.767511	1.70233	up	1.811991	0.857576	1.811991	up		0	0.767511	0.857576	NFKBIZ
206814_at	2.008571	1.00617	2.008571	up	1.780633	0.83239	1.780633	up		0	1.00617	0.83239	NGF
204760_s_at	1.539851	0.622791	1.539851	up	1.656015	0.727716	1.656015	up		0	0.622791	0.727716	NR1D1///THRA
206038_s_at	1.520894	0.604919	1.520894	up	1.848486	0.886344	1.848486	up		0	0.604919	0.886344	NR2C2
206343_s_at	2.176688	1.122134	2.176688	up	2.414901	1.271964	2.414901	up		0	1.122134	1.271964	NRG1
211844_s_at	1.839519	0.879329	1.839519	up	1.521919	0.605892	1.521919	up		0	0.879329	0.605892	NRP2
223510_at	1.537547	0.62063	1.537547	up	1.569403	0.650216	1.569403	up		0	0.62063	0.650216	NRP2
1555366_at	1.511096	0.595595	1.511096	up	1.511096	0.595595	1.511096	up		0	0.595595	0.595595	NSAP11
226351_at	1.507631	0.592284	1.507631	up	1.533878	0.617184	1.533878	up		0	0.592284	0.617184	NSUN4
209552_at	1.581051	0.660884	1.581051	up	1.519056	0.603175	1.519056	up		0	0.660884	0.603175	PAX8
228425_at	3.682738	1.880779	3.682738	up	2.737738	1.452984	2.737738	up		0	1.880779	1.452984	PAX8-AS1
208396_s_at	2.204532	1.140472	2.204532	up	1.654349	0.726264	1.654349	up		0	1.140472	0.726264	PDE1A
213556_at	1.844936	0.883571	1.844936	up	2.151886	1.105602	2.151886	up		0	0.883571	1.105602	PINLYP
219155_at	1.54853	0.630899	1.54853	up	1.6908	0.757706	1.6908	up		0	0.630899	0.757706	PITPNC1
209785_s_at	1.666277	0.736628	1.666277	up	1.510488	0.595015	1.510488	up		0	0.736628	0.595015	PLA2G4C
205479_s_at	2.016689	1.011989	2.016689	up	2.190249	1.131095	2.190249	up		0	1.011989	1.131095	PLAU
211668_s_at	2.132264	1.092386	2.132264	up	2.399822	1.262927	2.399822	up		0	1.092386	1.262927	PLAU
227037_at	1.913057	0.93588	1.913057	up	2.099336	1.069933	2.099336	up		0	0.93588	1.069933	PLD6
201939_at	1.707206	0.771637	1.707206	up	1.575158	0.655497	1.575158	up		0	0.771637	0.655497	PLK2
204285_s_at	2.016744	1.012028	2.016744	up	2.170043	1.117724	2.170043	up		0	1.012028	1.117724	PMAIP1
204286_s_at	1.657625	0.729117	1.657625	up	1.760734	0.816177	1.760734	up		0	0.729117	0.816177	PMAIP1
217875_s_at	1.59478	0.673357	1.59478	up	1.508912	0.593508	1.508912	up		0	0.673357	0.593508	PMEPA1
222449_at	1.520683	0.60472	1.520683	up	1.520683	0.60472	1.520683	up		0	0.60472	0.60472	PMEPA1

222879_s_at	1.600793	0.678787	1.600793	up	1.691842	0.758595	1.691842	up	0	0.678787	0.758595	POLH
242218_at	1.923521	0.943749	1.923521	up	2.131827	1.092091	2.131827	up	0	0.943749	1.092091	PPARD
201489_at	1.523404	0.607299	1.523404	up	1.536184	0.619351	1.536184	up	0	0.607299	0.619351	PPIF
201490_s_at	1.756268	0.812513	1.756268	up	1.647886	0.720616	1.647886	up	0	0.812513	0.720616	PPIF
202014_at	1.678544	0.747211	1.678544	up	1.724504	0.786181	1.724504	up	0	0.747211	0.786181	PPP1R15A
37028_at	1.672772	0.742241	1.672772	up	1.687247	0.754671	1.687247	up	0	0.742241	0.754671	PPP1R15A
220672_at	2.181563	1.125362	2.181563	up	2.323946	1.216577	2.323946	up	0	1.125362	1.216577	PPP4R4
220673_s_at	3.212792	1.683827	3.212792	up	3.060651	1.613839	3.060651	up	0	1.683827	1.613839	PPP4R4
233002_at	2.026257	1.018817	2.026257	up	2.026257	1.018817	2.026257	up	0	1.018817	1.018817	PPP4R4
204748_at	2.142626	1.09938	2.142626	up	2.095296	1.067154	2.095296	up	0	1.09938	1.067154	PTGS2
1554997_a_at	2.290476	1.195648	2.290476	up	2.605419	1.381516	2.605419	up	0	1.195648	1.381516	PTGS2
208010_s_at	1.692094	0.75881	1.692094	up	1.600419	0.67845	1.600419	up	0	0.75881	0.67845	PTPN22
206157_at	1.773517	0.826613	1.773517	up	1.573356	0.653845	1.573356	up	0	0.826613	0.653845	PTX3
1562153_a_at	1.825095	0.867971	1.825095	up	1.652621	0.724756	1.652621	up	0	0.867971	0.724756	PVT1
229894_s_at	1.826681	0.869225	1.826681	up	1.629295	0.704248	1.629295	up	0	0.869225	0.704248	RAB43
219210_s_at	1.695049	0.761327	1.695049	up	1.749168	0.806669	1.749168	up	0	0.761327	0.806669	RAB8B
219494_at	1.517006	0.601226	1.517006	up	1.526345	0.610081	1.526345	up	0	0.601226	0.610081	RAD54B
205205_at	2.221603	1.151601	2.221603	up	1.79726	0.845799	1.79726	up	0	1.151601	0.845799	RELB
227339_at	1.525217	0.609014	1.525217	up	1.512427	0.596865	1.512427	up	0	0.609014	0.596865	RGMB
209545_s_at	1.787152	0.837663	1.787152	up	1.788604	0.838834	1.788604	up	0	0.837663	0.838834	RIPK2
225767_at	1.657624	0.729116	1.657624	up	2.194876	1.134139	2.194876	up	0	0.729116	1.134139	RNA45S5
210056_at	1.613505	0.690198	1.613505	up	1.749809	0.807197	1.749809	up	0	0.690198	0.807197	RND1
210426_x_at	1.903635	0.928757	1.903635	up	2.050767	1.036163	2.050767	up	0	0.928757	1.036163	RORA
210479_s_at	1.727408	0.788609	1.727408	up	1.786068	0.836787	1.786068	up	0	0.788609	0.836787	RORA
204802_at	1.82936	0.871339	1.82936	up	1.526381	0.610115	1.526381	up	0	0.871339	0.610115	RRAD
228186_s_at	2.20634	1.141655	2.20634	up	1.960174	0.970982	1.960174	up	0	1.141655	0.970982	RSPO3
201846_s_at	1.623273	0.698905	1.623273	up	1.59132	0.670224	1.59132	up	0	0.698905	0.670224	RYBP
210592_s_at	1.687978	0.755296	1.687978	up	1.79851	0.846802	1.79851	up	0	0.755296	0.846802	SAT1
213988_s_at	1.739239	0.798456	1.739239	up	1.887175	0.916228	1.887175	up	0	0.798456	0.916228	SAT1
242786_at	1.980814	0.986094	1.980814	up	1.829007	0.87106	1.829007	up	0	0.986094	0.87106	SBF2-AS1
204035_at	9.621045	3.266194	9.621045	up	9.262951	3.211472	9.262951	up	0	3.266194	3.211472	SCG2
203889_at	1.613792	0.690455	1.613792	up	1.658536	0.72991	1.658536	up	0	0.690455	0.72991	SCG5
202062_s_at	1.657458	0.728972	1.657458	up	1.969565	0.977877	1.969565	up	0	0.728972	0.977877	SEL1L
204614_at	3.688716	1.883119	3.688716	up	5.197804	2.377902	5.197804	up	0	1.883119	2.377902	SERPINB2
205576_at	1.647035	0.719871	1.647035	up	1.64972	0.722221	1.64972	up	0	0.719871	0.722221	SERPIND1
223195_s_at	1.687321	0.754734	1.687321	up	1.717889	0.780637	1.717889	up	0	0.754734	0.780637	SESN2
223196_s_at	1.923951	0.944072	1.923951	up	1.867554	0.90115	1.867554	up	0	0.944072	0.90115	SESN2
208381_s_at	1.724114	0.785855	1.724114	up	1.688857	0.756047	1.688857	up	0	0.785855	0.756047	SGPL1
204157_s_at	1.633693	0.708137	1.633693	up	1.633693	0.708137	1.633693	up	0	0.708137	0.708137	SIK3
207038_at	2.714948	1.440925	2.714948	up	2.872218	1.522165	2.872218	up	0	1.440925	1.522165	SLC16A6
230748_at	2.201189	1.138283	2.201189	up	2.267336	1.180998	2.267336	up	0	1.138283	1.180998	SLC16A6
205896_at	2.250945	1.170531	2.250945	up	2.250945	1.170531	2.250945	up	0	1.170531	1.170531	SLC22A4
228181_at	1.523363	0.60726	1.523363	up	1.523363	0.60726	1.523363	up	0	0.60726	0.60726	SLC30A1
200924_s_at	1.628967	0.703958	1.628967	up	1.663235	0.733992	1.663235	up	0	0.703958	0.733992	SLC3A2

223798_at	1.899516	0.925632	1.899516	up	1.860394	0.895608	1.860394	up	0	0.925632	0.895608	SLC41A2
207528_s_at	1.785727	0.836512	1.785727	up	1.989127	0.992135	1.989127	up	0	0.836512	0.992135	SLC7A11
1557078_at	1.55616	0.63799	1.55616	up	1.517273	0.601481	1.517273	up	0	0.63799	0.601481	SLFN5
209897_s_at	1.979295	0.984986	1.979295	up	1.979295	0.984986	1.979295	up	0	0.984986	0.984986	SLIT2
228850_s_at	1.903099	0.928351	1.903099	up	1.823202	0.866474	1.823202	up	0	0.928351	0.866474	SLIT2
236367_at	1.551945	0.634078	1.551945	up	1.552131	0.634251	1.552131	up	0	0.634078	0.634251	SMG7
205443_at	1.585896	0.665298	1.585896	up	1.523261	0.607163	1.523261	up	0	0.665298	0.607163	SNAPC1
223773_s_at	1.562515	0.64387	1.562515	up	1.537971	0.621028	1.537971	up	0	0.64387	0.621028	SNHG12
65588_at	1.603389	0.681125	1.603389	up	1.66846	0.738517	1.66846	up	0	0.681125	0.738517	SNHG17
225220_at	1.600214	0.678265	1.600214	up	1.541023	0.623888	1.541023	up	0	0.678265	0.623888	SNHG8
232964_at	2.906709	1.539387	2.906709	up	3.137171	1.649464	3.137171	up	0	1.539387	1.649464	SPDYE1
229778_at	3.118868	1.641023	3.118868	up	2.549231	1.350062	2.549231	up	0	1.641023	1.350062	SPX
205499_at	1.53476	0.618013	1.53476	up	1.53476	0.618013	1.53476	up	0	0.618013	0.618013	SRPX2
225033_at	1.50278	0.587634	1.50278	up	1.579324	0.659308	1.579324	up	0	0.587634	0.659308	ST3GAL1
227179_at	1.500733	0.585667	1.500733	up	1.652631	0.724764	1.652631	up	0	0.585667	0.724764	STAU2
203439_s_at	1.583314	0.662948	1.583314	up	1.706062	0.77067	1.706062	up	0	0.662948	0.77067	STC2
205342_s_at	1.634645	0.708978	1.634645	up	1.607629	0.684934	1.607629	up	0	0.708978	0.684934	SULT1C2
244070_at	2.263042	1.178263	2.263042	up	2.038729	1.02767	2.038729	up	0	1.178263	1.02767	SYNE1
240206_at	2.312661	1.209554	2.312661	up	2.156181	1.108479	2.156181	up	0	1.209554	1.108479	TARS
225818_s_at	1.711153	0.774969	1.711153	up	1.589972	0.669001	1.589972	up	0	0.774969	0.669001	TBRG1
225819_at	1.537563	0.620646	1.537563	up	1.623092	0.698745	1.623092	up	0	0.620646	0.698745	TBRG1
214704_at	1.569835	0.650613	1.569835	up	1.507262	0.59193	1.507262	up	0	0.650613	0.59193	TCF25
209278_s_at	1.501724	0.586619	1.501724	up	1.545746	0.628304	1.545746	up	0	0.586619	0.628304	TFPI2
201042_at	1.752092	0.809078	1.752092	up	1.571876	0.652487	1.571876	up	0	0.809078	0.652487	TGM2
201108_s_at	1.649992	0.722459	1.649992	up	1.735818	0.795616	1.735818	up	0	0.722459	0.795616	THBS1
227866_at	2.002453	1.001769	2.002453	up	2.002453	1.001769	2.002453	up	0	1.001769	1.001769	TIAM2
212665_at	1.977742	0.983854	1.977742	up	2.021408	1.015361	2.021408	up	0	0.983854	1.015361	TIPARP
228205_at	1.561116	0.642578	1.561116	up	1.599573	0.677687	1.599573	up	0	0.642578	0.677687	TKT
214183_s_at	1.873681	0.905875	1.873681	up	2.05516	1.039251	2.05516	up	0	0.905875	1.039251	TKTL1
220169_at	1.660769	0.731852	1.660769	up	1.624376	0.699885	1.624376	up	0	0.731852	0.699885	TMEM156
234994_at	1.666048	0.73643	1.666048	up	1.624232	0.699758	1.624232	up	0	0.73643	0.699758	TMEM200A
202643_s_at	3.468258	1.794211	3.468258	up	3.415726	1.772193	3.415726	up	0	1.794211	1.772193	TNFAIP3
202644_s_at	3.015002	1.592159	3.015002	up	2.87465	1.523387	2.87465	up	0	1.592159	1.523387	TNFAIP3
209294_x_at	1.662884	0.733687	1.662884	up	1.690302	0.757281	1.690302	up	0	0.733687	0.757281	TNFRSF10B
210405_x_at	1.664054	0.734702	1.664054	up	1.643716	0.716961	1.643716	up	0	0.734702	0.716961	TNFRSF10B
210654_at	1.882287	0.912487	1.882287	up	1.568074	0.648994	1.568074	up	0	0.912487	0.648994	TNFRSF10D
207536_s_at	2.040771	1.029114	2.040771	up	1.779872	0.831774	1.779872	up	0	1.029114	0.831774	TNFRSF9
211786_at	1.626404	0.701686	1.626404	up	1.949944	0.963433	1.949944	up	0	0.701686	0.963433	TNFRSF9
220655_at	1.906413	0.930861	1.906413	up	1.906413	0.930861	1.906413	up	0	0.930861	0.930861	TNIP3
218145_at	1.880246	0.910921	1.880246	up	2.077897	1.055124	2.077897	up	0	0.910921	1.055124	TRIB3
1555788_a_at	1.871679	0.904333	1.871679	up	2.021679	1.015554	2.021679	up	0	0.904333	1.015554	TRIB3
220623_s_at	1.692377	0.759051	1.692377	up	1.849914	0.887458	1.849914	up	0	0.759051	0.887458	TSGA10
223962_at	2.244819	1.166599	2.244819	up	2.197774	1.136043	2.197774	up	0	1.166599	1.136043	TTC29
205807_s_at	1.844904	0.883545	1.844904	up	1.73819	0.797586	1.73819	up	0	0.883545	0.797586	TUFT1

222748_s_at	1.73793	0.79737	1.73793	up	1.522571	0.60651	1.522571	up	0	0.79737	0.60651	TXNL4B
221962_s_at	1.618505	0.694662	1.618505	up	1.615448	0.691935	1.615448	up	0	0.694662	0.691935	UBE2H
1559324_at	2.021165	1.015187	2.021165	up	1.538266	0.621305	1.538266	up	0	1.015187	0.621305	USP32P2
224978_s_at	1.629502	0.704431	1.629502	up	1.841728	0.88106	1.841728	up	0	0.704431	0.88106	USP36
204254_s_at	1.711097	0.774922	1.711097	up	1.839991	0.879698	1.839991	up	0	0.774922	0.879698	VDR
1558212_at	1.555585	0.637457	1.555585	up	1.568459	0.649348	1.568459	up	0	0.637457	0.649348	VLDLR-AS1
217785_s_at	1.603124	0.680886	1.603124	up	1.564945	0.646112	1.564945	up	0	0.680886	0.646112	YKT6
241372_at	1.592643	0.671423	1.592643	up	1.532714	0.616088	1.532714	up	0	0.671423	0.616088	ZC3H6
224915_x_at	1.612846	0.689609	1.612846	up	1.552758	0.634833	1.552758	up	0	0.689609	0.634833	ZFAS1
226227_x_at	1.592966	0.671716	1.592966	up	1.525568	0.609346	1.525568	up	0	0.671716	0.609346	ZFAS1
240809_at	2.485791	1.313705	2.485791	up	2.197978	1.136177	2.197978	up	0	1.313705	1.136177	ZNF295-AS1
229086_at	1.719164	0.781707	1.719164	up	1.746833	0.804742	1.746833	up	0	0.781707	0.804742	ZNF436-AS1
242864_at	1.889578	0.918064	1.889578	up	1.781629	0.833197	1.781629	up	0	0.918064	0.833197	ZNF554
227080_at	1.712514	0.776115	1.712514	up	1.565798	0.646898	1.565798	up	0	0.776115	0.646898	ZNF697
AFFX-M27830_5	2.219857	1.150467	2.219857	up	4.373875	2.128912	4.373875	up	0	1.150467	2.128912	
AFFX-M27830_M	1.635245	0.709507	1.635245	up	2.828988	1.500286	2.828988	up	0	0.709507	1.500286	
216130_at	1.612406	0.689215	1.612406	up	1.653967	0.725931	1.653967	up	0	0.689215	0.725931	
227943_at	1.789846	0.839835	1.789846	up	1.538231	0.621272	1.538231	up	0	0.839835	0.621272	
230574_at	1.584466	0.663997	1.584466	up	1.504552	0.589334	1.504552	up	0	0.663997	0.589334	
236987_at	1.507185	0.591857	1.507185	up	1.500773	0.585706	1.500773	up	0	0.591857	0.585706	
238623_at	1.648197	0.720889	1.648197	up	1.576477	0.656704	1.576477	up	0	0.720889	0.656704	
239715_at	1.728519	0.789537	1.728519	up	1.728519	0.789537	1.728519	up	0	0.789537	0.789537	
242213_at	1.511751	0.59622	1.511751	up	1.627864	0.70298	1.627864	up	0	0.59622	0.70298	
243144_at	1.622242	0.697989	1.622242	up	1.622242	0.697989	1.622242	up	0	0.697989	0.697989	
243955_at	1.524072	0.607931	1.524072	up	1.69005	0.757066	1.69005	up	0	0.607931	0.757066	
244247_at	1.800945	0.848754	1.800945	up	1.51444	0.598784	1.51444	up	0	0.848754	0.598784	
1564807_at	2.965642	1.568345	2.965642	up	1.71893	0.781511	1.71893	up	0	1.568345	0.781511	
1566897_at	1.623102	0.698754	1.623102	up	1.53131	0.614767	1.53131	up	0	0.698754	0.614767	
1568513_x_at	2.220907	1.151149	2.220907	up	3.323158	1.732555	3.323158	up	0	1.151149	1.732555	
1569609_at	1.592272	0.671087	1.592272	up	1.72719	0.788427	1.72719	up	0	0.671087	0.788427	
